# mRNA-Encoded Antibodies: An Emerging Paradigm in Antiviral Protection

**DOI:** 10.3390/biom16020297

**Published:** 2026-02-13

**Authors:** Sergey Klotchenko, Marina Plotnikova

**Affiliations:** Smorodintsev Research Institute of Influenza, The Ministry of Health of the Russian Federation, 197022 Saint-Petersburg, Russia

**Keywords:** mRNA-encoded antibodies, mRNA therapeutics, antibody engineering, broadly neutralizing antibodies (bnAbs), passive immunization, lipid nanoparticles (LNPs), in vivo expression, antiviral prophylaxis, viral infections, infectious diseases

## Abstract

Antibodies are a cornerstone of antiviral immunity, yet conventional recombinant antibody production remains costly and time-consuming. mRNA technology, based on synthetic mRNA encapsulated in lipid nanoparticles, offers an alternative strategy by enabling direct in vivo expression of therapeutic antibodies. This review examines recent advances in the development of mRNA-encoded antibodies for antiviral applications. We outline key technological principles, including mRNA construct design, delivery platforms, and pharmacokinetic properties, and compare this approach with established protein-based antibody therapies. We then summarize preclinical studies targeting a broad spectrum of viral pathogens, which collectively demonstrate rapid antibody expression, high serum concentrations, and strong prophylactic and therapeutic efficacy in animal models. Finally, we discuss the translation of this platform toward clinical application, highlighted by the completion of Phase I evaluation of *Moderna* mRNA-1944 against chikungunya virus. Together, these studies position mRNA-encoded antibodies as a flexible and rapidly deployable platform for passive immunization against emerging viral threats.

## 1. Introduction

Antibodies generated by the human immune system in response to infection or vaccination are key defenses against viruses. In recent years, recombinant monoclonal antibodies have entered the biopharmaceutical market as a new class of antiviral drugs. However, their broader use is still limited due to expensive, labor-intensive, and time-consuming manufacturing. Many of these constraints can be bypassed by packaging exogenous synthetic mRNA sequences that encode neutralizing antibodies into lipid nanoparticles (LNPs). This approach provides rapid, efficient, sustained, and safe targeted expression of full-length functional antibodies or their truncated forms directly in the patient. It also allows for expressing alternative antibody formats, such as intracellular or membrane-anchored antibodies, in specific target cells.

Since 2021, thousands of studies have explored mRNA as a platform for vaccines and therapeutics. This review focuses on an emerging paradigm in antiviral therapy that relies on synthetic mRNA encoding recombinant antibodies against target viral pathogens. We describe key aspects of this platform, including mRNA sequence design and optimization, LNP-mediated delivery, and pharmacokinetic advantages that distinguish mRNA-expressed antibodies from their protein-based counterparts. We also overview preclinical studies evaluating mRNA-based therapeutics that encode neutralizing antibodies against a wide range of viruses including HIV-1, hepatitis B virus, Ebola virus, poxviruses, rabies virus, henipaviruses, arboviruses, influenza viruses, coronaviruses, etc. mRNA-encoded antibodies are rapidly expressed, reach high serum concentrations, and show prolonged circulation in the bloodstream, providing robust protection in both prophylactic and therapeutic applications across animal models. To date, the only mRNA-encoded antibody therapeutic that has completed a Phase I clinical trial is *Moderna* mRNA-1944 for chikungunya virus.

Over the past decade, an experimental idea of expressing therapeutic antibodies through exogenous mRNA has rapidly evolved into a functional technological platform for passive immunization. It offers new ways for emergency prophylaxis and treatment of both existing and emerging viral infections, especially when effective conventional vaccines are unavailable.

## 2. Rationale for Using Recombinant Antibodies in Antiviral Therapy

As the leading cause of infectious disease in humans, viruses remain one of the most persistent challenges in global healthcare [1]. Unlike bacterial infections, which can be largely controlled with antibiotics, viral infections still lack broad-spectrum therapeutic drugs. Vaccines are the primary defense against both existing and emerging viruses, yet effective vaccines are still unavailable for many infectious diseases [2].

Based on their mechanism of action, antiviral therapeutics can be categorized into two main groups: virus-targeted drugs that interfere with the viral life cycle, and host-targeted drugs that activate the host immune system to combat infection [3]. An understanding of the viral replication cycle and structural features of the virions has paved the way for developing antiviral drugs that target the function and enzymatic activity of viral proteins [4]. As a result, the vast majority of antimicrobial drugs currently used in clinical practice are chemotherapeutic agents. However, developing them is very time- and labor-consuming, especially considering that viruses are highly likely to acquire drug resistance.

Historically, even before the discovery of antibiotics and vaccines, intravenous injections of serum containing antibodies from recovering humans or animals served as the main therapeutic approach to infectious diseases [5,6]. In recent decades, scientific and technological advances have brought therapeutic recombinant monoclonal antibodies to the biopharmaceutical market as a new class of antiviral drugs [7,8,9]. Antibodies, also called immunoglobulins (Igs), are components of the adaptive immune system, produced in response to viral infections or vaccination [10]. Native antibodies are highly specific antiviral agents, which are generated via a stochastic yet remarkably efficient process of somatic rearrangement [11] and then refined through affinity maturation in germinal centers [12]. Apart from interacting with viral antigens, antibodies can neutralize viruses by blocking their attachment to the cell surface and subsequent cell entry [13]. Using neutralizing antibodies to prevent and treat viral infections is considered a very promising direction in antiviral drug discovery.

There are five classes of immunoglobulins (IgG, IgA, IgM, IgD, and IgE) that differ in their size, molecular charge, amino acid composition, and carbohydrate content. Each antibody isotype contains heavy (HC) and light (LC) chains that form a unique antigen recognition site. Under normal conditions, IgG is the most abundant antibody class in humans, accounting for about 70–75% of all serum antibodies. IgG has a high affinity for specific antigenic determinants or epitopes and thus holds the greatest biotechnological potential [14].

Typically, only a portion of newly generated antibodies exhibits protective activity. The ability to identify and isolate broadly neutralizing immunoglobulins from memory B cells and plasma cells of immune donors, including immunized animals, has enabled an innovative approach that gave rise to a new class of antiviral drugs based on therapeutic antibodies [15,16,17,18,19,20,21,22]. Amino-acid sequences of these antibodies can be genetically engineered to improve their affinity, specificity, stability, reactogenicity, pharmacokinetic properties, effector functions, and tissue permeability [23].

## 3. The Structure and Therapeutic Potential of Recombinant Antibodies

**Figure 1:**
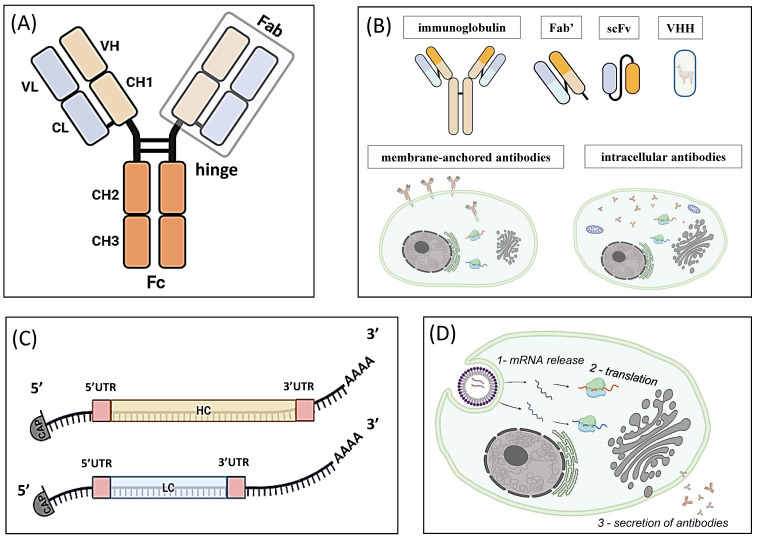
Structure and types of therapeutic antibodies encoded by exogenous mRNAs. (**A**) The structure of full-length class G immunoglobulins (IgG) consists of two light chains (LC, blue) and two heavy chains (HC, orange). Each LC includes a variable (VL) and a constant (CL) domain, and each HC includes one variable (VH) and three constant domains (CH1–CH3). An IgG antibody has an antigen-binding Fab fragment and an effector Fc fragment. (**B**) Truncated forms of antibodies used as therapeutics: Fab′ is a truncated form containing an LC and a fragment of HC (VH and CH1 domains); scFv is a single-chain variable fragment composed of a VH and a VL region connected with a peptide linker; VHH represents a single variable domain (VH) of the camelid heavy-chain-only antibody and can be engineered into homodimers to increase affinity or broaden neutralization; membrane-anchored and intracellular antibodies are examples of antibody formats that can be developed using mRNA technology. (**C**) The general structure of exogenous mRNAs designed to co-express and produce a full-size IgG antibody. (**D**) A schematic overview of how mRNA-encoded therapeutic antibodies are produced in vivo. After administration, the mRNA-lipid nanoparticle complex enters the cell, where the mRNA is released into the cytosol and engages with ribosomes to initiate translation. The antibody chains undergo glycosylation and assembly in the endoplasmic reticulum, followed by processing in the Golgi apparatus [36]. The resulting therapeutic antibodies either remain inside the cell to act on intracellular targets or are secreted into the bloodstream, where they bind to extracellular targets.

Correct immunoglobulin function depends on two structural elements: the antigen-binding Fab fragment and the effector Fc fragment (Figure 1A). The Fab fragment forms a unique antibody paratope composed of the variable domains of heavy and light chains (VH and VL). It enables specific antigen recognition, affinity for the antigen, and protective potential [24]. The effector functions of the Fc fragment are determined by the heavy-chain constant domains (CH) and include antibody-dependent cytotoxicity, cell-mediated phagocytosis, and complement-dependent cytotoxicity [25,26]. Within a species, IgG Fc fragments are highly similar, although allotypic differences create subtle variations between individuals. In contrast, cross-species IgG—for instance, using murine antibodies in humans—can trigger an undesirable immune response [14].

Targeted Fc engineering optimizes the properties of therapeutic antibodies by extending their half-life and modulating effector functions. Mutations such as YTE and LS enhance FcRn binding, prolonging circulation time and reducing dosing frequency. To minimize unwanted immune activity, approaches like the LALA mutations reduce effector functions, which is critical for antagonists or antibody-drug conjugates. These refinements improve the clinical profile and therapeutic risk-benefit ratio for patients [27,28].

The sequences of the variable VH and VL regions from neutralizing immunoglobulins identified in recovering and recovered individuals can be used to create candidate drugs with high therapeutic potential, based on either full-length recombinant antibodies or antibody fragments [29,30,31]. Truncated antibodies (Figure 1B) retain the antigen-binding affinity of the original molecules. They are also more economical to produce and can penetrate tissues and organs more efficiently, while their small size allows access to complex or shielded antigen epitopes. On the other hand, the smaller size of truncated antibodies accelerates their clearance from the bloodstream through renal excretion, which can necessitate higher doses and/or more frequent administration in vivo. Their limited effector functions also reduce reactogenicity [32]. These features typically restrict the application of truncated immunoglobulins to cancer therapy rather than viral infections.

Monoclonal antibodies are an important class of biologics. To date, the US Food and Drug Administration (FDA) has approved over 150 monoclonal antibody–based drugs, with hundreds more in various stages of preclinical and clinical development. For instance, in 2024 alone, the FDA approved 13 new therapeutic antibodies, marking the 30th anniversary of the first recombinant antibody approval [33,34]. In 2025, the agency approved 11 monoclonal antibody–based therapeutics, including clesrovimab for respiratory syncytial virus (RSV) infection [35]. The vast majority of licensed therapeutic antibodies are used to treat autoimmune/inflammatory diseases and cancer.

The tables below list therapeutic monoclonal antibodies with direct antiviral activity that have either been approved for clinical use in different countries (Table 1) or are in late-stage clinical trials as of the beginning of 2026 (Table 2) [34,37,38,39,40,41]. Importantly, most of them are recommended either for emergency use (for instance, against COVID-19) or for the treatment of viral infections when the risks of poor outcomes are extremely high. These antibodies primarily target viral surface proteins to prevent receptor binding and membrane fusion, thereby inhibiting viral entry into the target cell [42]. Antibody cocktails containing two or more different antibodies are often used to improve efficacy and counteract viral evasion driven by mutational variability. For example, this strategy is used to treat COVID-19 [43] and Ebola fever [44], as well as in post-exposure rabies prophylaxis [45].

Antibodies as therapeutic agents may be particularly beneficial when effective vaccine-based prevention is difficult to achieve. For instance, since developing an effective RSV vaccine proved challenging, the FDA has approved three monoclonal antibody therapeutics against RSV infection: palivizumab [46,47], nirsevimab [48,49,50], and clesrovimab [35,51,52,53]. They are now routinely used in clinical practice to prevent complications in high-risk patients, including newborns [50,54,55].

Although therapeutic antibodies are one of the fastest-growing sectors in the pharmaceutical industry, their main challenges remain unresolved. These include the high production cost of the final therapeutic, multi-stage purification process with protocols that vary depending on the antibody, and limited stability of the final formulation [56,57,58,59,60,61]. An alternative approach is delivering genetic information encoding an antibody to target cells as mRNA or DNA [62,63,64,65]. Unlike DNA, mRNA offers a safer and more flexible therapeutic approach. For example, it does not need to enter the nucleus, reduces the risk of insertional mutagenesis, and induces shorter expression of the encoded protein, allowing for adjustable treatment duration.

Nucleic acid-mediated expression not only overcomes the limitations of complex protein production and purification but also helps avoid aberrant post-translational modifications of recombinant antibodies. This strategy can significantly shorten the drug discovery pipeline and accelerate the introduction of therapeutic antibodies against emerging infectious agents into clinical practice, which is a particularly important consideration given the growing incidence of viral epidemics.

**Table 1:** Licensed recombinant antibody therapeutics against viral pathogens.

#	International Nonproprietary Name (Alternative Names)	Brand Name	Target; Mechanism of Action	Indication First Approved or Reviewed	Originator; Country	Developer; Country	Format; Fc Modifications; Reason for Fc Modifications	First US Approval Date	First EU Approval Date	First Global Approval (Country, Year)	Reference
1	Ibalizumab (TMB-355, 5A8)	Trogarzo	CD4 receptor on human T-cells; HIV fusion inhibitor	Treatment of HIV infection	Biogen Idec; US	Taimed Biologics/Theratechnologies; Taiwan/Canada	Humanized IgG4 kappa; None	06.03.2018	26.09.2019; withdrawn 01.01.2023	US, 2018	[66]
2	Atoltivimab/Maftivimab/Odesivimab (REGN-EB3; REGN3470/3471/3479)	Inmazeb	Ebola virus glycoprotein (GP); Virus internalisation inhibitor	Treatment of Ebola virus infection	Regeneron Pharmaceuticals; US	NIAID/Regeneron Pharmaceutical; US	Mixture of 3 human IgG1 kappa; None	14.10.2020	NA	US, 2020	[67]
3	Ansuvimab (EVB114, mAb114)	Ebanga	Ebola virus glycoprotein (GP); Virus internalisation inhibitor	Treatment of Ebola virus infection	Humabs BioMed; Switzerland	Ridgeback Biotherapeutics; US	Human IgG1 kappa; None	21.12.2020	NA	US, 2020	[68]
4	RAB-1 (SII RMAb, 17C7)	RabiShield	Rabies virus glycoprotein (G protein); Virus internalisation inhibitor	Post-exposure prophylaxis of rabies	CDC; US	Serum Institute of India; India	Human IgG1; None	NA	NA	India, 2016	[69]
5	Docaravimab/Miromavimab (M777-16-3/MAb 62-71-3)	TwinRab	Rabies virus glycoprotein (G protein); Virus internalisation inhibitor	Post-exposure prophylaxis of rabies	Zydus Cadila; India	Zydus Cadila; India	Mixture of 2 murine mIgG2b and mIgG1 kappa; None	NA	NA	India, 2019	[70]
6	Ormutivimab (NM-57)	rhRIG	Rabies virus glycoprotein (G protein); Virus internalisation inhibitor	Post-exposure prophylaxis of rabies	Thomas Jefferson University; US	Molecular Targeting Technologies/North China Pharmaceutical Corporation; China	Human IgG1 lambda2; None	NA	NA	China, 2022	[71]
7	Mazorelvimab/Zamerovimab (SYN023; CTB011/CTB012)	Krebi	Rabies virus glycoprotein (G protein); Virus internalisation inhibitor	Post-exposure prophylaxis of rabies	Synermore Biologics; Taiwan	Synermore Biologics; Taiwan	Mixture of 2 humanized IgG1 kappa; None	NA	NA	China, 2024	[72]
8	Palivizumab (MEDI-493)	Synagis	Respiratory syncytial virus F glycoprotein; Viral fusion protein inhibitors	Prevention of respiratory syncytial virus infection	MedImmune; US	MedImmune/AbbVie/AstraZeneca; US/UK	Humanized IgG1 kappa; None	19.06.1998	13.08.1999	US, 1998	[46]
9	Nirsevimab (MEDI8897)	Beyfortus	Respiratory syncytial virus F glycoprotein; Viral fusion protein inhibitor	Prevention of respiratory syncytial virus infection	AIMM Therapeutics; Netherlands	AstraZeneca/Sanofi; UK/France	Human IgG1 kappa; M252Y/S254T/T256E (YTE); Extends half-life	17.07.2023	31.10.2022	EU, 2022	[48]
10	Clesrovimab (MK-1654)	Enflonsia	Respiratory syncytial virus F glycoprotein; Viral fusion protein inhibitor	Prevention of respiratory syncytial virus infection	Merck Sharp & Dohme; US	Merck Sharp & Dohme; US	Human IgG1 kappa; M252Y/S254T/T256E (YTE); Extends half-life	09.06.2025	NA	US, 2025	[35]
11	Vilobelimab (CaCP29; IFX-1)	Gohibic	C5a receptor on human immune cells; Complement C5a inhibitor	Treatment of COVID-19-induced ARDS	InflaRx; Germany/US	InflaRx/Staidson Beijing BioPharmaceuticals; Germany/US/China	Humanized IgG4 kappa; None	04.04.2023 (EUA)	13.01.2025	US, 2023	[73]
12	Pemivibart (VYD222)	Pemgarda	SARS-CoV-2 Spike protein; Virus internalisation inhibitor	Prevention of COVID-19	Invivyd; US	Invivyd; US	Human IgG1 kappa; M435L/N441A (LA); Extends half-life	22.03.2024 (EUA)	NA	US, 2024	[74]
13	Sipavibart (AZD3152, Omi-42)	Kavigale	SARS-CoV-2 Spike protein; Virus internalisation inhibitor	Prevention of COVID-19	RQ Bio; UK	RQ Bio/AstraZeneca; UK	Human IgG1 lambda; L234F/L235E/P331S (TM); Reduce effector function; M252Y/S254T/T256E (YTE); Extends half-life	NA	20.01.2025	EU, 2025	[75]

**Table 2:** Recombinant antibody-based antivirals in late-stage clinical trials.

#	Name (Alternative Names)	AdisInsight ID	Target; Mechanism of Action	Active Indications (Highest Phase)	Latest Study Start	Latest Study Status	Originator; Country	Sponsor; Country	ClinicalTrials.gov ID (Chronological)	Reference
1	Semzuvolimab (UB-421; B4C7)	800014102	CD4 receptor on human T-cells; HIV fusion inhibitor	Phase III	01.03.2024	Not yet recruiting	United Biomedical; US	United BioPharma/UBP Greater China; Taiwan/China	NCT04406727 (Phase III); NCT04620291 (Phase I); NCT04985890 (Phase II)	[76]
2	Teropavimab/Zinlirvimab (3BNC117-LS/10-1074-LS; GS-5423/GS-2872)	800053320/800057114	HIV-1 envelope protein gp120 (CD4 binding site/V3 loop); Virus internalisation inhibitors	Phase II	05.09.2024	Recruiting	Rockefeller University; US	Gilead Sciences/NIAID; US	NCT04319367 (Phase II); NCT05245292 (Phase I); NCT05729568 (Phase II); NCT05612178 (Phase I); NCT06071767 (Phase I/II); NCT05300035 (Phase II); NCT06031272 (Phase I); NCT07054931 (Phase II)	[77,78]
3	N6LS (VH3810109; GSK3810109)	800056714	HIV-1 envelope protein gp120; Virus internalisation inhibitor	Phase II	10.07.2025	Recruiting	NIAID; US	ViiV Healthcare; UK	NCT05996471 (Phase II); NCT07053384 (Phase I)	[79]
4	VRC07-523LS (TMB-380) in mAb combinations (with CAP256V2LS; TMB-365; PGT121.414.LS; PGDM1400LS)	800057129 (800067844/800057180/800050846)	HIV-1 envelope protein gp120; Virus internalisation inhibitors	Phase II	15.02.2026	Not yet recruiting	NIAID (NIAID/ADARC/Theraclone Science/NIAID); US	NIAID/TaiMed Biologics; US/Taiwan	NCT04983030 (Phase I/II); NCT05890963 (Phase I); NCT06812494 (Phase II); NCT07215468 (Phase II); NCT06517693 (Phase I); NCT06987318 (Phase I)	[80,81,82,83,84]
5	Tobevibart (BRII-877; VIR-3434)	800056267	HBsAg; Virus internalisation inhibitor (hepatitis B virus/hepatitis D virus)	Phase II/III	05.08.2025	Recruiting	Vir Biotechnology; US	Vir Biotechnology; US	NCT05484206 (Phase II); NCT06216470 (Phase II); NCT06903338 (Phase III); NCT07128550 (Phase III); NCT07142811 (Phase II)	[85]
6	Silevimig (GR1801)	800073709	Rabies virus glycoprotein (G protein); Virus internalisation inhibitor	Phase III	21.10.2022	Completed (06.09.2024)	Genrix (Shanghai) Biopharmaceutical; China	Genrix (Shanghai) Biopharmaceutical; China	NCT05846568 (Phase III)	[86]
7	AV-1	800057569	Dengue virus envelope protein; Viral fusion protein inhibitor	Phase II	07.01.2025	Active, not recruiting	AbViro; US	AbViro; US	NCT06799741 (Phase II)	NA
8	TNM001	800071192	Respiratory syncytial virus F glycoprotein; Viral fusion protein inhibitor	Phase III	30.11.2024	Not yet recruiting	Trinomab Biotech; China	Zhuhai Trinomab Pharmaceutical; China	NCT06083623 (Phase II/Phase III); NCT06710925 (Phase III)	NA
9	AK0610	800066806	Respiratory syncytial virus F glycoprotein; Viral fusion protein inhibitor	Phase II	30.10.2025	Recruiting	Ark Biosciences; China	Shanghai Ark Biopharmaceutical; China	NCT06996704 (Phase I/II)	NA
10	AER002 (P2G3)	800073917	SARS-CoV-2 Spike protein; Virus internalisation inhibitor	Phase II	01.08.2023	Active, not recruiting	Aerium Therapeutics; US	Michael Peluso, MD; US	NCT05877508 (Phase II)	[87]
11	SA55 (BD55-5514)	800075048	SARS-CoV-2 Spike protein; Virus internalisation inhibitor	Phase II	31.08.2023	Recruiting	Sinovac Biotech; China	Sinovac Life Sciences; China	NCT06042764 (Phase II)	[88]
12	VYD2311	800077852	SARS-CoV-2 Spike protein; Virus internalisation inhibitor	Phase III	08.01.2026	Recruiting	Invivyd; US	Invivyd; US	NCT06523153 (Phase I); NCT07298434 (Phase III)	[89]

## 4. mRNA-Based Platforms for Antibody Therapeutics

The application of targeted exogenous mRNA delivery for transient in vivo protein expression has become widespread over the past decade [90,91]. Today, mRNA-based therapeutics are considered one of the most promising tools for the prevention and treatment of a broad range of diseases, including viral infections [92,93,94,95,96,97,98,99].

Exogenous mRNA sequences are obtained by in vitro transcription. This typically involves a DNA-dependent RNA polymerase, usually from the T7 phage, and a linear plasmid that serves as the DNA template. The plasmid contains the T7 promoter and the protein-coding sequence flanked by 5′- and 3′-untranslated regions (UTRs) [100,101]. Translation efficiency and stability of exogenous mRNA are ensured by the 5′-end cap [102] and the 3′-end poly-A tail [103,104,105], which can be incorporated during either co-transcription or individual enzymatic reactions [106,107]. Although modern technology makes mRNA synthesis relatively simple and fast, specific synthesis conditions can substantially influence the amount of translated protein, the reactogenicity of mRNA in vivo, and its therapeutic potential [108,109,110].

Thus, when designing mRNA (Figure 1C), much attention is paid to the UTR sequences [111]. The UTRs of mRNA molecules form secondary structures that are essential for proper ribosome scanning and dissociation, and they affect both the translation rate and the mRNA half-life in cells [112]. In native human mRNAs, the 5′-UTR typically ranges from 53 to 218 nucleotides, whereas the 3′-UTR is highly heterogeneous, varying from hundreds to thousands of nucleotides, with an average length of roughly 1000 [113]. When designing exogenous mRNA, the UTRs are shortened to reduce the risk of generating aberrant transcripts and unintended protein products [114].

Another equally important factor that can affect mRNA translation efficiency and potentially cause serious side effects is mRNA reactogenicity [115,116]. Exogenous mRNAs are recognized by the innate immune system via the pattern recognition receptors (PRRs), particularly Toll-like receptors (TLR3, TLR7, and TLR8) and RIG-I-like receptors [117,118]. This triggers an immune response that can suppress protein translation and cause undesirable side effects. One efficient approach to reducing mRNA reactogenicity is the inclusion of modified natural nucleotides to decrease mRNA affinity towards PRRs and prevent immune activation [118,119,120,121,122,123,124]. It is chemical nucleotide modifications that underpinned the progress in mRNA technology [125], and Katalin Karikó and Drew Weissman were awarded the 2023 Nobel Prize in Physiology or Medicine for their discoveries concerning nucleoside base modifications that enabled the development of effective mRNA vaccines against COVID-19 [126,127].

The final step in exogenous mRNA production is purification. Byproducts generated during abortive reactions, such as double-stranded RNA impurities and DNA fragments, can also activate TLRs and trigger immunostimulatory responses [128]. Purification of mRNA with modified bases using high-performance liquid chromatography or cellulose chromatography with an ethanol-containing buffer has been shown to remove up to 90% of these contaminants, regardless of mRNA length, coding sequence structure, or nucleotide composition, thereby reducing reactogenicity [129,130].

To summarize, the therapeutic potential of exogenous mRNAs that encode antibodies is based on three pillars of synthetic technology: (1) plasmid engineering for in vitro synthesis, including the intracellular kinetic regulators (UTR) and the consensus protein translation initiation site, such as the Kozak sequence; (2) an optimized synthesis protocol, incorporating modified nucleotides, the 5′-cap or its highly efficient synthetic analogs, and a high-performance enzyme (e.g., mutant T7 polymerase); (3) optimized high-performance liquid chromatography purification of exogenous mRNA. It is important to note that, while some immunogenicity is acceptable and potentially beneficial for mRNA vaccines, it is highly undesirable in mRNA-based drugs, as it directly limits their therapeutic potential.

mRNA therapeutics are considered effective if they achieve the desired concentration of the encoded antibody in target cells, the bloodstream, or specific organs or tissues [131]. In principle, exogenous mRNA must cross the lipid bilayer to enter target cells and be translated into functional protein [63]. Various cell types can spontaneously take up mRNA through different mechanisms, although this often results in mRNA being captured in acidic endolysosomal compartments, leading to its degradation [132]. Consequently, the safety and efficacy of mRNA-based therapy can be improved by developing delivery systems that enable targeted and secure transport [133,134,135,136,137]. Delivery systems generally serve to protect mRNA from degradation and facilitate its cellular entry and efficient translation (Figure 1D).

To date, lipid nanoparticles (LNPs) remain the most widely used tool for the delivery of exogenous mRNA [138,139,140,141]. All LNPs approved for clinical use are composed of four lipid types: an ionizable cationic lipid, cholesterol, an auxiliary phospholipid, and a lipid conjugated with polyethylene glycol (PEG) [142,143,144,145]. Such a composition creates a monodisperse LNP system, which can efficiently encapsulate exogenous mRNA and deliver it into cells [146]. The lipid component ratio, and particularly the proportion of the auxiliary phospholipid, can be adjusted to modify the LNPs’ charge, which facilitates targeted delivery of mRNA to specific organs, primarily the spleen and lungs. A variety of lipids are currently commercially available, and methods for formulating LNP compositions that ensure selective action of therapeutic mRNA in target organs and tissues have been described [147,148,149,150,151,152,153,154]. In addition, novel approaches for mRNA delivery are being developed in which LNPs are functionalized with single-domain antibodies that interact with receptors of target cells [155,156,157]. However, despite these advances, targeted in vivo delivery of therapeutic mRNA to specific cell populations remains challenging [158].

Most mRNA–LNP formulations contain lipids conjugated to PEG. In clinical studies, mRNA–LNPs have been shown to induce anti-PEG antibodies that may compromise therapeutic efficacy upon repeated administration [159,160]. Importantly, pre-existing anti-PEG antibodies do not appear to interfere with the primary immune response to mRNA therapeutics; however, antibody titres increase with repeated dosing, which raises concerns for long-term treatment regimens [161]. One strategy to mitigate this effect is optimization of the route of administration, as intramuscular injection elicits a weaker anti-PEG response than subcutaneous delivery [162]. In parallel, research efforts are focused on the development of alternative polymeric lipids with reduced immunogenicity. For example, safer repeated administration can be achieved by substituting PEG with poly(2-oxazoline) [163] or using brush-shaped polymeric lipids [164], which creates a steric shield that limits antibody binding.

Much of the current research focuses on the development of LNPs for intranasal (i.n.,) and pulmonary mRNA delivery, which enables mRNA to cross epithelial barriers in the respiratory tract. The main goal is to optimize LNP composition since stability of LNPs during aerosolization depends on the PEG-lipid content and the nature of the auxiliary lipid. These parameters also influence how effectively LNPs move through the mucosal layer, ultimately affecting the efficiency of mRNA transfection and target protein expression [165,166,167,168,169,170,171,172].

For in vivo applications, several routes of administering mRNA–LNP complexes are being explored, including intramuscular (i.m.,), intradermal, subcutaneous, intravenous (i.v.,), and intratracheal (i.t.,) delivery, as well as spinal injections and delivery to regional lymph nodes [100]. For therapeutic exogenous mRNA encoding antibodies against respiratory viruses, local delivery to the lungs via aerosol spray [173,174,175,176], e.g., unformulated mRNA in nuclease-free water, is considered the most suitable approach [177,178,179,180].

Unlike recombinant antibodies, which are usually given as a single—and typically the highest tolerable—dose, exogenous mRNA can support sustained antibody production for multiple days. mRNA half-life varies substantially depending on system design, including the UTRs used and the mode of mRNA delivery, particularly the structure of LNPs [181,182]. At later stages, antibody levels are mostly determined by the pharmacokinetics of the protein itself, which depends on its structure. For instance, fragments and truncated forms of antibodies are known to have a shorter half-life in the bloodstream than full-length IgG because they lack the Fc domain. The interaction between the IgG Fc region and the neonatal Fc receptor, located in endothelial cells, intestinal epithelium, and antigen-presenting cells, protects IgG from lysosomal degradation during endocytosis and promotes its transport to the cell surface, where it is released into the alkaline environment of the bloodstream [183]. Still, truncated mRNA-encoded immunoglobulins can reach and maintain therapeutic levels without frequent repeat dosing [184].

Thus, exogenous mRNAs that encode antibodies offer several advantages over protein-based antibody therapy: (1) rapidly advancing technologies help lower production costs and standardize manufacturing of mRNA therapeutics; (2) design and development of new antibody-encoding mRNAs can be completed more quickly using established technological platforms; (3) mRNA technology allow creating new antibody formats, such as intracellular or membrane-anchored variants, although this will require further progress in delivery systems; (4) this approach reduces contamination of exogenous mRNA preparations with components of animal or cell origin; (5) purification protocols are universal across mRNAs with different coding sequences and, therefore, require minimal optimization; (6) high translation rates and elevated local antibody concentration in situ can lower the dose needed to achieve a therapeutic effect.

This review primarily focuses on the development of mRNA-encoded therapeutic antibodies for passive immunization and the assessment of their antiviral efficacy in vivo. However, the success of mRNA vaccine technology has enabled an alternative strategy that is gaining increasing attention: inducing neutralizing antibodies by delivering mRNA that encodes a target viral protein—an approach known as mRNA immunization.

This antigen-encoding mRNA–LNP platform has proven highly versatile and efficient for the rapid generation of high-affinity, neutralizing monoclonal antibodies against a wide range of pathogens and challenging biological targets [185,186,187,188]. Moreover, it can be integrated with classical technologies, such as hybridoma [185,187,188,189], as well as with modern high-throughput approaches, including single B cell receptor sequencing (scBCR-seq) [186,190]. mRNA immunization overcomes several limitations associated with recombinant protein–based immunization, including antigen instability, the absence of native post-translational modifications (e.g., glycosylation), and difficulties in the expression and purification of transmembrane proteins [186,188,191].

Studies on SARS-CoV-2 illustrate the strengths of this approach: mRNA immunization not only enables the rapid generation of high-affinity antibodies with broad neutralizing activity against multiple viral variants [187,192], but also reduces overall development timelines by nearly twofold [185]. Beyond SARS-CoV-2, the platform has been successfully applied to the generation of antibodies against HIV-1 [191], mpox virus (formerly monkeypox) [190], and feline herpesvirus [189]. The last two examples also suggest potential diagnostic applications due to the rapid production of highly sensitive antibodies. Moreover, mRNA immunization has proven effective for generating antibodies against structurally complex transmembrane therapeutic targets, including GPCR (PAR4) [188], CD22, and GPRC5D [186], while preserving their native conformations and conformational epitopes.

Together, these findings establish mRNA–LNP–based immunization as a promising, versatile, and highly adaptable platform that allows for a faster development of therapeutic and diagnostic antibodies. This is particularly relevant for emerging and re-emerging infectious diseases, as well as for structurally complex transmembrane targets that remain difficult to address using conventional technologies.

## 5. Rational Design of Antibody-Encoding mRNAs

**Figure 2:**
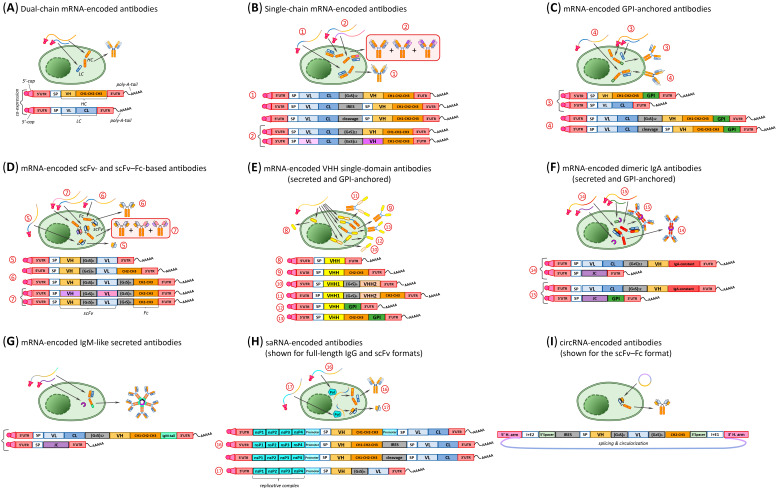
Design strategies for antibody-encoding mRNA constructs. (**A**) A dual HC/LC mRNA system contains two separate mRNAs encoding heavy (HC, orange) and light (LC, blue) chains of immunoglobulin G. A functional antibody is formed when both chains are co-expressed in a cell. (**B**) SC-mRNA format is a single mRNA sequence that encodes both antibody chains. HC and LC can be translated as a single polypeptide that subsequently folds into a mature antibody (in the presence of a glycine–serine linker), translated independently (using an IRES), or expressed as a single polypeptide with subsequent proteolytic cleavage (via a cleavage site). Co-expression of two SC-mRNAs encoding different full-length antibodies generates three functional products: two parental monospecific antibodies and one bispecific antibody. (**C**) A construct encoding membrane-anchored antibodies with a GPI anchor at the C-terminus of the heavy chain (HC). (**D**) Schematic design principles of mRNAs for generating scFv and scFv–Fc (Fc domain shown in orange). The Fc domain can be fused to the VH domain either directly or via a glycine–serine linker. Co-expression of two mRNAs encoding distinct scFv–Fc constructs produces three chimeric antibody species: two monospecific homodimers and one bispecific heterodimer. (**E**) mRNA sequences encoding VHH single-domain antibodies (yellow) in various formats: monomeric, anchored, fused VHH–Fc, and anchored VHH–Fc (with an Fc domain shown in orange). (**F**) Assembly of IgA antibody dimers (dIgA) is achieved by co-expression of mRNAs encoding HC, LC, and the joining chain (JC) required for dimerization. The addition of GPI anchor at the C-terminus of JC anchors dIgA in the membrane. (**G**) Construct encoding pentameric IgM-like antibodies. Multimerization is achieved through co-expression of SC-mRNA containing an IgM tailpiece sequence (aquamarine) fused to the IgG-CH domain and JC. (**H**) saRNA design: after translation, the replicative complex (nsP1–4) amplifies the antibody genes controlled by one or two subgenomic promoters. (**I**) circRNA encoding an antibody in the scFv–Fc format. All schematics depict a cell (light green) with a nucleus (dark green). The main structural elements of the RNA constructs are: 5′- and 3′-UTR (pink)—5′- and 3′-untranslated regions; SP (white)—signal peptide for antibody secretion; VH (orange), VL (blue)—variable domains of HC and LC; CH1-CH2-CH3 (dark orange)—constant domains of HC; CL (dark blue)—constant domain of LC; (G_4_S)_12_ (grey)—glycine–serine linker; IRES (grey)—internal ribosome entry site; cleavage (grey)—proteolytic cleavage site; GPI (green)—GPI anchor sequence; VHH (yellow)—single-domain antibody; JC (violet)—joining chain sequence; IgA-constant (red)—constant region of IgA; IgM-tail (aquamarine)—tailpiece sequence (PTLYNVSLVMSDTAGTCY); nsP1, nsP2, nsP3, nsP4 (cyan)—alphavirus nonstructural proteins that assemble into an saRNA replication complex; Promoter (light blue)—subgenomic promoter; 5′- and 3′-H. arm (pink)—5′- and 3′-homology arms; I+E—exon and intron. Circled numbers indicate individual mRNA constructs and their corresponding expressed antibody products.

Like protein-based therapeutics, mRNA-encoded antibodies can adopt various structures but are translated in vivo. Various approaches to mRNA design have been developed to ensure efficient expression of antibodies targeting viral pathogens (Figure 2).

The most direct extension of recombinant antibody technology is the dual HC/LC mRNA system (Figure 2A), which enables co-expression of mRNAs encoding both HC and LC of an immunoglobulin. The two mRNAs can be formulated at a defined ratio, most commonly 2:1 (HC:LC), and encapsulated either within a single LNP or in separate LNPs. This design is used in most studies discussed in this review. Its limitations include the risk of imbalanced HC and LC expression, potentially resulting in non-functional antibody molecules, as well as the added complexity of combination therapies involving antibody cocktails. A case in point is a 2025 study from the Santangelo group [180], which showed that partial or complete heavy-light mismatched antibodies can form when four mRNAs encoding the chains of two distinct antibodies are delivered simultaneously.

To optimize the HC and LC assembly, a single-chain mRNA (SC-mRNA) strategy has been suggested (Figure 2B). In this configuration, a single transcript encodes both chains of a full-length antibody. The two chains can be separated by a flexible linker [193] that permits heterodimer formation from a single polypeptide, or by an internal ribosome entry site (IRES) enabling independent translation, or by a proteolytic cleavage site such as P2A or T2A [194]. The SC-mRNA format also offers practical advantages for combination antibody therapies. Co-expression of two SC-mRNAs encoding distinct antibodies results in the formation of three functional recombinant antibody species with correctly paired HC and LC: two monospecific antibodies and one bispecific antibody [195,196,197,198]. In this context, SC-mRNA offers a decisive advantage over dual HC/LC mRNA systems that can produce non-functional antibodies due to partial or complete HC–LC mismatching.

mRNA technology enables therapeutic application of rare antibody formats that are difficult or impractical to produce as proteins. For example, mRNA-encoded antibodies targeting HIV-1 [178,180], RSV [177], or SARS-CoV-2 [199] have been engineered to remain anchored in the membranes of producer cells (Figure 2C). Such constructs can be implemented using either the dual HC/LC mRNA system or SC-mRNA. In these designs, the C terminus of the HC is fused to a glycosylphosphatidylinositol (GPI) anchor derived from decay-accelerating factor (DAF) [200], which retains the antibody at the cell surface after export. Compared to systemic antibody delivery, membrane anchoring of neutralizing antibodies has several advantages: (1) it limits antibody diffusion away from infected tissues, (2) increases the local concentration of antibody in the target organ, and (3) may prolong antibody residence at mucosal surfaces [177].

In addition to full-length immunoglobulins, mRNA platforms enable the in situ production of diverse antibody formats. These include truncated designs, such as scFv (Figure 2D) [201], as well as monomeric or oligomeric VHH single-domain antibodies (Figure 2E) [202,203,204,205,206]. To enhance their therapeutic properties, these formats can be fused to an Fc domain or attached to the membrane via a GPI anchor. Co-expression of two mRNAs encoding different scFvs fused to a common Fc domain (Figure 2D) yields two homodimeric and one heterodimeric chimeric antibody species with correct pairing, enabling effective combination therapy [207]. In addition, mRNA constructs encoding intrinsically bispecific antibodies, such as RiboBiFEs [208], have been developed. These molecules incorporate two VHH single-domain antibodies connected by a linker (Figure 2E), with each VHH capable of selectively binding a distinct target, which confers high specificity to the immune response.

Literature describes mRNAs encoding IgA dimers (dIgA) that demonstrate increased avidity and enhanced protective activity at mucosal surfaces [180,209,210,211]. This antibody format remains effective when expressed in a membrane-anchored configuration (Figure 2F). A similar mRNA design can be extended to secreted antibodies assembled as IgM-like pentamers (Figure 2G) [180].

In addition to conventional mRNA constructs, antibodies encoded by self-amplifying RNA (saRNA) [212,213] have shown strong protective efficacy in animal models of Zika virus infection [214] and SARS-CoV-2 [215,216]. saRNA constructs encode the alphavirus replicative machinery, which drives intracellular amplification of HC- and LC-encoding sequences regulated by one or two subgenomic promoters (Figure 2H). This design enables more sustained and efficient antibody expression. The HC and LC can also be produced using two separate saRNAs. Furthermore, saRNA technology is applicable for expressing antibodies in the scFv format.

Circular RNAs (circRNAs) are being actively explored as an alternative to linear mRNAs for therapeutic antibody expression [217,218,219,220]. Figure 2I illustrates the structure of a circRNA encoding an scFv–Fc antibody targeting SARS-CoV-2 [174]. Due to their covalently closed architecture, which lacks free 5′- and 3′-ends, circRNAs are more resistant to exonuclease-mediated degradation. This helps substantially increase their intracellular stability: reported in vitro half-lives reach up to 90 h, compared with 15–24 h for linear mRNAs. The increased stability supports more prolonged and intensive expression of the encoded protein, an advantage for therapeutic applications requiring sustained expression. Furthermore, the circular structure may help reduce immunogenicity, although this remains debated. While some studies report activation of RIG-I signaling, others observe minimal innate immune responses, particularly with highly purified or chemically modified circRNAs. Overall, circRNAs represent a promising platform for therapeutic antibody expression, combining enhanced stability, prolonged expression, and a potentially improved safety profile.

Moreover, exogenous mRNA technology enables the use of therapeutic intracellular antibodies (intrabodies), which are difficult to deliver into cells as mature proteins [221,222,223,224]. These antibodies do not exit the cell after maturation, binding target antigens in the cytoplasm or other intracellular compartments, whereas conventional circulating therapeutic antibodies interact with serum or membrane antigens [225,226,227,228]. In comparison, proteins translated from exogenous mRNA can function as highly specific intracellular inhibitors with high affinity [229] and can be engineered to target non-structural viral proteins, such as the polymerase complex [230]. It should be noted that, despite growing interest in this approach, no animal studies evaluating the efficacy of mRNAs encoding antiviral intrabodies have been reported as of January 2026. To date, only two studies have reported successful in vivo delivery of intrabodies using the mRNA–LNP platform. In a murine model, mRNA-encoded intracellular VHH single-domain antibodies specifically inhibited the bacterial effector proteins Etf-2 [231] and Etf-3 [232] of *Ehrlichia chaffeensis*, disrupting its survival in infected cells.

## 6. mRNA-Encoded Recombinant Antibodies in Antiviral Therapy

### 6.1. mRNA-Encoded Recombinant Antibodies

Since 2021, over a thousand peer-reviewed articles on mRNA design and its clinical applications as vaccines and therapeutic agents have been published each year. This section highlights key examples of the development and in vivo evaluation of exogenous mRNAs encoding various antibodies against viral pathogens [233,234,235,236]. Table 3 summarizes mRNA-based therapeutics developed since 2017 for in vivo production of recombinant antibodies targeting viral pathogens.

The introduction of hybridoma technology for monoclonal antibody production in 1975 by Jerne, Köhler, and Milstein [237], later recognized with the 1984 Nobel Prize in Physiology or Medicine, catalyzed the use of specific antibodies, both monoclonal and polyclonal, as therapeutic agents. Early studies were focused on the efficiency of monoclonal antibodies in leukemia treatment [238,239,240,241,242].

One of the earliest well-known studies on the activity of mRNA-encoded antibodies was published in *Cell* by a German research group in 1984 [243]. The work evaluated the antiviral activity of exogenous mRNAs that encoded antibodies against Golgi α-mannosidase II (GMII). Total mRNA was isolated from mouse hybridoma cells producing the 53FC3 antibodies against GMII [244] and then introduced into the cytoplasm of eukaryotic BHK-21 cells by direct microneedle injection. Within 5–6 h, mouse immunoglobulins accumulated in the Golgi complex, inhibiting the intracellular transport of viral membrane proteins. After infection with vesicular stomatitis virus, cells containing the 53FC3 mRNA showed significantly lower expression of the viral G protein on their surface compared to control cells [244].

In 1990, Wolff et al. [245] demonstrated that, in mice, exogenous mRNA can be successfully translated into proteins in muscle cells by cellular enzymes in situ. Despite this promising discovery, clinical application of in vitro-produced mRNA has long been limited by its instability, susceptibility to ribonuclease degradation, immunogenicity, and the complexity of developing suitable delivery systems. The concept of employing exogenous mRNA to encode therapeutic antibodies rather than directly administering immunoglobulins was officially introduced in 2008 by the German company *CureVac* in its patent application ‘RNA-coded antibody’ [246].

Beyond viral infections, mRNA-encoded antibodies also represent a promising therapeutic modality for a broad spectrum of bacterial diseases. For example, mRNAs encoding monoclonal antibodies against the VapA protein of *Rhodococcus equi* have been developed for inhalational treatment of pneumonia [172]. Both full-length antibodies [247] and scFv-fragments [248] targeting the type III secretion system have been generated to combat infections caused by multidrug-resistant *Pseudomonas aeruginosa*. Against botulinum neurotoxins serotypes A, B, and E produced by *Clostridium botulinum*, chimeric antibodies based on VHH single-domain antibodies fused to the human IgG1 Fc region (VHH–Fc) have been developed [249,250], and multimeric antibody formats providing broad neutralization have also been reported [251]. To address intracellular bacterial infections such as human monocytic ehrlichiosis (*Ehrlichia chaffeensis*), mRNA-encoded intracellular VHH single-domain antibodies targeting the effectors Etf-2 [231] and Etf-3 [232] are under active investigation. Other applications include mRNA-encoded VHH-based antibodies against toxins A and B of *Clostridioides difficile* [252]; passive immunization through breast milk expressing IgG ZAC-3 against *Vibrio cholerae* [253]; and mucosal expression of IgA-class antibodies in the intestinal and respiratory tracts targeting *Salmonella enterica* and *Pseudomonas aeruginosa* [211].

**Table 3:** mRNA-based therapeutics for in vivo expression of neutralizing antibodies against viral pathogens.

Virus Species	Study Number in Review	Original Antibody (Reference)	mRNA Therapeutic	Viral Target	Antibody Type	Antibody Expression Method	Delivery Method, Vehicle Composition, Route of Administration, Dosing Regimen, Time Before and/or After Infection	Animal Species and Study Design	Reference (Originator)
HIV-1	1	VRC01 [254]	VRC01 mRNA-LNP	Surface Env-gp120 glycoprotein (CD4 binding site)	Full-length IgG antibody	Co-expression of mRNAs encoding HC and LC	LNPs (ionizable cationic lipid/DSPC/cholesterol/PEG-lipid at molar ratios of 50:10:38.5:1.5), i.v., up to 1.4 mg/kg, 24 h prior to infection	BALB/c, C57BL/6, and NSG mice (pharmacokinetic study); humanized CD34-NSG and BLT mice (protective efficacy was demonstrated under a prophylactic mRNA administration regimen in a viral infection model using the primary isolates SF162 and JR-CSF)	[255] (*Acuitas Therapeutics*)
	2	PGT121 [83]	aPGT121 mRNA, sPGT121 mRNA	Surface Env-gp120 glycoprotein	Full-length IgG antibody: secreted (sPGT121) and GPI-anchored via the heavy chain (aPGT121)	Co-expression of mRNAs encoding HC and LC (for the assembly of both secreted IgG and the GPI-anchored forms)	Unformulated mRNA in nuclease-free water, delivered intravaginally as an aerosol using the *MADgic Teleflex* aerosolizer, applied to the cervical mucosa, up to 1 mg of mRNA per animal	Katahdin sheep, rhesus macaques (pharmacokinetic study)	[178]
	3	PGDM1400 [84], PGT121 [83], N6 [79]	PGDM1400 scFv–Fc, PGT121 scFv–Fc, N6 IgG	Surface glycoprotein Env: gp140 (PGDM1400), gp120 (PGT121), CD4 binding site in gp120 (N6)	A cocktail containing two scFv–Fc and one full-length IgG antibody	Co-expression of four mRNAs encoding three antibodies: two mRNA encoding two scFv–Fc antibodies (single-chain constructs) and two mRNA encoding IgG HC and LC	LNPs (ionizable lipid/DSPC/cholesterol/PEG-lipid at molar ratios of 50:10:38.5:1.5), i.v., 1 mg/kg	Hemizygous Tg32 mice (hFcRn; pharmacokinetic study)	[207] (*Moderna*)
	4	PGT121 [83], VRC07 [80], 10E8.4 [256], iMab [257], CAP256 [258], PGDM1400 [84], J3-VHH [259]	Multiple strategies and antibodies tested in animal models, including mRNA SC-PGT121 aIgG, SC-VRC07 aIgG; SC-PGT121 aIgA, SC-VRC07 aIgA; SC-PGT121 sIgA, SC-VRC07 sIgA; SC-PGT121 IgM-tail, SC-VRC07 IgM-tail; combinations of SC-PGT121 and SC-VRC07 aIgG, aIgA, sIgA and IgM-tail; aJ3-2×-Fc	Surface glycoprotein Env: gp120 V3 loop (PGT121), CD4 binding site of gp120 (VRC07, J3-VHH)	GPI-anchored IgG (aIgG), secreted IgA dimer (sIgA), GPI-anchored IgA dimer (aIgA), IgM-like multimer by incorporating an IgM tailpiece sequence at the end of IgG-CH (IgM-tail); GPI-anchored VHH–Fc (aVHH–Fc)	In rhesus macaques: single-chain version of the GPI-anchored IgG (SC-aIgG): LC and HC mRNAs merged into a single mRNA strand using (G_4_S)_12_ linker, with a GPI anchor at the C-end of HC; SC-sIgA and SC-aIgA: co-expression of mRNA-encoded IgA (LC-linker-HC) and mRNA-encoded JC (a secreted JC (sJC) for secreted IgA (SC-sIgA) and a GPI-anchored JC (aJC) for membrane-bound IgA (SC-aIgA)), at a 1:0.15 ratio; IgM-tail: co-expression of mRNA encoding single-chain IgG fused to a C-terminal IgM tailpiece and mRNA encoding the JC (sJC) at a ratio of 1:0.15; for co-expression of two different antibodies, simultaneous delivery of two separate single-chain aIgG mRNAs at a 1:1 ratio, or, in the case of IgA/IgM-tail formats, additional co-expression of JC mRNA at a ratio of 1:1:0.3; aVHH-2×-Fc: expression of mRNA encoding two repeated VHH domains linked via a (G_4_S)_12_ linker, with addition of an IgG heavy-chain constant fragment (Fc) and a C-terminal GPI anchor. In mice: co-expression of mRNA-encoded antibodies in sIgA and IgM-tail formats together with the JC at a ratio of 4:4:2.	In rhesus macaques: unformulated mRNA in nuclease-free water, delivered intravaginally as an aerosol using the *MADgic Teleflex* aerosolizer, applied to the cervical mucosa, up to 1 mg of mRNA per animal in a total volume of 300 µL (two 150-µL doses administered to different areas); biopsy samples for ex vivo analyses were collected 24 h after administration. In mice: LNPs (cKK-E12/DOPE/cholesterol/C14-PEG 2000-P at molar ratios of 35:16:46.5:2.5); the mRNAs were diluted in 10 mM citrate buffer (pH 3); intravenous, retro-orbital injection, 2 mg/kg, given twice with a 2-day interval; antibody concentrations in serum were measured on day 3 after administration.	Rhesus macaques (ex vivo infection of vaginal explants; preclinical studies of safety, pharmacokinetics, and toxicity; hematological analyses, immunophenotyping, histological evaluation); C57BL/6 mice (pharmacokinetics, serum neutralization assay); SHIV/HIV strains, including isolates from clades A, B, C и AE	[180]
	5	ePGDM1400v9 (optimized version PGDM1400, [84])	mRNA-ePGDM1400v9 IgG1, mRNA-ePGDM1400v9 IgA2	Surface Env glycoprotein: gp140	Full-length IgG1 antibody containing an LS mutation, full-length IgA2 antibody dimer	Co-expression of mRNAs encoding IgG1 HC and LC; co-expression of mRNAs encoding HC, LC, and the IgA2 JC	LNPs (ionizable lipid/DSPC/cholesterol/PEG-lipid at molar ratios of 50:10:38.5:1.5), i.v., 1 mg/kg	Rhesus macaques (preclinical studies of safety, pharmacokinetics, and biodistribution; evaluation of activity using an HIV-based pseudovirus neutralization assay)	[209] (*Moderna*)
Hepatitis B virus	6	G12 [260]	mL (G12-scFv), mL (G12-scFv–Fc), mL (G12-IgG)	Surface antigen S protein (HBsAg)	Chimeric scFv–Fc antibody and full-length IgG antibody	Expression of mRNA encoding scFv; expression of mRNA encoding scFv–Fc; co-expression of mRNAs encoding HC and LC	LNPs (SM-102/DSPC/cholesterol/DMG-PEG2k at molar ratios of 50:10:38.5:1.5), i.v., 2.5 mg/kg, 2 weeks post-infection	C57BL/6 mice (protective efficacy was demonstrated under a therapeutic mRNA administration regimen in a chronic hepatitis B infection model using an adeno-associated virus)	[261]
Ebola virus	7	2G1 [262]	mRNA-2G1-LNP	GP glycoprotein	Full-length IgG antibody	Co-expression of mRNAs encoding HC and LC	LNPs (SM-102/DSPC/cholesterol/PEG-lipid at molar ratios of 50:10:38.5:1.5), i.v., 1 mg/kg, 12 h prior to infection	BALB/c mice (protective efficacy was demonstrated under a therapeutic mRNA administration regimen in an HIV-based Ebola pseudovirus infection model)	[263]
Orthopoxviruses: vaccinia virus (VACV), mpox virus (MPXV), variola virus (VARV)	8	c7D11 [264], c8A [265], and c6C [266]	c7D11 mRNA, c8A mRNA, and c6C mRNA	VACV-L1 protein of the intracellular mature virion (IMV) (c7D11), VACV-B5 glycoprotein of the extracellular enveloped virion (EEV) (c8A), and VACV-A33 glycoprotein of the EEV (c6C)	Three chimeric full-length IgG antibodies containing human constant regions and variable regions derived from mouse (c7D11) and monkey (c8A and c6C)	Co-expression of mRNAs encoding HC and LC from the c7D11, c8A, and c6C antibodies; expressed both individually and in combinations, administered as separate injections; each antibody was encoded by its own pair of mRNAs (HC and LC), with each pair individually encapsulated in LNPs; mRNAs contained no modified nucleotides	LNPs (proprietary, *Arcturus Therapeutics*), i.m., up to 0.9 mg/kg	New Zealand White rabbits (pharmacokinetic study)	[267] (*CureVac*)
	9	mAb22, mAb26 ([268], S1, Table 2)	Mix2a: mRNA-mab22-LNP + mRNA-mAb26-LNP	VACV-A33 EEV glycoprotein (mAb22) and MPXV-M1 IMV (mAb26) protein	A cocktail of two full-length IgG antibodies	Co-expression of mRNAs encoding HC and LC from two antibodies	LNPs (ionizable lipid/DSPC/cholesterol/PEG-lipid at molar ratios of 50:10:38.5:1.5), i.v., 1 mg/kg, 24 h prior to infection	BALB/c mice (protective efficacy was demonstrated under a prophylactic mRNA administration regimen in a lethal VACV-induced infection model)	[268]
Rabies virus	10	CR57 [269]	mRNA-LNP encoding anti-rabies mAb	G glycoprotein	Full-length IgG antibody	Co-expression of mRNAs encoding HC and LC; the mRNA did not contain modified nucleotides	LNPs (proprietary, *Acuitas Therapeutics*), i.v., 2 mg/kg, 24 h prior to infection and 2 h post-infection	Swiss albino mice (protective efficacy was demonstrated under both prophylactic and therapeutic mRNA administration regimens in a lethal rabies infection model)	[270] (*CureVac, Acuitas Therapeutics*)
MERS-CoV	11	NbMS10 [271]	LNP-mRNA-NbMS10	Spike protein receptor-binding domain (RBD)	VHH single-domain antibody fused with the human IgG Fc region (chimeric dimer)	Expression of mRNA encoding the VHH single-domain antibody fused with the human IgG Fc region	LNPs (ionizable lipid/DOPE/cholesterol/PEG-lipid/targeting lipid at molar ratios of 24.5:4.7:20:0.8:50), i.v., 1 mg/kg	BALB/c mice (pharmacokinetic study and activity evaluation in an HIV-based MERS-CoV pseudovirus neutralization assay)	[175]
Hendra virus and Nipah virus	12	1E5 [272]	mRNA-1E5-LNPs	G glycoprotein	Full-length IgG antibody	Co-expression of mRNAs encoding HC and LC	LNPs (SM-102/DSPC/cholesterol/DMG-PEG2k at molar ratios of 50:10:38.5:1.5), i.v., up to 0.5 mg/kg, 12 h and 7 days prior to infection	BALB/c mice (protective efficacy was demonstrated under a prophylactic mRNA administration regimen in an infection model using the HIV-based Nipah and Hendra pseudoviruses)	[273]
Dengue virus	13	VDB11 ([274], Appendix A1)	scIgA mRNA	E glycoprotein	Full-length IgA1 antibody	Expression of mRNA encoding both HC and LC as a single-chain construct	LNPs (113-O10S/cholesterol/DOPC/DMG-PEG2k), i.v., 0.2 μg/kg	C57BL/6 (pharmacokinetic study)	[210]
Chikungunya virus	14	CHKV-24 [275]	mRNA-1944	Surface E2 glycoprotein	Full-length IgG antibody	Co-expression of mRNAs encoding HC and LC	LNPs (ionizable lipid/DSPC/cholesterol/DMG-PEG2k at molar ratios of 50:10:38.5:1.5), i.v.; AG129 mice: up to 0.5 mg/kg, 24 h prior to infection; C57BL/6 mice: up to 10 mg/kg, 4 h post-infection; cynomolgus macaques: up to 3 mg/kg; humans: up to 0.6 mg/kg	AG129 mice (protective efficacy was demonstrated under a prophylactic mRNA administration regimen in a lethal infection model); C57BL/6 mice (efficacy was assessed under a therapeutic regimen); cynomolgus macaques (pharmacokinetic studies); humans (Phase I clinical trial for safety, tolerability, pharmacokinetics, and pharmacodynamics)	[275,276] (*Moderna*)
Zika virus	15	ZIKV-117 [277]	ZIKV-117 RNA	Surface E protein dimer	Full-length IgG1 antibody or its scFv	Expression of the scFv; co-expression of two saRNAs encoding HC and LC; bicistronic saRNA with HC and LC separated by IRES; bicistronic saRNA with HC and LC separated by the furin cleavage site and the viral T2A peptide; both variants of chain orientations (HC–LC and LC–HC) in a single-chain construct have been tested	Nanostructured LNPs (squalene/Dynasan 114/Span 60/DOTAP), i.m., up to 2 mg/kg, 7, 5, and 1 days before infection or 24 h and 3 days post-infection	C57BL/6 mice (protective efficacy was demonstrated in both prophylactic and therapeutic regimens of saRNA administration in a lethal infection model using a mouse-adapted Zika virus strain, in combination with a single dose of a monoclonal antibody inhibiting IFNAR1)	[214]
Rift Valley fever virus	16	A38 [278]	A38-mRNA-LNP	Gn glycoprotein	Full-length IgG antibody	Co-expression of mRNAs encoding HC and LC	LNPs (ALC-0315/DSPC/cholesterol/PEG-lipid), i.m., 1 mg/kg	BALB/c mice (pharmacokinetic study)	[279]
Severe fever with thrombocytopenia syndrome virus (SFTSV)	17	Ab10 (S/A-TEN) [280]	mRNA S/A-TEN	Gn glycoprotein	Full-length IgG antibody	Co-expression of mRNAs encoding HC and LC	LNPs (ALC-0315/DSPC/cholesterol/DMG-PEG2k at molar ratios of 50:10:38.5:1.5), i.v., 3 mg/kg, 24 h post-infection, twice with a 3-day interval	C57BL/6 mice with transient blockade of interferon signaling using anti-IFNAR antibodies, IFNAR-knockout mice (protective efficacy was demonstrated under the therapeutic mRNA administration regimen in a lethal infection model)	[281]
Influenza A virus	18	—	Human IgG mRNA	—	Full-length IgG antibody	Co-expression of mRNAs encoding HC and LC	LNPs (ionizable lipid/DSPC/cholesterol/PEG-lipid at molar ratios of 50:10:38.5:1.5), i.v., 0.1 mg/kg	Cynomolgus macaques (pharmacokinetic study)	[282] (*Moderna*)
	19	FcγRIV VHH-M2e VHH [283] (FcγRIV VHH-RSVF VHH [284] used as a negative control)	FcγRIV VHH-M2e VHH RiboBiFEs (FcγRIV VHH-RSVF VHH RiboBiFEs)	Influenza virus M2e protein/mouse FcγRIV (RSV F glycoprotein)	Bispecific VHH single-domain antibodies	Expression of mRNA encoding bispecific VHH single-domain antibodies (RiboBiFE) as a single-chain construct	DOTAP/cholesterol-based LNPs at molar ratios of 2:3, i.t., 0.25 mg/kg, 4 h prior to infection	BALB/c mice, wild-type and FcγRIV^−/−^ C57BL/6 mice (protective efficacy was demonstrated under a prophylactic mRNA administration regimen in a lethal influenza A/X47 (H3N2) infection model)	[208]
	20	HV-B10 [285]	HV-B10 HC-P2A-LC mRNA/LNP	Hemagglutinin	Full-length IgG antibody	Expression of mRNA encoding both HC and LC as a single-chain construct	LNPs (ALC-0315: ALC-0315/DSPC/cholesterol/ALC-0159 at molar ratios of 46.3:9.4:42.7:1.6, i.v., i.m.; or MC3/DOTAP: Dlin-MC3-DMA/DSPC/cholesterol/DSPE-PEG2k/DOTAP at molar ratios of 25:5:19.2:0.8:50, i.n.), i.v., up to 0.5 mg/kg, 24 h prior to infection	C57BL/6 mice (protective efficacy was demonstrated under a prophylactic mRNA administration regimen in a lethal influenza A/California/04/2009 infection model)	[286]
Influenza B virus	21	CR8033 [287]	mRNA-LNP encoding anti-influenza B mAb	Hemagglutinin	Full-length IgG antibody	Co-expression of mRNAs encoding HC and LC; the mRNA did not contain modified nucleotides	LNPs (*Acuitas Therapeutics*), i.v., 2 mg/kg, 24 h prior to infection and 2 h post-infection	Swiss albino mice (used for pharmacokinetic studies and as a negative control for evaluating protective efficacy under prophylactic and therapeutic mRNA administration regimens in a lethal rabies infection model)	[270] (*CureVac, Acuitas Therapeutics*)
RSV	22	MEDI-493 (palivizumab) [46], F-VHH-4 [284]	sPali mRNA, aPali mRNA, aVHH mRNA, sVHH mRNA	F glycoprotein in a prefusion conformation	Full-length IgG antibodies: secreted (sPali, palivizumab) and GPI-anchored in HC (aPali); VHH single-domain antibodies: secreted (sVHH) and fused to a GPI anchor (aVHH)	Co-expression of mRNAs encoding HC and LC (for the assembly of both secreted IgG and the GPI-anchored forms); expression of mRNA encoding VHH single-domain antibodies (secreted or fused to a GPI anchor) as a single-chain construct	Viromer RED, in vivo-jetPEI, and unformulated mRNA in nuclease-free water; aerosol delivery using the MicroSprayer IA-1C device (*Penn-Century*), i.t., up to 5 mg/kg, administered 24 h and 7 days prior to infection	BALB/c mice (protective efficacy was demonstrated under a prophylactic mRNA administration regimen in an RSV infection model)	[177]
SARS-CoV-2	23	CB6 [288]	VEEV-VRP-CB6	Spike protein RBD	Full-length IgG antibody	Expression of saRNA encoding HC and LC separated by a subgenomic promoter; saRNA did not contain modified nucleotides	Virus replicon particles (VRPs), alphaviral particles, i.n., 5 × 10^5^ infectious particles, 24 h prior to infection	BALB/c mice (protective efficacy was demonstrated under a prophylactic saRNA administration regimen in a mouse-adapted coronavirus infection model)	[215]
	24	3E8 [289]	VEEV-VRP-3E8	Human angiotensin-converting enzyme 2 (hACE2)	Full-length IgG antibody	Expression of saRNA encoding HC and LC separated by a subgenomic promoter; saRNA did not contain modified nucleotides	Virus replicon particles (VRPs), alphaviral particles, i.n., 5 × 10^4^ infectious particles, 24 h prior to infection	Syrian hamsters (protective efficacy was demonstrated under a prophylactic saRNA administration regimen in an Omicron BA.1 infection model)	[216]
	25	HB27 [290]	mRNA-HB27-LNP	Spike protein RBD	Full-length IgG antibody	Co-expression of mRNAs encoding HC and LC	LNPs (ionizable lipid/DSPC/cholesterol/PEG-lipid at molar ratios of 50:10:38.5:1.5), i.v., up to 1 mg/kg, 24 h prior to infection	BALB/c mice (protective efficacy was demonstrated under a prophylactic mRNA administration regimen in a coronavirus infection model using the mouse-adapted MASCp36 strain and the SARS-CoV-2 Beta variant); Syrian hamsters (protective efficacy was demonstrated under a prophylactic mRNA administration regimen in a close-contact transmission model)	[291]
	26	XGv264 [292]	mRNA-XGv264-LNP	Spike protein RBD	Full-length IgG antibody	Co-expression of mRNAs encoding HC and LC	LNPs (novel ionizable lipid/DSPC/cholesterol/PEG-lipid), i.v., up to 1 mg/kg	Aged cynomolgus macaques (protective efficacy was demonstrated under a prophylactic mRNA administration regimen in an Omicron BA.1 coronavirus infection model)	[293]
	27	COV2-2832 [294], DH1041 [295]	COV2-2832 mRNA, DH1041 mRNA	Spike protein RBD	Two full-length IgG antibodies with the GPI-anchored HC	Co-expression of mRNAs encoding LC and the GPI-anchored HC (two GPI-anchored IgG studied individually)	Polymeric PBATE nanoparticles, i.n. using the Aerogen Solo nebulizer (*Tri-anim*), 2.5 mg/kg, 48 h prior to infection	Syrian hamsters (protective efficacy was demonstrated under a prophylactic mRNA administration regimen in a WA-1 coronavirus infection model)	[199]
	28	8-9D [174]	Liver-LNPs@mRNA^8-9D^, Lung-LNPs@mRNA^8-9D^s, Liver-LNPs@CircRNA^8-9D^, Lung-LNPs@CircRNA^8-9D^	Spike protein RBD	Full-length IgG antibody and chimeric scFv–Fc antibody	Co-expression of mRNAs encoding HC and LC; expression of circRNA encoding scFv–Fc antibody	LNPs (Liver-LNPs: ionizable lipid/DSPC/cholesterol/PEG-lipid at molar ratios of 49.1:9.4:40.0:1.5, Lung-LNPs: ionizable lipid/DOPE/cholesterol/PEG-lipid/Cationic targeting lipid at molar ratios of 24.5:4.7:20.0:0.8:50.0), i.v., 0.25 mg/kg, 24 h prior to infection and 24 h post-infection	K18-hACE2 transgenic mice (protective efficacy was demonstrated under both prophylactic and therapeutic mRNA administration regimens in a coronavirus infection model using the SARS-CoV-2 Beta and Omicron BA.2 variants)	[174]
	29	2NSP23 [296]	LNP-mRNA-2NSP23	Non-structural protein NSP9	VHH single-domain antibody	Expression of mRNA encoding a VHH single-domain antibody	LNPs (C12-200/DOPE/cholesterol/DMG-PEG/DOTAP at molar ratios of 29.8:13.6:39.5:2.1:15), ex vivo, up to 30 ng/μL, 24 h prior to infection	Human airway epithelial 3D cell cultures derived from the upper respiratory tract of healthy donors (viral replication was shown to be inhibited under a prophylactic mRNA administration regimen in a coronavirus infection model using multiple strains)	[297]
	30	LY1404 (bebtelovimab) [298], 76E1 [299]	LY1404 HC/LC mRNA-LNP, 76E1 HC/LC mRNA-LNP	Spike protein RBD S1 subunit (LY1404), FP fusion peptide of the spike protein S2 subunit (76E1)	Full-length IgG antibodies	Co-expression of mRNAs encoding HC and LC	LNPs (SM-102/DSPC/cholesterol/DMG-PEG2k at molar ratios of 50:10:38.5:1.5), i.m., 0.75 mg/kg, 24 h prior to infection	BALB/c mice (protective efficacy was demonstrated under a prophylactic mRNA administration regimen in a mouse-adapted CMA4 coronavirus infection model); Syrian hamsters (protective efficacy of a prophylactic mRNA administration regimen was demonstrated in two SARS-CoV-2 challenge models: one with the Omicron BQ.1 variant and another with the highly pathogenic Delta variant)	[300]
	31	PDI 204 [301]	PDI 204 HC-P2A-LC mRNA/LNP	Spike protein RBD	Full-length IgG antibody	Expression of mRNA encoding both HC and LC as a single-chain construct	LNPs (ALC-0315: ALC-0315/DSPC/cholesterol/ALC-0159 at molar ratios of 46.3:9.4:42.7:1.6, i.v., i.m.; or MC3/DOTAP: Dlin-MC3-DMA/DSPC/cholesterol/DSPE-PEG2k/DOTAP at molar ratios of 25:5:19.2:0.8:50, i.n.), i.v., up to 0.5 mg/kg, 7 days prior to infection	K18-hACE2 transgenic mice (protective efficacy was demonstrated under a prophylactic mRNA administration regimen in an Omicron BA.1 coronavirus infection model)	[286]

Abbreviations: LNPs, lipid nanoparticles; HC and LC, heavy and light immunoglobulin chains, respectively; i.v., intravenous; i.m., intramuscular; i.n., intranasal; i.t., intratracheal.

The remainder of Section 6 provides a systematic overview of contemporary approaches to the development of mRNA-encoded therapeutic antibodies against viral pathogens, the central focus of this review, drawing on over 30 in vivo studies. Viruses are grouped according to their primary routes of transmission, and within each group, individual pathogens are discussed in descending order of epidemiological relevance.

First, we address viruses transmitted predominantly through direct human-to-human contact (HIV-1 [302,303], hepatitis B virus [304,305], Ebola virus [306], and mpox virus [307,308,309]) via blood, bodily fluids, skin lesions, or close physical or sexual contact. In these settings, the principal therapeutic challenges include efficient neutralization of extracellular virions and protection of mucosal surfaces. Long-term efficacy is especially important for chronic infections such as HIV-1 and hepatitis B virus, whereas rapid neutralization is paramount for acute infections such as Ebola virus and mpox virus.

The discussion continues with zoonotic viruses, including rabies virus [310], MERS-CoV [311], Hendra virus [312], and Nipah virus [313]. These pathogens are maintained in animal reservoirs, with human infection typically occurring through direct contact with animals or their tissues. Such infections often involve cross-species transmission and high pathogenicity. In the case of MERS-CoV and Nipah virus, subsequent direct human-to-human transmission imposes additional constraints on therapeutic design.

The following subsection is devoted to arboviruses transmitted by arthropod vectors, including dengue virus [314,315], chikungunya virus [316,317], Zika virus [318,319], Rift Valley fever virus [320], and severe fever with thrombocytopenia syndrome virus (SFTSV) [321,322]. In addition to vector-borne transmission, these viruses may also spread vertically from mother to fetus (dengue virus, chikungunya virus, Zika virus), sexually (Zika virus), through contact-associated zoonotic routes (Rift Valley fever virus), or via direct human-to-human contact (SFTSV). As a result, therapeutic strategies for this group must emphasize rapid neutralization while accommodating substantial antigenic diversity.

The section concludes with respiratory viruses transmitted via droplets and aerosols, including influenza A and B viruses [323], RSV [324], and SARS-CoV-2 [325,326,327,328]. This group appears the most promising in terms of developing universal or platform-based antibody solutions suitable for large-scale prophylaxis and treatment during seasonal epidemics and global pandemics.

Overall, this framework illustrates how pathogen biology and transmission dynamics shape target selection, define therapeutic requirements, and guide strategic priorities in the development of mRNA-encoded antibodies.

### 6.2. mRNA-Encoded Antibodies Against Viruses Transmitted via Direct Human-to-Human Contact

(1, as listed in Table 3) In 2017, a team of US and Canadian scientists, led by the 2023 Nobel prize winner Drew Weissman, published the first paper on mRNA application for passive immunotherapy [255]. They evaluated the practical feasibility of translating exogenous mRNAs with modified bases into a full-length IgG with broad neutralizing activity in mice. This mRNA sequence encoded the heavy (HC) and light (LC) chains (Figure 2A) of the VRC01 antibody [254] against HIV-1. Twenty-four hours after administering a single 1.4 mg/kg i.v. dose of mRNA–LNPs in humanized immunodeficient mice, approximately 170 μg/mL of the VRC01 protein was detected in blood serum, a level that exceeded the concentration achieved when the same antibody was injected in its protein form (600 μg). High levels of the mRNA-encoded antibody persisted for 5 days. Combined i.v. delivery of LNPs and mRNA with modified nucleotides, purified by chromatography, did not increase type I interferon or pro-inflammatory cytokine production. Weekly injections of mRNA–LNPs–VRC01 at 1 mg/kg maintained serum antibody levels at or above 40 μg/mL. The antibodies generated after a single mRNA injection efficiently suppressed viral replication, protecting mice from infection after i.v. HIV-1 challenge. This study was the first in vivo experiment to show the strong therapeutic potential of mRNA-encoded antibodies.

(2) Three years later, Philip Santangelo’s team from Georgia Institute of Technology and Emory University, USA, evaluated the activity of an mRNA-encoded antibody [178] based on the broadly neutralizing antibody (bnAb) PGT121 that targets the HIV-1 surface glycoprotein gp120 [83]. The therapeutic mRNA PGT121 formulation was administered to the cervix of sheep, which is anatomically similar to the human cervix, as an aqueous spray containing either the secreted form of the mRNA-encoded antibody (sPGT121) or the antibody carrying a GPI anchor (aPGT121) (Figure 2A,C). To compare the pharmacokinetics and distribution of PGT121 on the mucosal surface over time, the authors used chimeric mRNA constructs that encoded the HC conjugated with NanoLuc. The results were also confirmed on epithelial samples from the female genital tract using Western blotting [178]. Pharmacokinetic studies using a single 750 μg dose showed that the GPI anchor in aPGT121 promoted high and sustained antibody expression in the reproductive tract. Antibody levels peaked at 100 μg/mL at 24 h and then gradually decreased to 40 μg/mL by day 28. In contrast, sPGT121 declined to about 10 μg/mL by day 14. Thus, introducing the GPI anchor extended antibody expression and improved its efficiency in topical application. Additionally, to assess the neutralizing activity of mRNA-encoded antibodies ex vivo, female monkeys received several doses of aPGT121 mRNA, and biospecimens were collected 24 h later. Explants and vaginal secretions containing the antibody demonstrated high antiviral activity in neutralization reactions with simian HIV. This work also showed that an aqueous mRNA formulation applied as a spray could potentially be used for self-administered prophylaxis.

(3) The study by *Moderna* [207] used the mRNA–LNPs platform to co-express three bnAbs, PGDM1400, PGT121, and N6, which target the HIV-1 envelope protein [79,83,84]. Single-chain scFv–Fc antibodies were designed based on the sequences of the corresponding full-length immunoglobulins. Each construct contained the variable regions of HC and LC, as well as the IgG Fc region composed of the CH2 and CH3 heavy-chain domains. Two of the three antibodies, PGDM1400 and PGT121, showed neutralizing activity comparable to that of the original IgG, and their expression levels in mice exceeded 30 μg/mL. In contrast, the N6 antibody was functional only as a full-length IgG, reaching a peak concentration of approximately 1 mg/mL. A mixture of four mRNAs encoding the HC and LC of N6 (Figure 2A) and the scFv–Fc forms (Figure 2D) of PGDM1400 and PGT121 was efficiently expressed in Tg32 hemizygous mice [329], inducing high IgG levels in blood. Antibody levels above 10 μg/mL were observed for 90 days after i.v. delivery of the 1 mg/kg mRNA cocktail. Sera from these immunized mice exhibited neutralizing activity against multiple HIV-based pseudovirus strains.

(4) The latest study from the Santangelo group [180], published in November 2025, introduces an innovative strategy to advance HIV prevention through intravaginal delivery of mRNA encoding bnAbs against HIV-1 surface proteins. Central to this work is the development of a SC-mRNA platform (Figure 2B) that enables more efficient assembly of antibody HC and LC compared to conventional dual-chain systems. This design reduces the likelihood of non-functional antibody species and free light chains. The authors used previously reported anti–HIV-1 bnAbs to demonstrate feasibility in vitro. These bnAbs were encoded by mRNA either in a secreted IgG format or as membrane-anchored antibodies via a GPI anchor, including PGT121 [83], VRC07 [80], 10E8.4 [256], iMab [257], CAP256 [258], and PGDM1400 [84] (Figure 2C). Based on in vitro neutralization profiles, the study selected PGT121 and VRC07 for in vivo evaluation, as these antibodies exhibited the broadest activity and targeted distinct epitopes on the HIV-1 gp120 envelope glycoprotein.

Building on the SC-mRNA platform, the study evaluated multiple strategies to enhance neutralizing potency. These included co-expression of two bnAbs (PGT121 and VRC07) (Figure 2B), isotype switching from IgG to IgA (Figure 2F), extension of the IgG constant region with an IgM tailpiece (Figure 2G) to promote multimer formation while preserving variable domains, and structural engineering of J3-VHH single-domain antibody (Figure 2E) [259] to increase antigen-binding valency and incorporate an IgG Fc domain.

Earlier work from *Moderna* [207] generated functional mRNA constructs encoding single-chain Fv–Fc fusion proteins that lacked the CH1 constant domain. In contrast, the SC-mRNA system described by Santangelo and colleagues [180] retains full-length IgG constant regions, including CH1, which may be instrumental in regulating variable-domain affinity. To further support multimerization, the authors introduced mRNA encoding the JC, a critical component in the formation of multimeric IgA and IgM. Co-transfection of antibody-encoding SC-mRNA with JC mRNA enabled efficient generation of multimeric antibody species (Figure 2F,G). Multimeric antibodies may exhibit enhanced therapeutic efficacy due to their increased avidity and improved effector functions.

These structural modifications of mRNA-encoded bnAbs, designed to enhance mucosal immunity [330,331], markedly expanded neutralization breadth and increased potency compared with parental IgG-format bnAbs. The most pronounced effects were observed when PGT121 and VRC07 were co-expressed as IgM-like multimers (Figure 2G). These engineered antibodies neutralized a broad panel of SHIV/HIV isolates, including clades A, B, C, and AE, many of which resisted neutralization by IgG-format bnAbs.

In rhesus macaques, a single intravaginal aerosol administration of unformulated mRNA in nuclease-free water encoding IgM-like multimeric PGT121 and VRC07 induced sustained local antibody production at the vaginal mucosa. This approach provided near-complete tissue protection during ex vivo challenge with all tested SHIV strains, including SHIV-KNH1144, SHIV-162, SHIV-C109, and SHIV-AE16. Comprehensive safety assessments detected no significant systemic or local toxicity, immune activation, or tissue damage at 24 h post-administration. Parallel experiments in mice further supported these findings. Administered on day 0 and day 2, two intravenous doses of mRNA-LNPs encoding single-chain PGT121 and VRC07 in secreted IgA or multimeric IgM formats produced robust serum antibody levels, reaching 19.2 µg/mL for IgA and 82.8 µg/mL for IgM by day 3.

Thus, the study demonstrated that a SC-mRNA antibody platform, combined with strategic structural design, can overcome key limitations of existing HIV-1 prevention strategies. This work establishes a promising foundation for the development of effective and safe mucosal prophylactics against HIV, particularly for women, and suggests broad potential for adaptation to other sexually transmitted infections.

(5) In 2026, Davin Sok’s group, in collaboration with researchers from *Moderna*, reported a successful application of mRNA–LNPs encoding both IgG1 (Figure 2A) and IgA2 (Figure 2F) isotypes of the monoclonal ePGDM1400v9 antibody against HIV-1 in rhesus macaques [209]. ePGDM1400v9 is an optimized antibody derived from the bnAb PGDM1400 described earlier [84]. An M428L/N434S mutation (LS mutation) [332] was introduced into the Fc region of IgG1 to increase its half-life. Three separate mRNA molecules encoding the heavy, light, and J-chains, which are required to form IgA2 dimers, were used to produce IgA2. After i.v. administration of mRNA–LNPs, the corresponding serum antibodies were detected in all animals. Peak levels were significantly higher for IgG1 than IgA2 (23.7–34.5 µg/mL vs. 1.4–5.4 µg/mL), and IgG1 had a longer half-life compared to IgA2 (7.3–15.4 days vs. 3.4–3.6 days). However, both immunoglobulin isotypes retained HIV-neutralizing activity in vitro, similar to their recombinant analogs. Mass spectrometry analysis revealed that mRNA-encoded IgA2 antibodies carried a native glycosylation pattern, including N-glycolylneuraminic acid, which is not usually present in recombinant antibodies produced in CHO or HEK cell lines. These findings point to the potential of the mRNA platform to express functionally active IgA-isotype antibodies with characteristics similar to native immunoglobulins.

(6) In China, Tianlei Ying and collaborators explored three homologous antibodies against the hepatitis B surface antigen S protein (HBsAg): G12-scFv, G12-scFv–Fc, and G12-IgG [261]. The G12 antibody was developed as a candidate drug against chronic hepatitis B infection [260]. Two formats of this antibody, G12-scFv–Fc (Figure 2D) and G12-IgG (Figure 2A), were tested in a murine model of infection. After a single i.v. administration of mRNA–LNPs at 2.5 mg/kg, serum HBV DNA and HBsAg levels were significantly reduced for 30 days in mice infected with an adeno-associated virus expressing HBsAg, which indicates sustained passive immunity. By comparison, the recombinant G12 antibody at a dose of 6.7 mg/kg became ineffective as early as 9 days after administration.

(7) Wei Chen’s team from the Laboratory of Advanced Biotechnology in Beijing [263] developed and evaluated mRNA–LNPs as a potential emergency prophylaxis agent against hemorrhagic fever caused by Ebola virus. The mRNA molecules encoded the HC and LC (Figure 2A) of a previously isolated universal human bnAb 2G1, which targeted the viral surface glycoprotein and recognized four *Ebolavirus* species [262]. The animals received up to 1 mg/kg of mRNA–2G1–LNPs via i.v. injection and up to 5 mg/kg of the 2G1 protein 12 h before intraperitoneal injection (i.p.) of pseudoviruses. Delivered as mRNA–2G1–LNPs, this antibody demonstrated high neutralizing activity against HIV-pseudotyped Ebola virus (EBOV) and Sudan virus (SUDV) in mice: 19.8-fold and 12.5-fold higher, respectively, than that of the original recombinant 2G1 IgG protein. The antibodies encoded by mRNA–2G1 peaked at 24 h, which effectively prevented the invasion of pseudoviruses. The minimum prophylactic dose of these mRNA–LNPs that completely suppressed pseudovirus infection in mice was 0.25 mg/kg.

(8) Researchers from the U.S. Army Medical Research Institute of Infectious Diseases (USAMRIID), in collaboration with the German company *CureVac*, evaluated mRNA-encoded antibodies (Figure 2A) against poxviruses in macaques and rabbits [267]. They explored the feasibility of using unmodified mRNAs for passive immunotherapy. These mRNAs encoded three previously described chimeric antibodies, c7D11, c8A, and c6C [264,265,266], which target poxviruses, including variola virus (VARV), vaccinia virus (VACV), and mpox virus (MPXV). The mRNA constructs were delivered within a relatively short time frame via several sequential injections. High antibody concentrations were detected 24 h after i.m. administration of mRNA–LNP complexes to rabbits at doses up to 0.9 mg/kg. However, pharmacokinetic studies suggested that these mRNA-encoded antibodies might fail to provide effective protection against viral infection in large laboratory animals due to their short circulation half-life.

(9) A group of Chinese researchers led by Cheng-Feng Qin explored several combinations of mRNA-encoded antibodies with broadly neutralizing activity against poxviruses [268]. In vivo work resulted in the development of an anti-smallpox cocktail comprising two therapeutic mRNA-encoded antibodies, mAb22 and mAb26. These antibodies targeted two major antigens present during infection: the intracellular mature virion (IMV) and the extracellular enveloped virion (EEV). Antibody expression was achieved using a dual HC/LC mRNA system (Figure 2A): mRNAs encoding the HC and LC of each antibody were separately encapsulated in LNPs and then combined as a cocktail for administration to animals. Antibody concentrations peaked one day after mRNA administration in mice. A single i.v. injection of the antibody cocktail at a dose of 1 mg/kg one day prior to infection prevented weight loss and mortality, as shown using the lethal cowpox infection model.

### 6.3. mRNA-Encoded Antibodies Against Zoonotic Viruses Transmitted Through Direct Contact

(10, 21) An international team from Tufts University (USA), *Acuitas Therapeutics* (Canada), and *CureVac* (Germany) investigated mRNAs encoding full-length antibodies (Figure 2A) that provided protective immunity against rabies and influenza B in a lethal mouse model [270]. In the prophylactic regimen, mice received a single i.v. dose of mRNA–LNPs (2 mg/kg) encoding the CR57 anti-rabies antibody [269], and 24 h later, were challenged with rabies via i.m. injection. In the therapeutic regimen, animals received mRNA–LNPs 2 h after infection. No lethal outcomes were registered in the groups treated with anti-rabies mRNA–LNPs, whereas the control group that received mRNA–LNPs encoding anti-influenza antibodies [287] showed 100% mortality.

(11) mRNA-encoded antibodies have also been designed to target the spike protein RBD of the MERS-CoV virus, causing Middle East Respiratory Syndrome [175]. To improve antibody stability and reduce reactogenicity, Guangyu Zhao’s team from China developed chimeric dimerized antibodies consisting of the NbMS10 VHH single-domain antibody (Figure 2E) [271] fused to the Fc region of human IgG. mRNA was packaged in LNPs for targeted delivery to the lungs. In a pharmacokinetic study, mice received the mRNA-encoded antibodies at a dose of 1 mg/kg via i.v. injection. The results confirmed efficient lung targeting and high expression of the chimeric antibody, which persisted for 24 h. It was also shown that sera from immunized mice had virus-neutralizing activity against a MERS-CoV pseudovirus.

(12) A 2025 study by Changming Yu’s team from Beijing Institute of Biotechnology [273], who previously developed mRNA-encoded antibodies against Ebola virus [263], evaluated the stability, efficacy, and therapeutic potential of mRNA-encoded antibodies for henipavirus infections. The team screened UTRs and identified an optimal combination for mRNA-mediated expression of the bnAb 1E5 [272], which targets the G glycoproteins of Nipah and Hendra viruses. The resulting mRNA–LNPs–1E5 formulation (Figure 2A) produced high levels of functional antibody after i.v. administration in mice. Antibody levels peaked at day 3 post-injection with no signs of hepatotoxicity or tissue inflammation. In two mouse infection models using Nipah and Hendra pseudoviruses, prophylactic mRNA administration demonstrated high protective efficacy: both at a low dose (0.125 mg/kg) given 12 h before challenge and at a higher 0.5 mg/kg single dose of mRNA, stored at 4 °C for two months and administered 7 days prior to infection.

### 6.4. mRNA-Encoded Antibodies Against Viruses with Vector-Borne Transmission

(13) The protective potential of candidate therapeutics based on mRNA-encoded antibodies has been explored for several arboviruses as well [333]. Published in February 2025, the paper by the Waickman lab was the first to describe the design of antiviral mRNA-vectored antibodies of the IgA isotype (Figure 2F) [210]. Before this work was released, the only study on the topic was published in 2023 by *Moderna*. It investigated the specific activity of in vivo mRNA-encoded IgA antibodies targeting bacterial pathogens [211]. Waickman and colleagues worked with VDB11 [274], a bnAb they previously developed against the viral E glycoprotein of dengue virus, which is transmitted by mosquitoes. This is particularly relevant for developing a safe strategy to prevent and treat dengue fever, as, in this case, IgG antibodies should be avoided due to the risk of antibody-dependent enhancement (ADE). The researchers used several strategies to design constructs that expressed anti-dengue antibodies from a single-chain IgA-encoding segment (scIgA). In particular, they tested antibody variants with different VL and VH fragment orientations and hinge regions derived from both IgA and IgG isotypes. In mice, i.v. administration of mRNA–LNPs–scIgA induced sustained production of human IgA antibodies for 3–4 days. These serum antibodies were specific to the dengue virus E protein, with a half-life of approximately 19 h.

(14) The first and, to date, the only mRNA-based antiviral therapeutic to enter clinical trials is mRNA-1944 developed by *Moderna* [334]. It encodes the HC and LC domains of the full-length IgG antibody (Figure 2A) CHKV-24 that targets the mosquito-borne chikungunya virus, and has successfully completed Phase I. The highly neutralizing CHKV-24 antibody against the E2 glycoprotein was isolated from B cells of a recovered patient [275]. Administration of CHKV mRNA–LNPs–24 via i.v. injection in mice at a dose of 200 μg induced high levels of bnAbs within 24 h and prevented mortality, as well as virus-induced arthritis and musculoskeletal disorders. In cynomolgus macaques, i.v. delivery of CHKV-24 mRNA–LNPs at 0.5 mg/kg achieved therapeutically significant serum concentrations of up to 36 μg/mL, with a half-life of 23 days. These results have supported the progression of CHKV-24 in mRNA format to clinical trials.

In a randomized, placebo-controlled phase I clinical trial evaluating the safety, tolerability, pharmacokinetics, and pharmacodynamics of mRNA-1944, 38 healthy participants aged 18–50 received LNP-mRNA-1944 complexes either as a single dose of 0.1, 0.3, or 0.6 mg/kg, or as two weekly doses of 0.3 mg/kg. No serious adverse events were observed after mRNA administration. CHKV-24 IgG expression was dose-dependent, detectable 12, 24, and 48 h after a single dose, and reached therapeutically relevant levels (>1 μg/mL) with a half-life of approximately 69 days. Administration of mRNAs encoding the HC and LC of CHKV-24 IgG induced high circulating levels of functional bnAbs that exceeded expected protective titers while remaining safe and persisting for several months [276]. Re-administration one week later almost doubled serum antibody concentrations.

(15) saRNA technology [212,213] was employed to express a full-length human monoclonal antibody ZIKV-117 against the Zika virus, also a mosquito-transmitted pathogen. ZIKV-117 has broad neutralizing activity against both African and Asian lineages of the virus [277]. A group of American researchers led by Neal van Hoeven designed an saRNA replicon containing the genes for four non-structural proteins of Venezuelan equine encephalitis virus (VEEV; nsP1–nsP4) [335]. The downstream genes for viral structural proteins were replaced with sequences encoding the HC and LC of ZIKV-117 (Figure 2H) [214]. To enable full-length antibody expression, four different strategies were applied: (1) separate saRNAs encoding HC and LC individually; (2) an saRNA sequence encoding the ZIKV-117 scFv; (3) a single-chain saRNA encoding HC and LC separated by an encephalomyocarditis virus (EMCV) internal ribosome entry site (IRES); (4) a single-chain saRNA encoding HC and LC separated by a furin cleavage site and a viral T2A peptide in order to enable ribosomal skipping (Figure 2H). The last two strategies were tested for both orientations of the antibody chains, e.g., H-IRES-L and L-IRES-H.

The study showed that ZIKV-117 serum levels were dose-dependent up to 10 μg of saRNA and then plateaued, even at the highest tested dose of 200 μg. When delivered intramuscularly in mice, saRNA encoding HC and LC separated by the furin-T2A sequence resulted in 32-fold higher antibody levels compared to conventional mRNA encoding the same antibody. Peak concentrations of various saRNA-encoded antibodies were observed 5–7 days after saRNA administration. Suppression of the immune response through interferon-alpha receptor (IFNAR) inhibition was initially developed to create a lethal mouse model of Zika virus infection [336]. It is worth noting that prophylactic IFNAR blockade one day before infection significantly enhanced saRNA-mediated expression of ZIKV-117. Overall, i.m. administration of saRNA encoding neutralizing ZIKV-117 antibodies demonstrated strong protective potential in a lethal mouse model, under both prophylactic and therapeutic regimens.

(16) Based on the previously patented bnAb A38 [278], a research group from Zhejiang University, led by Jianmin Li, developed an mRNA–LNPs formulation to deliver an antibody against another mosquito-transmitted pathogen—Rift Valley fever virus. They compared the pharmacokinetics of the antibody in mice following i.m. injection of the mRNA-encoded form (Figure 2A) with i.v. administration of the protein form [279]. A 1 mg/kg dose of the mRNA-encoded antibody provided a longer bloodstream half-life than the protein injection, persisting for more than 20 days.

(17) In 2025, a South Korean research group led by Jae-Hwan Nam developed an mRNA-based drug [281] that encodes the bnAb Ab10 (Figure 2A) [280] against the Gn glycoprotein of SFTSV, which is transmitted through tick bites. A single i.v. dose of A–TEN–mRNA–LNPs in mice induced rapid antibody expression, peaking at 6 h, and generated high neutralizing titers against multiple viral strains. In a therapeutic regimen, the treatment provided 100% protection in two lethal infection models: in C57BL/6 mice with a temporary interferon blockade using anti-IFNAR antibodies, and in immunodeficient C57BL/6 IFNAR-knockout mice. The mRNA-encoded antibodies were administered twice, 3 days apart, starting 24 h after infection. This regimen completely prevented mortality, promoted rapid body weight recovery, significantly reduced viral loads in organs, and prevented liver and spleen pathology, indicating its strong therapeutic potential.

### 6.5. mRNA-Encoded Antibodies Against Airborne Viruses

(19) Xavier Saelens’ team from Belgium and German collaborators developed a chimeric construct consisting of two bispecific single-domain llama antibodies (VHH). One VHH single-domain antibody targeted the ectodomain of the influenza A virus M2 protein (M2e), and the other targeted the murine Fcγ receptor IV (FcγRIV) [283]. Bispecific antibodies can engage two different antigenic epitopes, which can broaden their therapeutic effect compared to conventional monoclonal antibodies [195,196,197,198]. This chimeric antibody was designed to selectively recruit innate immune cells to influenza-infected cells by bridging the conserved M2e region, exposed on the surface of infected cells, with the activating FcγRIV. FcγRIV, which is expressed on monocytes, macrophages, and neutrophils, plays an important role in IgG2α-dependent effector activity in vivo [337]. In this way, the genetically engineered antibody helps direct innate immune cells to sites of viral replication [338]. RiboBIFE, a related class of mRNA-encoded antibodies consisting of two VHH single-domain antibodies (Figure 2E) [208], were encapsulated into DOTAP/cholesterol-based particles. After i.t. administration, RiboBIFE remained detectable in bronchoalveolar lavage fluid for 48 h, whereas the corresponding protein antibody was no longer detectable after 24 h. In vivo, mice given RiboBIFE one day before infection with a lethal dose of influenza A virus lost less weight, showed higher survival rates, and had lower viral loads in the lungs compared to controls.

(22) mRNA technology has also been used to express palivizumab, the first monoclonal antibody approved for the prevention of a viral infection. It was licensed in 1998 for prophylaxis against RSV in high-risk pediatric populations [46,47,324] and has since shown effectiveness in clinical practice. The Santangelo group evaluated several antibody variants encoded by exogenous mRNA [177]: soluble palivizumab (sPali) (Figure 2A); a membrane-anchored form of palivizumab fused to a GPI anchor appended to an immunoglobulin HC (aPali) (Figure 2C); a secreted VHH single-domain antibody (Figure 2E) against the RSV F glycoprotein (sVHH; heavy chain variable domains of llama antibodies), originally developed by Xavier Saelens’ team [284]; and a membrane-anchored version of the same VHH single-domain antibody (aVHH). In mice, prophylactic administration of 5 mg/kg of sPali or aPali as an aqueous mRNA spray reduced RSV titers in the lungs by 65% and 89%, respectively. The aqueous mRNA spray was also more effective than commercial liposomal carriers and did not elicit an immune response. These results clearly demonstrated that GPI anchors can be used to increase the bloodstream circulation time of small single-domain antibodies such as VHH, which typically have short half-lives (30 min to 2 h). In this study, the aVHH single-domain antibody remained detectable in the lungs for 28 days after i.v. administration of the exogenous mRNA. It also significantly lowered RSV titers in infected mice 7 days after mRNA delivery. Administration of 100 μg of mRNA encoding either secreted or membrane-anchored palivizumab provided the same level of protection as an i.m. dose of the commercial protein form of palivizumab. mRNA encoding anchored antibodies enables immunoglobulins to remain on the epithelial surface, increasing their concentration in the lungs and improving efficacy. Overall, these findings indicate that mRNA-encoded antibodies can prevent RSV infection, with membrane-anchored variants producing stronger antiviral effects than their secreted counterparts.

*mRNA-Encoded Antibodies Against SARS-CoV-2*

(23) Since 2020, numerous studies have focused on the development of exogenous mRNA encoding antibodies against SARS-CoV-2. Led by Han-Qing Ye, a research team from Wuhan Institute of Virology in China [215] designed a construct based on a modified self-amplifying VEEV alphavirus replicon with two subgenomic promoters (Figure 2H). This enabled simultaneous translation of HC and LC and the assembly of the full-length bnAb CB6 against the SARS-CoV-2 spike protein RBD [288]. VEEV-VRPs (alphavirus replicon particles) were used for targeted delivery to the lungs via i.v. injection in mice. This saRNA protected mice from infection in the prophylactic administration regimen, as reflected by lower viral titers and reduced lung tissue damage. (24) Furthermore, i.n. delivery of saRNA encoding the 3E8 antibody against human angiotensin-converting enzyme [289] protected hamsters from both the Omicron and Delta strains of coronavirus [216].

(25) Another study by Cheng-Feng Qin’s group from China [291] showed that mRNAs encoding the HC and LC (Figure 2A) of the HB27 bnAb [290] against the SARS-CoV-2 spike protein RBD produced antibodies with an extended half-life. A single prophylactic i.v. dose of this mRNA–LNPs formulation provided a 50% survival rate in mice challenged with a lethal dose of the virus for up to 63 days, whereas the protein form of HB27 administered in the same regimen was associated with 100% mortality as early as 9 days after infection. In a prophylactic administration regimen, dose-dependent protection was also observed in a close-contact transmission model in hamsters.

(26) In a follow-up study [293], Qin and colleagues described mRNA (Figure 2A) encoding another bnAb against the SARS-CoV-2 spike protein RBD—XGv264 [292]. A single i.v. administration of mRNA–LNPs–XGv264 in cynomolgus macaques resulted in rapid and sustained antibody expression, with peak serum concentrations of 9.6 µg/mL and a half-life of 16.2 days. These antibodies exhibited broad neutralizing activity against various SARS-CoV-2 variants, including Omicron BA.1 and BA.2. Prophylactic administration of mRNA–LNPs–XGv264 to aged macaques at 0.3 and 0.6 mg/kg two days before infection provided dose-dependent protection. It completely suppressed viral RNA shedding from both the upper and lower respiratory tract, significantly decreased viral loads in the lungs, and reduced lung pathology. This mRNA-based drug demonstrated a satisfactory safety profile, leading to only minor and reversible changes in certain hematological parameters.

(27) The Santangelo group also explored mRNA-based therapeutics against SARS-CoV-2. In their 2022 study, they developed an inhalable, nebulized form of mRNA-encoded antibodies against the spike protein RBD, which contained a GPI anchor in the HC to tether the antibody to cell membranes (Figure 2C) [199]. mRNA encoding neutralizing monoclonal antibodies COV2-2832 [294] and DH1041 [295] was encapsulated in a PBATE polymer [179] for targeted delivery to the lungs. Antibody expression mediated by this mRNA persisted in the lungs of hamsters for several weeks. Nebulized mRNA-encoded antibodies achieved higher neutralizing activity at lower doses than i.v. delivery of the corresponding protein antibodies. In a hamster coronavirus infection model, prophylactic administration of 312 μg of mRNA two days before infection had a significant positive effect on animal weights and lung viral titers.

(28) Gong Cheng’s team from Tsinghua University in Beijing focused on lung-selective delivery of mRNA encoding the human bnAb 8-9D against various SARS-CoV-2 variants (Figure 2A) [174]. In particular, the antibody expression efficiency was evaluated in K18-hACE2 transgenic mice following i.v. administration of mRNA encapsulated in LNPs, either selectively targeted to the lungs or predominantly delivered to the liver. Lung-targeted delivery of the 8-9D mRNA enabled the expression of bnAbs in the lungs, which blocked virus invasion and effectively protected animals from infection with the Beta or Omicron BA.1 coronavirus variants. Both mRNA–LNP formulations significantly reduced viral loads in the lungs and trachea compared to the untreated group.

Importantly, in this study, the authors also reported the first—and, thus far, only—application of circRNA technology [217,218,219,220] for targeted expression of therapeutic antibodies against viruses. They engineered a circRNA (Figure 2I) encoding the 8-9D antibody in a single-chain scFv–Fc format, encapsulated in LNPs, and showed it to be effective in vivo. The enhanced stability of circRNA extended the half-life of the 8-9D antibody in mouse lungs and bronchoalveolar lavage fluid to nearly two weeks. As of February 2026, only one additional study has reported another application of the novel circRNA–LNP–based platform for sustained antibody expression. In a colorectal cancer model, prolonged expression of a circRNA-encoded bispecific antibody targeting PD-L1 and CD3 resulted in robust therapeutic efficacy [339].

(29) In this review, we do not cover studies that evaluated the antiviral activity of mRNA-encoded antibodies only in vitro. Still, we would like to highlight a study led by Piergiorgio Percipalle and an international team from Belgium, Germany, Italy, and the UAE [297], as they were the first to experimentally validate the concept of therapeutic mRNA-encoded intrabodies directed against viral non-structural proteins [230]. Before this work, several research groups had already evaluated various formats of plasmid-encoded intrabodies against non-structural proteins of influenza virus in vitro [340,341,342,343]. In 2024, the Percipalle lab assessed the antiviral activity of the mRNA-encoded 2NSP23 VHH single-domain antibody (Figure 2E) [296] targeting the highly conserved SARS-CoV-2 intracellular non-structural protein NSP9, which plays a key role in the assembly of the viral replication-transcription complex (RTC). Delivery of mRNA–LNPs–2NSP23 to infected cells induced intracellular expression of the single-domain antibody that specifically bound NSP9. This interaction suppressed the replication of multiple SARS-CoV-2 variants, including Hu-1, Alpha, Delta, Mu, and Omicron. It also restored the host cell transcriptional program disrupted by the virus, such as genes linked to mitochondrial function and antiviral immune response. The effectiveness of this approach was confirmed in both cell culture and a physiologically relevant 3D model of reconstructed human respiratory epithelium [344], suggesting its potential as a new strategy for the development of antiviral mRNA therapeutics.

(30) In their 2026 study [300], Haitao Hu’s team from the University of Texas Medical Branch describe an mRNA–LNP complexes encoding two bnAbs against SARS-CoV-2 (Figure 2A): LY1404 [298], targeting the RBD within the spike protein S1 subunit, and 76E1 [299] directed at the fusion peptide (FP) epitope within the spike protein S2 subunit. A single i.m. injection of mRNA–LNPs 24 h before infection resulted in efficient serum antibody production in mice, which peaked on day 3 and was sustained for 7–14 days. Both mRNA-encoded antibodies significantly reduced viral loads in the lungs of mice challenged with an adapted SARS-CoV-2 strain (CMA4). In hamsters infected with the immune-evasive Omicron BQ.1 or the highly pathogenic Delta variant, however, only the 76E1 antibody provided significant protection. It reduced viral load both locally in the upper respiratory tract (nasal washes) and in the lungs, while also preventing lung pathology. These results demonstrate that mRNA-delivered bnAbs, particularly 76E1, confer protection against SARS-CoV-2 infection by reducing both viral burden and disease severity.

(31, 20) A recent study from Adam Wheatley’s group at the University of Melbourne [286] describes an SC–mRNA–LNPs platform (Figure 2B) designed for efficient delivery and in vivo co-expression of two bnAbs: PDI 204 [301], which targets the SARS-CoV-2 spike protein RBD, and HV-B10 [285], which targets influenza A virus hemagglutinin. A single i.v. or i.m. administration of these mRNA–LNPs in mice produced high levels of functional antibodies in both blood serum and the respiratory mucosa. Moreover, the construct that contained a SC-mRNA encoding the HC and LC linked by a P2A peptide was more effective than using separate mRNAs for each chain. A key limitation, however, was the induction of anti-drug antibodies (ADA) against the therapeutic, triggered by the adjuvant properties of the LNPs. ADA promoted faster clearance of the mRNA-encoded antibodies from the bloodstream compared with recombinant antibodies. This, in turn, directly affected protective efficacy: when given one week before challenge, the mRNA–LNPs provided weaker protection from SARS-CoV-2 than the corresponding protein antibodies, whereas the mRNA-encoded antibody HV-B10 administered one day before challenge offered complete protection against a lethal influenza infection. Overall, the study reinforced the potential of mRNA–LNPs for rapid antibody delivery, yet emphasized the need to optimize LNP formulations to reduce the reactogenicity of mRNA-based therapeutics and prolong their activity.

mRNA-encoded antibodies as a therapeutic modality are advancing exceptionally rapidly, and new studies continue to emerge even during the preparation of this review. Notably, just a week before our manuscript was accepted, the first antiviral intrabody with demonstrated in vivo efficacy was reported in a study by Shaowei Li’s team from Xiamen University in China. Their work described intrabody 17F2, delivered in an mRNA–LNP–scFv format [345]. This antibody targets human papillomavirus type 18 (HPV18) [346] and recognizes a previously uncharacterized α3 helix in the C-terminal region of the intracellular oncoprotein E7. Functionally, mRNA-encoded scFv-17F2 inhibited the proliferation of HPV18-positive HeLa cells in vitro and suppressed tumor xenograft growth in immunodeficient mice in vivo, with enhanced efficacy observed for constructs incorporating a nuclear localization signal.

Taken together, recent studies have clearly demonstrated the biological activity of therapeutic mRNAs that encode recombinant antiviral antibodies in various formats. Several strategies have been suggested and validated for in vivo production of antibodies encoded by exogenous mRNAs, highlighting the pharmacokinetic advantages of this approach compared to protein-based immunoglobulin therapeutics. These findings undoubtedly establish mRNA-based recombinant antibody therapy as a promising platform for developing novel antiviral strategies.

## 7. Discussion

For more than a century, convalescent serum from humans or animals has been used to prevent and treat infectious diseases. As a therapeutic approach, passive antibody transfer gained new momentum once immunoglobulins were characterized and monoclonal antibody technology emerged. These advances reshaped immunology and laid the groundwork for the discovery of a novel class of therapeutics: monoclonal antibodies. Recreating a natural, immune-derived ‘therapeutic’ that undergoes strict selection in vivo and binds foreign antigen epitopes with high specificity proved to be an extremely promising direction for antiviral therapy.

The research covered by this review clearly indicates that mRNA-encoded antibody technology is rapidly progressing from proof of concept toward a viable therapeutic platform. Their potential for emergency prophylaxis and treatment of viral infections is particularly high, especially for pathogens for which effective vaccines or direct-acting therapies are unavailable. The defining strength of this platform is its speed: within weeks, it is possible to generate a therapeutic agent capable of sustained in vivo expression of functional antibodies with native post-translational modifications.

Nevertheless, broad clinical application of mRNA-encoded antibodies is still substantially limited by several technological, manufacturing, and economic barriers. First, even though the platform is inherently versatile, the cost of producing mRNA that meets stringent GMP requirements remains high. The synthesis of highly purified mRNA templates, incorporation of modified nucleosides, co-transcriptional capping, and multistep chromatographic purification to remove immunogenic contaminants (e.g., double-stranded RNA) are all technically demanding and resource-intensive processes [347,348,349].

Second, logistics associated with the storage and distribution of ready-to-use therapeutics also represent a major constraint. To maintain their stability and prevent aggregation, most currently available LNP formulations and the mRNAs they encapsulate require cold-chain conditions, often at temperatures as low as −80 °C. This significantly restricts large-scale distribution, particularly in settings with limited infrastructure, where the need for effective prophylactic and therapeutic interventions is often greatest.

A third critical challenge lies in targeted delivery. Intravenous administration of LNPs is suitable for mRNA delivery predominantly to the liver and spleen, which is an advantage when aiming for systemic expression of secreted antibodies. However, achieving therapeutically relevant antibody concentrations in specific target organs remains difficult. This limitation is particularly evident for respiratory infections, where high local antibody levels in the lungs are required. Despite recent advances in selective delivery strategies, including SORT technology [147,150] and the PILOT and POST platforms [153,154], the development of LNPs capable of highly efficient and cell-type-specific mRNA delivery in vivo is still at an early stage. Moreover, LNPs may exhibit residual reactogenicity, inducing ADA that accelerate clearance of the expressed therapeutic antibodies and shorten the duration of their protective effect.

Finally, an additional fundamental limitation arises from the intrinsic properties of mRNA itself, namely, the transient nature of its expression. Although this makes the platform well-suited for short-term, emergency interventions, it restricts the duration of protection and poses challenges for applications requiring sustained antibody levels, such as chronic infections. Overcoming this constraint will likely require repeated dosing or the adoption of more complex approaches, including saRNA. However, these strategies are associated with additional technological and regulatory complexities.

It is essential to have a clear understanding of the mechanistic differences between mRNA vaccines and mRNA-encoded antibody therapeutics. mRNA vaccines function through active immunization: they encode viral antigens, such as the coronavirus spike (S) protein, that are expressed in antigen-presenting cells, particularly dendritic cells, thereby initiating adaptive immune responses and the formation of long-lived B cells and memory T cells [350]. Although this process unfolds over several weeks, it confers durable immunity. By contrast, mRNA-encoded antibodies represent a form of passive immunization. Here, a template sequence for the immediate production of neutralizing antibodies is delivered directly to the patient’s cells, bypassing the time-consuming process of developing adaptive immunity. Protective effects therefore appear within hours to days but are inherently transient, limited by both the duration of mRNA expression and the half-life of the synthesized antibody. Thus, rather than competing strategies, these approaches are complementary: vaccines are optimal for long-term population-level prophylaxis, whereas mRNA-encoded antibodies are well suited for emergency protection of vulnerable individuals, post-exposure prophylaxis, and therapeutic intervention.

One of the most distinctive and promising advantages of the mRNA platform is that it supports novel antibody formats, including membrane-anchored antibodies and intrabodies. The intracellular delivery of functional immunoglobulins or their variable fragments in protein form remains technically challenging. In contrast, mRNA delivery allows antibodies to be synthesized directly within the cytosol, where they can target and neutralize intracellular viral components, such as conserved proteins of the viral replication complex [230]. This capability introduces a new paradigm in antiviral therapy, as it enables targeting of viral proteins that are inaccessible to conventional secreted antibodies and are often less susceptible to escape mutations.

Future development of mRNA-encoded antibody technology is likely to focus on several key areas. First, optimization of delivery systems will be essential. This includes the design of more stable and less immunogenic lipids for LNPs, the development of biodegradable polymeric or hybrid nanoparticles that eliminate the need for ultra-low-temperature storage, and improved strategies for organ- and cell-specific targeting. In particular, optimizing formulations for inhalational or intranasal administration is expected to receive increasing attention because these routes allow high local antibody expression at mucosal surfaces—the primary portals of infection for many pathogens. Second, the therapeutic arsenal will be expanded, which will involve the development of combination mRNA cocktails encoding multiple antibodies with complementary mechanisms of action to overcome viral resistance, as well as the creation of bispecific and multispecific antibodies encoded by a single mRNA molecule. Third, personalized approaches represent a promising direction that can leverage the mRNA platform for rapid production of mRNA-based therapeutics encoding antibodies isolated from individual convalescent patients. Another promising approach is extending the duration of antibody expression to reduce the frequency of drug administration. This can be addressed by engineering mRNA constructs with modifications in untranslated regions or using self-amplifying replicons, as well as by optimizing antibody protein sequences, e.g., through mutations in the Fc fragment to increase the half-life. Finally, and critically important for translating laboratory advances into routine clinical practice, efforts will be made to streamline regulatory pathways, standardize manufacturing processes, and improve product stability to reduce costs and logistical barriers [97,348,349,351,352].

In conclusion, mRNA-encoded antibody technology represents a revolutionary breakthrough in biotechnology and antiviral therapy. Despite ongoing challenges related to manufacturing, logistics, and targeted delivery, its unique advantages—rapid development of new therapeutics, versatility, the ability to express complex antibody formats in vivo, and the potential to target intracellular pathogens—make it indispensable for combating current and future pandemic threats. Continued research and investment in mRNA antibody platforms are essential to transform this highly promising technology into a widely accessible and effective clinical tool.

## 8. Conclusions

Recent research suggests mRNA-encoded antibodies as a particularly promising platform for the prevention and treatment of viral infections. Optimized exogenous mRNA delivered in LNPs enables antibodies to be synthesized in vivo and, thus, bypasses many of the bottlenecks of traditional recombinant protein therapy, such as limited stability, labor-intensive and expensive manufacturing, and complex purification processes. Importantly, antibodies translated in vivo acquire native co-translational and post-translational modifications that are essential for their optimal biological activity.

Numerous preclinical studies across diverse viral infection models, such as HIV, hepatitis viruses, Ebola virus, smallpox, rabies, henipaviruses, arboviruses, and respiratory viruses, etc., have demonstrated high protective efficacy of mRNA-encoded antibodies. In some cases, a single dose fully prevented lethal infection and significantly reduced viral loads. The completion of Phase I clinical trials for *Moderna*’s mRNA-1944 therapeutic against chikungunya virus has become a milestone that confirmed the safety and practical feasibility of this technology in humans.

The mRNA-based platform supports a functionally diverse range of therapeutic agents. It includes not only conventional, secreted IgG antibodies, but also more advanced formats, such as intrabodies, membrane-anchored antibodies, chimeric scFv–Fc constructs, bispecific antibodies, IgA-isotype dimers, and IgM-like multimers. This flexibility enables targeted therapeutic strategies, including those aimed at neutralizing intracellular viral proteins that were previously inaccessible to antibody-based approaches. Local delivery is also feasible, and intranasal administration of mRNA–LNP complexes has been shown to achieve high antibody levels directly at the infection site. This approach helps improve protection against respiratory pathogens while reducing systemic doses and potential side effects.

Continued progress in targeted delivery, lower residual reactogenicity of LNPs, new mRNA cocktail formulations, and optimized expression profiles will further strengthen the potential of mRNA-encoded therapeutic antibodies as a powerful tool for emergency prevention and treatment of viral infections. In many scenarios, this technology could not only complement traditional approaches to passive immunization but also outperform them, especially during pandemics and outbreaks of newly emerging viruses.

## Data Availability

No new data were created or analyzed in this study. Data sharing is not applicable to this article.

## Abbreviations

The following abbreviations are used in this manuscript:

ADA	Anti-drug antibodies
ADARC	Aaron Diamond AIDS Research Center
ADE	Antibody-dependent enhancement
aIgA	GPI-anchored version of the same IgA
BHK	Baby Hamster Kidney
BLT	Bone Marrow-Liver-Thymus
bnAbs	Broadly neutralizing antibodies
CH	Constant domain of Heavy chains (CH1, CH2, CH3)
CHO	Chinese Hamster Ovary
circRNA	Circular RNA
CL	Constant domain of Light chains
DAF	Decay-accelerating factor
dIgA	IgA antibody dimers
DMG-PEG2k	1,2-dimyristoyl-rac-glycero-3-methoxy(polyethylene glycol)-2000
DOPE	1,2-Dioleoyl-sn-glycero-3-phosphatidylethanolamine
DOTAP	1,2-Dioleoyl-sn-glycero-3-trimethylammonium-propane
DSPC	1,2-Distearoyl-sn-glycero-3-phosphocholine
DSPE-PEG2k	1,2-Distearoyl-sn-glycero-3-phosphoethanolamine-N-carboxy(polyethylene glycol)-2000
EBOV	Ebola virus
EEV	Extracellular enveloped virion
EMCV	Encephalomyocarditis virus
EU	European Union
EUA	Emergency Use Authorization
Fab	Fragment antigen-binding
Fc	Fragment crystallizable
FcγRIV	Fcγ receptor IV
FDA	Food and Drug Administration
FP	Fusion peptide
G_4_S	poly-Glycine-Serine linker
GMII	Golgi α-mannosidase II
Gn	Glycoprotein N
GP	Glycoprotein
GPI	Glycosylphosphatidylinositol
hACE2	Human angiotensin-converting enzyme 2
HBsAg	Hepatitis B surface antigen
HBV	Hepatitis B virus
HC	Heavy Chain
HEK	Human Embryonic Kidney
HPV18	Human papillomavirus type 18
hFcRn	Human neonatal receptor for the Fc fragment of IgG
HIV	Human immunodeficiency virus
i.m.	intramuscular
i.n.	intranasal
i.p.	intraperitoneal
i.t.	intratracheal
i.v.	intravenous
ID	Identification Number
IFNAR	Interferon-α/β Receptor
IgA	Immunoglobulin A
IgG	Immunoglobulin G
IgM	Immunoglobulin M
IMV	Intracellular mature virion
IRES	Internal Ribosome Entry Site
JC	Joining chain
LA	M435L/N441A mutation
LC	Light Chain
LNPs	Lipid nanoparticles
LS	M428L/N434S mutation
M2e	Ectodomain of the influenza A virus M2 protein
MERS-CoV	Middle East respiratory syndrome coronavirus
MPXV	Mpox virus, or monkeypox virus
NA	Not Available
NIAID	National Institute of Allergy and Infectious Diseases
NSG mouse	NOD scid gamma mouse
nsP	Nonstructural protein
P2A peptide	Porcine Teschovirus-1 2A peptide
PBATE	Poly(butylene terephthalate-co-butylene adipate-co-ethylene terephthalate-co-ethylene adipate)
PEG	Polyethylene glycol
PRRs	Pattern recognition receptors
RBD	Receptor-binding domain
RIG-I	Retinoic acid-inducible gene 1
RSV	Respiratory Syncytial Virus
RTC	Replication-transcription complex
S protein	Surface protein
saRNA	Self-amplifying RNA
SARS-CoV-2	Severe acute respiratory syndrome coronavirus 2
SC	Single chain
SC-mRNA	Single-chain mRNA
scBCR-seq	Single B cell receptor sequencing
scFv	Single-chain Variable Fragment
scFv–Fc	Single-Chain Variable Fragment fused to the Fc region
scIgA	Single-chain IgA
SFTSV	Severe fever with thrombocytopenia syndrome virus
SHIV	Simian-Human Immunodeficiency Virus
sIgA	Secreted version of the same IgA
SUDV	Sudan virus
TLR	Toll-like receptors
TM	L234F/L235E/P331S triple mutation
UK	United Kingdom
US	United States
USAMRIID	United States Army Medical Research Institute of Infectious Diseases
UTRs	Untranslated regions
VACV	Vaccinia virus
VARV	Variola virus
VEEV	Venezuelan equine encephalitis virus
VH	Variable Heavy chain domain
VHH	Variable Heavy domain of Heavy chain (single-domain antibody)
VL	Variable Light chain domain
VRPs	Virus replicon particles
YTE	M252Y/S254T/T256E mutation

## References

[1] Jin X., Ren J., Li R., Gao Y., Zhang H., Li J., Zhang J., Wang X., Wang G. (2021). Global burden of upper respiratory infections in 204 countries and territories, from 1990 to 2019. eClinicalMedicine.

[2] Roth G.A., Picece V.C.T.M., Ou B.S., Luo W., Pulendran B., Appel E.A. (2022). Designing spatial and temporal control of vaccine responses. Nat. Rev. Mater..

[3] Mao T., Kim J., Peña-Hernández M.A., Valle G., Moriyama M., Luyten S., Ott I.M., Gomez-Calvo M.L., Gehlhausen J.R., Baker E. (2024). Intranasal neomycin evokes broad-spectrum antiviral immunity in the upper respiratory tract. Proc. Natl. Acad. Sci. USA.

[4] Law G.L., Korth M.J., Benecke A.G., Katze M.G. (2013). Systems virology: Host-directed approaches to viral pathogenesis and drug targeting. Nat. Rev. Microbiol..

[5] Casadevall A., Dadachova E., Pirofski L.A. (2004). Passive antibody therapy for infectious diseases. Nat. Rev. Microbiol..

[6] Hale G. (2006). Therapeutic antibodies--delivering the promise?. Adv. Drug Deliv. Rev..

[7] Buss N.A., Henderson S.J., McFarlane M., Shenton J.M., de Haan L. (2012). Monoclonal antibody therapeutics: History and future. Curr. Opin. Pharmacol..

[8] Tsumoto K., Isozaki Y., Yagami H., Tomita M. (2019). Future perspectives of therapeutic monoclonal antibodies. Immunotherapy.

[9] Crowe J.E. (2022). Human Antibodies for Viral Infections. Annu. Rev. Immunol..

[10] Yuseff M.I., Pierobon P., Reversat A., Lennon-Duménil A.M. (2013). How B cells capture, process and present antigens: A crucial role for cell polarity. Nat. Rev. Immunol..

[11] Tonegawa S. (1983). Somatic generation of antibody diversity. Nature.

[12] Upasani V., Rodenhuis-Zybert I., Cantaert T. (2021). Antibody-independent functions of B cells during viral infections. PLoS Pathog..

[13] Klasse P.J. (2014). Neutralization of Virus Infectivity by Antibodies: Old Problems in New Perspectives. Adv. Biol..

[14] Kim S.J., Park Y., Hong H.J. (2005). Antibody engineering for the development of therapeutic antibodies. Mol. Cells.

[15] Nelson A.L., Dhimolea E., Reichert J.M. (2010). Development trends for human monoclonal antibody therapeutics. Nat. Rev. Drug Discov..

[16] Bradbury A.R., Sidhu S., Dübel S., McCafferty J. (2011). Beyond natural antibodies: The power of in vitro display technologies. Nat. Biotechnol..

[17] Both L., Banyard A.C., van Dolleweerd C., Wright E., Ma J.K., Fooks A.R. (2013). Monoclonal antibodies for prophylactic and therapeutic use against viral infections. Vaccine.

[18] Lanzavecchia A., Frühwirth A., Perez L., Corti D. (2016). Antibody-guided vaccine design: Identification of protective epitopes. Curr. Opin. Immunol..

[19] Aboul-Ella H., Gohar A., Ali A.A., Ismail L.M., Mahmoud A.E.E., Elkhatib W.F., Aboul-Ella H. (2024). Monoclonal antibodies: From magic bullet to precision weapon. Mol. Biomed..

[20] Mekala J.R., Nalluri H.P., Reddy P.N., Sainath S.B., Kumar S.N.S., Kiran S.G.V.S.D., Dhiman R., Chamarthy S., Komaragiri R.R., Manyam R.R. (2024). Emerging trends and therapeutic applications of monoclonal antibodies. Gene.

[21] Chan A.C., Martyn G.D., Carter P.J. (2025). Fifty years of monoclonals: The past, present and future of antibody therapeutics. Nat. Rev. Immunol..

[22] Lu R.M., Chiang H.L., Yuan J.P., Wang H.H., Chen C.Y., Panda S.S., Liang K.H., Peng H.P., Ko S.H., Hsu H.J. (2025). Technological advancements in antibody-based therapeutics for treatment of diseases. J. Biomed. Sci..

[23] Castelli M.S., McGonigle P., Hornby P.J. (2019). The pharmacology and therapeutic applications of monoclonal antibodies. Pharmacol. Res. Perspect..

[24] Goulet D.R., Atkins W.M. (2020). Considerations for the Design of Antibody-Based Therapeutics. J. Pharm. Sci..

[25] Hansel T.T., Kropshofer H., Singer T., Mitchell J.A., George A.J. (2010). The safety and side effects of monoclonal antibodies. Nat. Rev. Drug Discov..

[26] Tiller K.E., Tessier P.M. (2015). Advances in Antibody Design. Annu. Rev. Biomed. Eng..

[27] Wang X., Mathieu M., Brezski R.J. (2018). IgG Fc engineering to modulate antibody effector functions. Protein Cell.

[28] Saunders K.O. (2019). Conceptual Approaches to Modulating Antibody Effector Functions and Circulation Half-Life. Front. Immunol..

[29] Vincent K.J., Zurini M. (2012). Current strategies in antibody engineering: Fc engineering and pH-dependent antigen binding, bispecific antibodies and antibody drug conjugates. Biotechnol. J..

[30] Stone C.A., Spiller B.W., Smith S.A. (2024). Engineering therapeutic monoclonal antibodies. J. Allergy Clin. Immunol..

[31] Keri D., Walker M., Singh I., Nishikawa K., Garces F. (2023). Next generation of multispecific antibody engineering. Antib. Ther..

[32] Bates A., Power C.A. (2019). David vs. Goliath: The Structure, Function, and Clinical Prospects of Antibody Fragments. Antibodies.

[33] Strohl W.R. (2025). Structure and function of therapeutic antibodies approved by the US FDA in 2024. Antib. Ther..

[34] US Food and Drug Administration Drugs@FDA: FDA-Approved Drugs. https://www.fda.gov/drugs/novel-drug-approvals-fda/novel-drug-approvals-2025.

[35] Tang A., Chen Z., Cox K.S., Su H.P., Callahan C., Fridman A., Zhang L., Patel S.B., Cejas P.J., Swoyer R. (2019). A potent broadly neutralizing human RSV antibody targets conserved site IV of the fusion glycoprotein. Nat. Commun..

[36] Feige M.J., Hendershot L.M., Buchner J. (2010). How antibodies fold. Trends Biochem. Sci..

[37] European Commission Public Health—Union Register of Medicinal Products. https://ec.europa.eu/health/documents/community-register/html/index_en.htm.

[38] The Antibody Society Therapeutic Monoclonal Antibodies Approved or in Regulatory Review. https://www.antibodysociety.org/antibody-therapeutics-product-data/.

[39] AdisInsight Database by Springer Nature. https://adisinsight.springer.com/.

[40] ClinicalTrials.gov, The U.S. National Institutes of Health (NIH) Registry and Results Database of Clinical Trials https://clinicaltrials.gov/.

[41] Antibodies to Watch. mAbs. https://www.tandfonline.com/journals/kmab20/collections/antibodies-to-watch.

[42] Mokhtary P., Pourhashem Z., Mehrizi A.A., Sala C., Rappuoli R. (2022). Recent Progress in the Discovery and Development of Monoclonal Antibodies against Viral Infections. Biomedicines.

[43] Akinosoglou K., Rigopoulos E.A., Kaiafa G., Daios S., Karlafti E., Ztriva E., Polychronopoulos G., Gogos C., Savopoulos C. (2022). Tixagevimab/Cilgavimab in SARS-CoV-2 Prophylaxis and Therapy: A Comprehensive Review of Clinical Experience. Viruses.

[44] Sivapalasingam S., Kamal M., Slim R., Hosain R., Shao W., Stoltz R., Yen J., Pologe L.G., Cao Y., Partridge M. (2018). Safety, pharmacokinetics, and immunogenicity of a co-formulated cocktail of three human monoclonal antibodies targeting Ebola virus glycoprotein in healthy adults: A randomised, first-in-human phase 1 study. Lancet Infect. Dis..

[45] Liu X., Li J., Zha Y., Wang Z., Jiang Y., Zhang X., Guo J., Yu J., Li X., Zhang Q. (2025). The efficacy and safety of SYN023 (Zamerovimab and Mazorelvimab injection), the recombinant humanized anti-rabies virus monoclonal antibody mixture, combined with rabies vaccine in a WHO category III rabies post-exposure population: A randomized, double-blind, positive control, phase III clinical trial. Vaccine.

[46] Johnson S., Oliver C., Prince G.A., Hemming V.G., Pfarr D.S., Wang S.C., Dormitzer M., O’Grady J., Koenig S., Tamura J.K. (1997). Development of a humanized monoclonal antibody (MEDI-493) with potent in vitro and in vivo activity against respiratory syncytial virus. J. Infect. Dis..

[47] The IMpact-RSV Study Group (1998). Palivizumab, a humanized respiratory syncytial virus monoclonal antibody, reduces hospitalization from respiratory syncytial virus infection in high-risk infants. Pediatrics.

[48] Griffin M.P., Khan A.A., Esser M.T., Jensen K., Takas T., Kankam M.K., Villafana T., Dubovsky F. (2017). Safety, Tolerability, and Pharmacokinetics of MEDI8897, the Respiratory Syncytial Virus Prefusion F-Targeting Monoclonal Antibody with an Extended Half-Life, in Healthy Adults. Antimicrob. Agents Chemother..

[49] Domachowske J.B., Khan A.A., Esser M.T., Jensen K., Takas T., Villafana T., Dubovsky F., Griffin M.P. (2018). Safety, Tolerability and Pharmacokinetics of MEDI8897, an Extended Half-life Single-dose Respiratory Syncytial Virus Prefusion F-targeting Monoclonal Antibody Administered as a Single Dose to Healthy Preterm Infants. Pediatr. Infect. Dis. J..

[50] Hammitt L.L., Dagan R., Yuan Y., Baca Cots M., Bosheva M., Madhi S.A., Muller W.J., Zar H.J., Brooks D., Grenham A. (2022). Nirsevimab for Prevention of RSV in Healthy Late-Preterm and Term Infants. N. Engl. J. Med..

[51] Aliprantis A.O., Wolford D., Caro L., Maas B.M., Ma H., Montgomery D.L., Sterling L.M., Hunt A., Cox K.S., Vora K.A. (2021). A Phase 1 Randomized, Double-Blind, Placebo-Controlled Trial to Assess the Safety, Tolerability, and Pharmacokinetics of a Respiratory Syncytial Virus Neutralizing Monoclonal Antibody MK-1654 in Healthy Adults. Clin. Pharmacol. Drug Dev..

[52] Phuah J.Y., Maas B.M., Tang A., Zhang Y., Caro L., Railkar R.A., Swanson M.D., Cao Y., Li H., Roadcap B. (2023). Quantification of clesrovimab, an investigational, half-life extended, anti-respiratory syncytial virus protein F human monoclonal antibody in the nasal epithelial lining fluid of healthy adults. Biomed. Pharmacother..

[53] Madhi S.A., Simões E.A.F., Acevedo A., Novoa Pizarro J.M., Shepard J.S., Railkar R.A., Cao X., Maas B.M., Zang X., Krick A. (2025). A Phase 1b/2a Trial of a Half-life Extended Respiratory Syncytial Virus Neutralizing Antibody, Clesrovimab, in Healthy Preterm and Full-term Infants. J. Infect. Dis..

[54] Farber H.J., Buckwold F.J., Lachman B., Simpson J.S., Buck E., Arun M., Valadez A.M., Ruiz T., Alonzo J., Henry A. (2016). Observed Effectiveness of Palivizumab for 29-36-Week Gestation Infants. Pediatrics.

[55] Esposito S., Abu Raya B., Baraldi E., Flanagan K., Martinon Torres F., Tsolia M., Zielen S. (2022). RSV Prevention in All Infants: Which Is the Most Preferable Strategy?. Front. Immunol..

[56] Birch J.R., Racher A.J. (2006). Antibody production. Adv. Drug Deliv. Rev..

[57] Wang W., Wang E.Q., Balthasar J.P. (2008). Monoclonal antibody pharmacokinetics and pharmacodynamics. Clin. Pharmacol. Ther..

[58] Kelley B. (2009). Industrialization of mAb production technology: The bioprocessing industry at a crossroads. MAbs.

[59] Arosio P., Barolo G., Müller-Späth T., Wu H., Morbidelli M. (2011). Aggregation stability of a monoclonal antibody during downstream processing. Pharm. Res..

[60] Kunert R., Reinhart D. (2016). Advances in recombinant antibody manufacturing. Appl. Microbiol. Biotechnol..

[61] Sifniotis V., Cruz E., Eroglu B., Kayser V. (2019). Current Advancements in Addressing Key Challenges of Therapeutic Antibody Design, Manufacture, and Formulation. Antibodies.

[62] Schlake T., Thess A., Thran M., Jordan I. (2019). mRNA as novel technology for passive immunotherapy. Cell Mol. Life Sci..

[63] Van Hoecke L., Roose K. (2019). How mRNA therapeutics are entering the monoclonal antibody field. J. Transl. Med..

[64] Schlake T., Thran M., Fiedler K., Heidenreich R., Petsch B., Fotin-Mleczek M. (2019). mRNA: A Novel Avenue to Antibody Therapy?. Mol. Ther..

[65] Deal C.E., Carfi A., Plante O.J. (2021). Advancements in mRNA Encoded Antibodies for Passive Immunotherapy. Vaccines.

[66] Reimann K.A., Burkly L.C., Burrus B., Waite B.C., Lord C.I., Letvin N.L. (1993). In vivo administration to rhesus monkeys of a CD4-specific monoclonal antibody capable of blocking AIDS virus replication. AIDS Res. Hum. Retroviruses.

[67] Pascal K.E., Dudgeon D., Trefry J.C., Anantpadma M., Sakurai Y., Murin C.D., Turner H.L., Fairhurst J., Torres M., Rafique A. (2018). Development of Clinical-Stage Human Monoclonal Antibodies That Treat Advanced Ebola Virus Disease in Nonhuman Primates. J. Infect. Dis..

[68] Corti D., Misasi J., Mulangu S., Stanley D.A., Kanekiyo M., Wollen S., Ploquin A., Doria-Rose N.A., Staupe R.P., Bailey M. (2016). Protective monotherapy against lethal Ebola virus infection by a potently neutralizing antibody. Science.

[69] Sloan S.E., Hanlon C., Weldon W., Niezgoda M., Blanton J., Self J., Rowley K.J., Mandell R.B., Babcock G.J., Thomas W.D. (2007). Identification and characterization of a human monoclonal antibody that potently neutralizes a broad panel of rabies virus isolates. Vaccine.

[70] Müller T., Dietzschold B., Ertl H., Fooks A.R., Freuling C., Fehlner-Gardiner C., Kliemt J., Meslin F.X., Franka R., Rupprecht C.E. (2009). Development of a mouse monoclonal antibody cocktail for post-exposure rabies prophylaxis in humans. PLoS Negl. Trop. Dis..

[71] Wang M.X., Jia M., Jin M., Han J., Duan J., Wang L.Q., Jin R.H., Li N., Yao J.L., Li Y.F. (2013). Safety of a single injection with recombinant human rabies immunoglobin at various dosages in humans. Chin. J. Biol..

[72] Chao T.Y., Ren S., Shen E., Moore S., Zhang S.F., Chen L., Rupprecht C.E., Tsao E. (2017). SYN023, a novel humanized monoclonal antibody cocktail, for post-exposure prophylaxis of rabies. PLoS Negl. Trop. Dis..

[73] Sun S., Zhao G., Liu C., Fan W., Zhou X., Zeng L., Guo Y., Kou Z., Yu H., Li J. (2015). Treatment with anti-C5a antibody improves the outcome of H7N9 virus infection in African green monkeys. Clin. Infect. Dis..

[74] Rappazzo C.G., Tse L.V., Kaku C.I., Wrapp D., Sakharkar M., Huang D., Deveau L.M., Yockachonis T.J., Herbert A.S., Battles M.B. (2021). Broad and potent activity against SARS-like viruses by an engineered human monoclonal antibody. Science.

[75] Nutalai R., Zhou D., Tuekprakhon A., Ginn H.M., Supasa P., Liu C., Huo J., Mentzer A.J., Duyvesteyn H.M.E., Dijokaite-Guraliuc A. (2022). Potent cross-reactive antibodies following Omicron breakthrough in vaccinees. Cell.

[76] Wang C.Y., Sawyer L.S., Murthy K.K., Fang X., Walfield A.M., Ye J., Wang J.J., Chen P.D., Li M.L., Salas M.T. (1999). Postexposure immunoprophylaxis of primary isolates by an antibody to HIV receptor complex. Proc. Natl. Acad. Sci. USA.

[77] Scheid J.F., Mouquet H., Ueberheide B., Diskin R., Klein F., Oliveira T.Y., Pietzsch J., Fenyo D., Abadir A., Velinzon K. (2011). Sequence and structural convergence of broad and potent HIV antibodies that mimic CD4 binding. Science.

[78] Mouquet H., Scharf L., Euler Z., Liu Y., Eden C., Scheid J.F., Halper-Stromberg A., Gnanapragasam P.N., Spencer D.I., Seaman M.S. (2012). Complex-type N-glycan recognition by potent broadly neutralizing HIV antibodies. Proc. Natl. Acad. Sci. USA.

[79] Huang J., Kang B.H., Ishida E., Zhou T., Griesman T., Sheng Z., Wu F., Doria-Rose N.A., Zhang B., McKee K. (2016). Identification of a CD4-Binding-Site Antibody to HIV that Evolved Near-Pan Neutralization Breadth. Immunity.

[80] Rudicell R.S., Kwon Y.D., Ko S.Y., Pegu A., Louder M.K., Georgiev I.S., Wu X., Zhu J., Boyington J.C., Chen X. (2014). Enhanced potency of a broadly neutralizing HIV-1 antibody in vitro improves protection against lentiviral infection in vivo. J. Virol..

[81] Moore P.L., Gray E.S., Sheward D., Madiga M., Ranchobe N., Lai Z., Honnen W.J., Nonyane M., Tumba N., Hermanus T. (2011). Potent and broad neutralization of HIV-1 subtype C by plasma antibodies targeting a quaternary epitope including residues in the V2 loop. J. Virol..

[82] Iacob S.A., Iacob D.G. (2017). Ibalizumab Targeting CD4 Receptors, An Emerging Molecule in HIV Therapy. Front. Microbiol..

[83] Walker L.M., Huber M., Doores K.J., Falkowska E., Pejchal R., Julien J.P., Wang S.K., Ramos A., Chan-Hui P.Y., Moyle M. (2011). Broad neutralization coverage of HIV by multiple highly potent antibodies. Nature.

[84] Sok D., van Gils M.J., Pauthner M., Julien J.P., Saye-Francisco K.L., Hsueh J., Briney B., Lee J.H., Le K.M., Lee P.S. (2014). Recombinant HIV envelope trimer selects for quaternary-dependent antibodies targeting the trimer apex. Proc. Natl. Acad. Sci. USA.

[85] Lempp F.A., Volz T., Cameroni E., Benigni F., Zhou J., Rosen L.E., Noack J., Zatta F., Kaiser H., Bianchi S. (2023). Potent broadly neutralizing antibody VIR-3434 controls hepatitis B and D virus infection and reduces HBsAg in humanized mice. J. Hepatol..

[86] Long C., Wang W., Hao X., Yu C., Feng Y., Tu C., Sun S., Bian L., Liu Z., Wang L. (2023). Development of a novel bispecific antibody GR1801 for rabies. J. Med. Virol..

[87] Fenwick C., Turelli P., Ni D., Perez L., Lau K., Herate C., Marlin R., Lana E., Pellaton C., Raclot C. (2022). Patient-derived monoclonal antibody neutralizes SARS-CoV-2 Omicron variants and confers full protection in monkeys. Nat. Microbiol..

[88] Cao Y., Jian F., Zhang Z., Yisimayi A., Hao X., Bao L., Yuan F., Yu Y., Du S., Wang J. (2022). Rational identification of potent and broad sarbecovirus-neutralizing antibody cocktails from SARS convalescents. Cell Rep..

[89] Bhatti T. (2025). Global Genomic Surveillance Reveals Pre-EUA Fixation of Pemivibart (VYD2311) Escape Constellations in SARS-CoV-2. Preprints.org.

[90] Tavernier G., Andries O., Demeester J., Sanders N.N., De Smedt S.C., Rejman J. (2011). mRNA as gene therapeutic: How to control protein expression. J. Control. Release.

[91] Rohner E., Yang R., Foo K.S., Goedel A., Chien K.R. (2022). Unlocking the promise of mRNA therapeutics. Nat. Biotechnol..

[92] Wang Y.S., Kumari M., Chen G.H., Hong M.H., Yuan J.P., Tsai J.L., Wu H.C. (2023). mRNA-based vaccines and therapeutics: An in-depth survey of current and upcoming clinical applications. J. Biomed. Sci..

[93] Wei H.H., Zheng L., Wang Z. (2023). mRNA therapeutics: New vaccination and beyond. Fundam. Res..

[94] Parhiz H., Atochina-Vasserman E.N., Weissman D. (2024). mRNA-based therapeutics: Looking beyond COVID-19 vaccines. Lancet.

[95] Shi Y., Shi M., Wang Y., You J. (2024). Progress and prospects of mRNA-based drugs in pre-clinical and clinical applications. Signal Transduct. Target. Ther..

[96] Pardi N., Krammer F. (2024). mRNA vaccines for infectious diseases—Advances, challenges and opportunities. Nat. Rev. Drug Discov..

[97] Żak M.M., Zangi L. (2025). Clinical development of therapeutic mRNA applications. Mol. Ther..

[98] Yu K., Xiao G., Chen B., Xu N., Liu W. (2025). Nucleic acid-encoded antibody gene transfer-next generation of antibody therapies. Drug Deliv..

[99] Zhou H., Wei D., Chen Z., Chen H., Dong C., Yao W., Wang J., Liu X., Li Y., Yang Y. (2026). The mRNA-Based Innovative Strategy: Progress and Challenges. Nanomicro Lett..

[100] Sahin U., Karikó K., Türeci Ö. (2014). mRNA-based therapeutics--developing a new class of drugs. Nat. Rev. Drug Discov..

[101] Weng Y., Li C., Yang T., Hu B., Zhang M., Guo S., Xiao H., Liang X.J., Huang Y. (2020). The challenge and prospect of mRNA therapeutics landscape. Biotechnol. Adv..

[102] Dahm G.C., Pieper M., Schepers H., Rentmeister A. (2026). The 5′ Cap Epitranscriptome and Beyond: Natural and Engineered 5′ Cap Modifications for Optimizing mRNA Therapeutics and Functional Studies. ChemMedChem.

[103] Chen H., Liu D., Guo J., Aditham A., Zhou Y., Tian J., Luo S., Ren J., Hsu A., Huang J. (2025). Branched chemically modified poly(A) tails enhance the translation capacity of mRNA. Nat. Biotechnol..

[104] Chen H., Qin W., Bao H., Chen X., Ye X., Wang Y., Liu L., Zhang Y., Sun Y., Zhang T. (2025). Poly(A) variants supporting robust transmission stability in bacteria and high protein expression in animals for mRNA in vitro transcription. Mol. Ther. Nucleic Acids.

[105] Spiewla T., Czubak K., Pilch Z., Baranowski M.R., Krawczyk P.S., Affek K., Antczak W., Szulc-Gasiorowska M., Chmielinski S., Mroczek S. (2026). PolyA tail segmentation improves the stability of the template DNA and increases the translatability of in vitro transcribed mRNA. Nucleic Acids Res..

[106] Hou X., Shi J., Xiao Y. (2024). mRNA medicine: Recent progresses in chemical modification, design, and engineering. Nano Res..

[107] Zhang Z., Ong Y.H., Yang B., Fan B., Yang Y.Y., Ni Q. (2026). Chemical engineering strategies to enhance mRNA-LNP stability for therapeutic applications. Biomater. Sci..

[108] Holtkamp S., Kreiter S., Selmi A., Simon P., Koslowski M., Huber C., Türeci O., Sahin U. (2006). Modification of antigen-encoding RNA increases stability, translational efficacy, and T-cell stimulatory capacity of dendritic cells. Blood.

[109] Mauger D.M., Cabral B.J., Presnyak V., Su S.V., Reid D.W., Goodman B., Link K., Khatwani N., Reynders J., Moore M.J. (2019). mRNA structure regulates protein expression through changes in functional half-life. Proc. Natl. Acad. Sci. USA.

[110] Zhang H., Zhang L., Lin A., Xu C., Li Z., Liu K., Liu B., Ma X., Zhao F., Jiang H. (2023). Algorithm for optimized mRNA design improves stability and immunogenicity. Nature.

[111] Orlandini von Niessen A.G., Poleganov M.A., Rechner C., Plaschke A., Kranz L.M., Fesser S., Diken M., Löwer M., Vallazza B., Beissert T. (2019). Improving mRNA-Based Therapeutic Gene Delivery by Expression-Augmenting 3′ UTRs Identified by Cellular Library Screening. Mol. Ther..

[112] Mamaghani S., Penna R.R., Frei J., Wyss C., Mellett M., Look T., Weiss T., Guenova E., Kündig T.M., Lauchli S. (2024). Synthetic mRNAs Containing Minimalistic Untranslated Regions Are Highly Functional In Vitro and In Vivo. Cells.

[113] Leppek K., Das R., Barna M. (2018). Functional 5′ UTR mRNA structures in eukaryotic translation regulation and how to find them. Nat. Rev. Mol. Cell Biol..

[114] Leppek K., Byeon G.W., Kladwang W., Wayment-Steele H.K., Kerr C.H., Xu A.F., Kim D.S., Topkar V.V., Choe C., Rothschild D. (2022). Combinatorial optimization of mRNA structure, stability, and translation for RNA-based therapeutics. Nat. Commun..

[115] Nelson J., Sorensen E.W., Mintri S., Rabideau A.E., Zheng W., Besin G., Khatwani N., Su S.V., Miracco E.J., Issa W.J. (2020). Impact of mRNA chemistry and manufacturing process on innate immune activation. Sci. Adv..

[116] Verbeke R., Hogan M.J., Loré K., Pardi N. (2022). Innate immune mechanisms of mRNA vaccines. Immunity.

[117] Karikó K., Buckstein M., Ni H., Weissman D. (2005). Suppression of RNA recognition by Toll-like receptors: The impact of nucleoside modification and the evolutionary origin of RNA. Immunity.

[118] Karikó K., Muramatsu H., Welsh F.A., Ludwig J., Kato H., Akira S., Weissman D. (2008). Incorporation of pseudouridine into mRNA yields superior nonimmunogenic vector with increased translational capacity and biological stability. Mol. Ther..

[119] Anderson B.R., Muramatsu H., Nallagatla S.R., Bevilacqua P.C., Sansing L.H., Weissman D., Karikó K. (2010). Incorporation of pseudouridine into mRNA enhances translation by diminishing PKR activation. Nucleic Acids Res..

[120] Kormann M.S., Hasenpusch G., Aneja M.K., Nica G., Flemmer A.W., Herber-Jonat S., Huppmann M., Mays L.E., Illenyi M., Schams A. (2011). Expression of therapeutic proteins after delivery of chemically modified mRNA in mice. Nat. Biotechnol..

[121] Weissman D. (2015). mRNA transcript therapy. Expert. Rev. Vaccines.

[122] Kauffman K.J., Mir F.F., Jhunjhunwala S., Kaczmarek J.C., Hurtado J.E., Yang J.H., Webber M.J., Kowalski P.S., Heartlein M.W., DeRosa F. (2016). Efficacy and immunogenicity of unmodified and pseudouridine-modified mRNA delivered systemically with lipid nanoparticles in vivo. Biomaterials.

[123] Qin S., Tang X., Chen Y., Chen K., Fan N., Xiao W., Zheng Q., Li G., Teng Y., Wu M. (2022). mRNA-based therapeutics: Powerful and versatile tools to combat diseases. Signal Transduct. Target. Ther..

[124] Zhou F., Huang L., Li S., Yang W., Chen F., Cai Z., Liu X., Xu W., Lehto V.P., Lächelt U. (2023). From structural design to delivery: mRNA therapeutics for cancer immunotherapy. Exploration.

[125] Karikó K., Weissman D. (2007). Naturally occurring nucleoside modifications suppress the immunostimulatory activity of RNA: Implication for therapeutic RNA development. Curr. Opin. Drug Discov. Dev..

[126] Baden L.R., El Sahly H.M., Essink B., Kotloff K., Frey S., Novak R., Diemert D., Spector S.A., Rouphael N., Creech C.B. (2021). Efficacy and Safety of the mRNA-1273 SARS-CoV-2 Vaccine. N. Engl. J. Med..

[127] Polack F.P., Thomas S.J., Kitchin N., Absalon J., Gurtman A., Lockhart S., Perez J.L., Pérez Marc G., Moreira E.D., Zerbini C. (2020). Safety and Efficacy of the BNT162b2 mRNA COVID-19 Vaccine. N. Engl. J. Med..

[128] Karikó K., Ni H., Capodici J., Lamphier M., Weissman D. (2004). mRNA is an endogenous ligand for Toll-like receptor 3. J. Biol. Chem..

[129] Karikó K., Muramatsu H., Ludwig J., Weissman D. (2011). Generating the optimal mRNA for therapy: HPLC purification eliminates immune activation and improves translation of nucleoside-modified, protein-encoding mRNA. Nucleic Acids Res..

[130] Baiersdörfer M., Boros G., Muramatsu H., Mahiny A., Vlatkovic I., Sahin U., Karikó K. (2019). A Facile Method for the Removal of dsRNA Contaminant from In Vitro-Transcribed mRNA. Mol. Ther. Nucleic Acids.

[131] Desai D.A., Cristofoletti R. (2025). Bench to Bedside Modeling of mRNA Encoding IgG Using a Multiscale Mechanistic Pharmacokinetic-Toxicokinetic (PK-TK) Model: A Case Study with Anti-Claudin 18.2. Clin. Transl. Sci..

[132] Lorenz C., Fotin-Mleczek M., Roth G., Becker C., Dam T.C., Verdurmen W.P., Brock R., Probst J., Schlake T. (2011). Protein expression from exogenous mRNA: Uptake by receptor-mediated endocytosis and trafficking via the lysosomal pathway. RNA Biol..

[133] Kowalski P.S., Rudra A., Miao L., Anderson D.G. (2019). Delivering the Messenger: Advances in Technologies for Therapeutic mRNA Delivery. Mol. Ther..

[134] Li B., Zhang X., Dong Y. (2019). Nanoscale platforms for messenger RNA delivery. Wiley Interdiscip. Rev. Nanomed. Nanobiotechnol..

[135] Guan S., Rosenecker J. (2017). Nanotechnologies in delivery of mRNA therapeutics using nonviral vector-based delivery systems. Gene Ther..

[136] Li D.F., Liu Q.S., Yang M.F., Xu H.M., Zhu M.Z., Zhang Y., Xu J., Tian C.M., Yao J., Wang L.S. (2023). Nanomaterials for mRNA-based therapeutics: Challenges and opportunities. Bioeng. Transl. Med..

[137] Prinindya K.N.N., Ashraf G.M., Uddin S., Mahdi E., Hasan A. (2025). Recent advances in engineering approaches for delivery of mRNA-based therapeutics. Biomater. Adv..

[138] Pardi N., Tuyishime S., Muramatsu H., Kariko K., Mui B.L., Tam Y.K., Madden T.D., Hope M.J., Weissman D. (2015). Expression kinetics of nucleoside-modified mRNA delivered in lipid nanoparticles to mice by various routes. J. Control. Release.

[139] Kauffman K.J., Dorkin J.R., Yang J.H., Heartlein M.W., DeRosa F., Mir F.F., Fenton O.S., Anderson D.G. (2015). Optimization of Lipid Nanoparticle Formulations for mRNA Delivery In Vivo with Fractional Factorial and Definitive Screening Designs. Nano Lett..

[140] Hou X., Zaks T., Langer R., Dong Y. (2021). Lipid nanoparticles for mRNA delivery. Nat. Rev. Mater..

[141] Jeong M., Lee Y., Park J., Jung H., Lee H. (2023). Lipid nanoparticles (LNPs) for in vivo RNA delivery and their breakthrough technology for future applications. Adv. Drug Deliv. Rev..

[142] Vasileva O., Zaborova O., Shmykov B., Ivanov R., Reshetnikov V. (2024). Composition of lipid nanoparticles for targeted delivery: Application to mRNA therapeutics. Front. Pharmacol..

[143] Haque M.A., Shrestha A., Mikelis C.M., Mattheolabakis G. (2024). Comprehensive analysis of lipid nanoparticle formulation and preparation for RNA delivery. Int. J. Pharm. X.

[144] Li X., Qi J., Wang J., Hu W., Zhou W., Wang Y., Li T. (2024). Nanoparticle technology for mRNA: Delivery strategy, clinical application and developmental landscape. Theranostics.

[145] Lee Y., Guo K., Oh M., Kim E., Jeong Y., Yoon Y., Choi Y., Hwang Y., Byeon Y., Dong Y. (2025). Advances in the rational design of ionizable lipids for mRNA therapeutics. Mat. Today.

[146] Kulkarni J.A., Witzigmann D., Chen S., Cullis P.R., van der Meel R. (2019). Lipid Nanoparticle Technology for Clinical Translation of siRNA Therapeutics. Acc. Chem. Res..

[147] Cheng Q., Wei T., Farbiak L., Johnson L.T., Dilliard S.A., Siegwart D.J. (2020). Selective organ targeting (SORT) nanoparticles for tissue-specific mRNA delivery and CRISPR-Cas gene editing. Nat. Nanotechnol..

[148] Liu S., Cheng Q., Wei T., Yu X., Johnson L.T., Farbiak L., Siegwart D.J. (2021). Membrane-destabilizing ionizable phospholipids for organ-selective mRNA delivery and CRISPR-Cas gene editing. Nat. Mater..

[149] Dilliard S.A., Cheng Q., Siegwart D.J. (2021). On the mechanism of tissue-specific mRNA delivery by selective organ targeting nanoparticles. Proc. Natl. Acad. Sci. USA.

[150] Wang X., Liu S., Sun Y., Yu X., Lee S.M., Cheng Q., Wei T., Gong J., Robinson J., Zhang D. (2023). Preparation of selective organ-targeting (SORT) lipid nanoparticles (LNPs) using multiple technical methods for tissue-specific mRNA delivery. Nat. Protoc..

[151] Zwolsman R., Darwish Y.B., Kluza E., van der Meel R. (2025). Engineering Lipid Nanoparticles for mRNA Immunotherapy. Wiley Interdiscip. Rev. Nanomed. Nanobiotechnol..

[152] Xue L., Zhao G., Gong N., Han X., Shepherd S.J., Xiong X., Xiao Z., Palanki R., Xu J., Swingle K.L. (2025). Combinatorial design of siloxane-incorporated lipid nanoparticles augments intracellular processing for tissue-specific mRNA therapeutic delivery. Nat. Nanotechnol..

[153] Lin Y., Li M., Luo Z., Meng Y., Zong Y., Ren H., Yu X., Tan X., Liu F., Wei T. (2026). Tissue-specific mRNA delivery and prime editing with peptide-ionizable lipid nanoparticles. Nat. Mater..

[154] Chang T., Zheng Y., Jiang M., Jia S., Bai J., Zheng Z., Li S., Guo J., Wang Y., Wang Y. (2026). Peptide codes for organ-selective mRNA delivery. Nat. Mater..

[155] Dietmair B., Humphries J., Mercer T.R., Thurecht K.J., Howard C.B., Cheetham S.W. (2025). Targeted mRNA delivery with bispecific antibodies that tether LNPs to cell surface markers. Mol. Ther. Nucleic Acids.

[156] Chen M.Z., Yuen D., McLeod V.M., Yong K.W., Smyth C.H., Herling B.R., Payne T.J., Fabb S.A., Belousoff M.J., Algarni A. (2025). A versatile antibody capture system drives specific in vivo delivery of mRNA-loaded lipid nanoparticles. Nat. Nanotechnol..

[157] Chen L., Van Der Weken H., Zwaenepoel O., Okkelman I.A., Aelvoet J., Van Denberghe E., Gettemans J., Dmitriev R.I., De Geest B.G., Cox E. (2025). Cell-specific mRNA delivery via nanobody-functionalized lipid nanoparticles. J. Control. Release.

[158] He X., Li G., Huang L., Shi H., Zhong S., Zhao S., Jiao X., Xin J., Yin X., Liu S. (2025). Nonviral targeted mRNA delivery: Principles, progresses, and challenges. MedComm.

[159] Kiaie S.H., Majidi Zolbanin N., Ahmadi A., Bagherifar R., Valizadeh H., Kashanchi F., Jafari R. (2022). Recent advances in mRNA-LNP therapeutics: Immunological and pharmacological aspects. J. Nanobiotechnol..

[160] Ju Y., Lee W.S., Pilkington E.H., Kelly H.G., Li S., Selva K.J., Wragg K.M., Subbarao K., Nguyen T.H.O., Rowntree L.C. (2022). Anti-PEG Antibodies Boosted in Humans by SARS-CoV-2 Lipid Nanoparticle mRNA Vaccine. ACS Nano.

[161] Omata D., Kawahara E., Munakata L., Tanaka H., Akita H., Yoshioka Y., Suzuki R. (2024). Effect of Anti-PEG Antibody on Immune Response of mRNA-Loaded Lipid Nanoparticles. Mol. Pharm..

[162] Münter R., Christensen E., Andresen T.L., Larsen J.B. (2023). Studying how administration route and dose regulates antibody generation against LNPs for mRNA delivery with single-particle resolution. Mol. Ther. Methods Clin. Dev..

[163] Li M., Ma S., Shen M., Wan R., Long J.A., Jiang Y., Liang H., Guo Z., Ma X., Song W. (2026). Poly (2-oxazoline) decorated lipid nanoparticles for robust mRNA delivery in the presence of pre-existing anti-PEG antibodies. Sci. China Mater..

[164] Xiao Y., Lian X., Sun Y., Sung Y.C., Vaidya A., Chen Z., Gupta A., Chatterjee S., Zheng L., Guerrero E. (2025). High-density brush-shaped polymer lipids reduce anti-PEG antibody binding for repeated administration of mRNA therapeutics. Nat. Mater..

[165] Lokugamage M.P., Vanover D., Beyersdorf J., Hatit M.Z.C., Rotolo L., Echeverri E.S., Peck H.E., Ni H., Yoon J.K., Kim Y. (2021). Optimization of lipid nanoparticles for the delivery of nebulized therapeutic mRNA to the lungs. Nat. Biomed. Eng..

[166] Suberi A., Grun M.K., Mao T., Israelow B., Reschke M., Grundler J., Akhtar L., Lee T., Shin K., Piotrowski-Daspit A.S. (2023). Polymer nanoparticles deliver mRNA to the lung for mucosal vaccination. Sci. Transl. Med..

[167] Baldeon Vaca G., Meyer M., Cadete A., Hsiao C.J., Golding A., Jeon A., Jacquinet E., Azcue E., Guan C.M., Sanchez-Felix X. (2023). Intranasal mRNA-LNP vaccination protects hamsters from SARS-CoV-2 infection. Sci. Adv..

[168] Ongun M., Lokras A.G., Baghel S., Shi Z., Schmidt S.T., Franzyk H., Rades T., Sebastiani F., Thakur A., Foged C. (2024). Lipid nanoparticles for local delivery of mRNA to the respiratory tract: Effect of PEG-lipid content and administration route. Eur. J. Pharm. Biopharm..

[169] Bai X., Chen Q., Li F., Teng Y., Tang M., Huang J., Xu X., Zhang X.Q. (2024). Optimized inhaled LNP formulation for enhanced treatment of idiopathic pulmonary fibrosis via mRNA-mediated antibody therapy. Nat. Commun..

[170] Li B., Jiang A.Y., Raji I., Atyeo C., Raimondo T.M., Gordon A.G.R., Rhym L.H., Samad T., MacIsaac C., Witten J. (2025). Enhancing the immunogenicity of lipid-nanoparticle mRNA vaccines by adjuvanting the ionizable lipid and the mRNA. Nat. Biomed. Eng..

[171] Vu M.N., Pilapitiya D., Kelly A., Koutsakos M., Kent S.J., Juno J.A., Tan H.X., Wheatley A.K. (2025). Deconvolution of cargo delivery and immunogenicity following intranasal delivery of mRNA lipid nanoparticle vaccines. Mol. Ther. Nucleic Acids.

[172] Legere R.M., Ott J.A., Poveda C., Vanover D., Borba K.E.R., Yeon Joo J., Martin C.L., da Silveira B.P., Bray J.M., Landrock K. (2025). Nebulization of an mRNA-encoded monoclonal antibody for passive immunization of foals against Rhodococcus equi. Mol. Ther..

[173] Qiu M., Tang Y., Chen J., Muriph R., Ye Z., Huang C., Evans J., Henske E.P., Xu Q. (2022). Lung-selective mRNA delivery of synthetic lipid nanoparticles for the treatment of pulmonary lymphangioleiomyomatosis. Proc. Natl. Acad. Sci. USA.

[174] Tai W., Yang K., Liu Y., Li R., Feng S., Chai B., Zhuang X., Qi S., Shi H., Liu Z. (2023). A lung-selective delivery of mRNA encoding broadly neutralizing antibody against SARS-CoV-2 infection. Nat. Commun..

[175] Zhang Y., Tian C., Yu X., Yu G., Han X., Wang Y., Zhou H., Zhang S., Li M., Yang T. (2024). Lung-Selective Delivery of mRNA-Encoding Anti-MERS-CoV Nanobody Exhibits Neutralizing Activity Both In Vitro and In Vivo. Vaccines.

[176] Huang J., Bai X., Stewart W., Xu X., Zhang X.Q. (2025). Budesonide-incorporated inhalable lipid nanoparticles for antiTSLP nanobody mRNA delivery to treat steroid-resistant asthma. Nat. Commun..

[177] Tiwari P.M., Vanover D., Lindsay K.E., Bawage S.S., Kirschman J.L., Bhosle S., Lifland A.W., Zurla C., Santangelo P.J. (2018). Engineered mRNA-expressed antibodies prevent respiratory syncytial virus infection. Nat. Commun..

[178] Lindsay K.E., Vanover D., Thoresen M., King H., Xiao P., Badial P., Araínga M., Park S.B., Tiwari P.M., Peck H.E. (2020). Aerosol Delivery of Synthetic mRNA to Vaginal Mucosa Leads to Durable Expression of Broadly Neutralizing Antibodies against HIV. Mol. Ther..

[179] Rotolo L., Vanover D., Bruno N.C., Peck H.E., Zurla C., Murray J., Noel R.K., O’Farrell L., Araínga M., Orr-Burks N. (2023). Species-agnostic polymeric formulations for inhalable messenger RNA delivery to the lung. Nat. Mater..

[180] Joo J.Y., Xiao P., John S.P., Vanover D., Peck H.E., Sasser L.E., Bose D., Jung Y., Hwang J., Zurla C. (2025). Intravaginal delivery of mRNA-encoded antibodies with enhanced breadth and potency for SHIV/HIV protection. Nat. Commun..

[181] Chavalparit T., Barry C., Gunter H., Gillard M., Mercer T., Marcellin E. (2026). Rapid expression of therapeutic antibodies in mammalian cells via mRNA transfection. mAbs.

[182] Campanile E., Pettinà E., Giampiccolo S., Leonardelli L., Marchetti L. (2025). Physiologically Based Pharmacokinetic Modeling of mRNA-Encoded Therapeutics: A Multiscale Framework for LNP and Antibody Trafficking in Mice. bioRxiv.

[183] Pyzik M., Sand K.M.K., Hubbard J.J., Andersen J.T., Sandlie I., Blumberg R.S. (2019). The Neonatal Fc Receptor (FcRn): A Misnomer?. Front. Immunol..

[184] Fiandaca G., Campanile E., Leonardelli L., Pettinà E., Giampiccolo S., Carstens E.J., Dasti L., Zangani N., Marchetti L. (2025). A multiscale physiologically based pharmacokinetic model to support mRNA-encoded BiTE therapy in cancer treatment. Mol. Ther. Nucleic Acids.

[185] Hsu F.F., Liang K.H., Kumari M., Chen W.Y., Lin H.T., Cheng C.M., Tao M.H., Wu H.C. (2022). An efficient approach for SARS-CoV-2 monoclonal antibody production via modified mRNA-LNP immunization. Int. J. Pharm..

[186] Ren P., Peng L., Yang L., Suzuki K., Fang Z., Renauer P.A., Lin Q., Bai M., Li T., Clark P. (2023). RAMIHM generates fully human monoclonal antibodies by rapid mRNA immunization of humanized mice and BCR-seq. Cell Chem. Biol..

[187] An Z., Zhang Y., Yu X., Xia J., Yin Y., Li G., Lu J., Fan X., Xu Y. (2023). The Screening of Broadly Neutralizing Antibodies Targeting the SARS-CoV-2 Spike Protein by mRNA Immunization in Mice. Pharmaceutics.

[188] Liu E.S., Ho K.W., Wu C.C., Fan H.L., Wang T.Y., Hsieh Y.C., Huang B.C., Hong S.T., Liao T.Y., Liu Y.L. (2025). To generate functional anti-protease-activated receptor-4 (PAR4), a G protein-coupled receptor, antibodies through PAR4-mRNA-LNP immunization. npj Vaccines.

[189] Zhang C., Liu Y., Zhao G., Hu B., Xu L., Liu J., Sun Y., Guo X., Deng X., Lian S. (2025). Preparation of Monoclonal Antibodies Against the gD Protein of Feline Herpesvirus Type-1 by mRNA Immunization. Vet. Sci..

[190] Sun H., Miao Y., Yang X., Guo L., Li Q., Wang J., Long J., Zhang Z., Shi J., Li J. (2024). Rapid identification of A29L antibodies based on mRNA immunization and high-throughput single B cell sequencing to detect Monkeypox virus. Emerg. Microbes Infect..

[191] Guenaga J., Alirezaei M., Feng Y., Alameh M.G., Lee W.H., Baboo S., Cluff J., Wilson R., Bale S., Ozorowski G. (2024). mRNA lipid nanoparticles expressing cell-surface cleavage independent HIV Env trimers elicit autologous tier-2 neutralizing antibodies. Front. Immunol..

[192] Lu R.M., Liang K.H., Chiang H.L., Hsu F.F., Lin H.T., Chen W.Y., Ke F.Y., Kumari M., Chou Y.C., Tao M.H. (2023). Broadly neutralizing antibodies against Omicron variants of SARS-CoV-2 derived from mRNA-lipid nanoparticle-immunized mice. Heliyon.

[193] Chen X., Zaro J.L., Shen W.C. (2013). Fusion protein linkers: Property, design and functionality. Adv. Drug Deliv. Rev..

[194] Kim J.H., Lee S.R., Li L.H., Park H.J., Park J.H., Lee K.Y., Kim M.K., Shin B.A., Choi S.Y. (2011). High cleavage efficiency of a 2A peptide derived from porcine teschovirus-1 in human cell lines, zebrafish and mice. PLoS ONE.

[195] Spiess C., Merchant M., Huang A., Zheng Z., Yang N.Y., Peng J., Ellerman D., Shatz W., Reilly D., Yansura D.G. (2013). Bispecific antibodies with natural architecture produced by co-culture of bacteria expressing two distinct half-antibodies. Nat. Biotechnol..

[196] Kontermann R.E., Brinkmann U. (2015). Bispecific antibodies. Drug Discov. Today.

[197] Thakur A., Huang M., Lum L.G. (2018). Bispecific antibody based therapeutics: Strengths and challenges. Blood Rev..

[198] Suurs F.V., Lub-de Hooge M.N., de Vries E.G.E., de Groot D.J.A. (2019). A review of bispecific antibodies and antibody constructs in oncology and clinical challenges. Pharmacol. Ther..

[199] Vanover D., Zurla C., Peck H.E., Orr-Burks N., Joo J.Y., Murray J., Holladay N., Hobbs R.A., Jung Y., Chaves L.C.S. (2022). Nebulized mRNA-Encoded Antibodies Protect Hamsters from SARS-CoV-2 Infection. Adv. Sci..

[200] Du Y., Pattnaik A.K., Song C., Yoo D., Li G. (2012). Glycosyl-phosphatidylinositol (GPI)-anchored membrane association of the porcine reproductive and respiratory syndrome virus GP4 glycoprotein and its co-localization with CD163 in lipid rafts. Virology.

[201] Nelson A.L. (2010). Antibody fragments: Hope and hype. mAbs.

[202] Hamers-Casterman C., Atarhouch T., Muyldermans S., Robinson G., Hamers C., Songa E.B., Bendahman N., Hamers R. (1993). Naturally occurring antibodies devoid of light chains. Nature.

[203] van der Linden R.H., Frenken L.G., de Geus B., Harmsen M.M., Ruuls R.C., Stok W., De Ron L., Wilson S., Davis P., Verrips C.T. (1999). Comparison of physical chemical properties of llama VHH antibody fragments and mouse monoclonal antibodies. Biochim. Biophys. Acta.

[204] Harmsen M.M., De Haard H.J. (2007). Properties, production, and applications of camelid single-domain antibody fragments. Appl. Microbiol. Biotechnol..

[205] Muyldermans S. (2013). Nanobodies: Natural single-domain antibodies. Annu. Rev. Biochem..

[206] Muyldermans S. (2021). Applications of Nanobodies. Annu. Rev. Anim. Biosci..

[207] Narayanan E., Falcone S., Elbashir S.M., Attarwala H., Hassett K., Seaman M.S., Carfi A., Himansu S. (2022). Rational Design and In Vivo Characterization of mRNA-Encoded Broadly Neutralizing Antibody Combinations against HIV-1. Antibodies.

[208] Van Hoecke L., Verbeke R., De Vlieger D., Dewitte H., Roose K., Van Nevel S., Krysko O., Bachert C., Schepens B., Lentacker I. (2020). mRNA Encoding a Bispecific Single Domain Antibody Construct Protects against Influenza A Virus Infection in Mice. Mol. Ther. Nucleic Acids.

[209] Rouzeau R., Schmidt H.R., Deal C.E., Allen J.D., Dudley D.M., Burton I., Rakasz E.G., Plante O., Crispin M., Carfi A. (2026). Harnessing mRNA for the expression of monoclonal IgG and IgA in non-human primates. Front. Immunol..

[210] Nguyen T., Gebo C., Lu J., Popoola D.O., Thomas S.J., Li Y., Waickman A.T. (2025). Development and optimization of an mRNA-vectored single-chain IgA1 isotype monoclonal antibody with potential to treat or prevent dengue virus infection. Antivir. Res..

[211] Deal C.E., Richards A.F., Yeung T., Maron M.J., Wang Z., Lai Y.T., Fritz B.R., Himansu S., Narayanan E., Liu D. (2023). An mRNA-based platform for the delivery of pathogen-specific IgA into mucosal secretions. Cell Rep. Med..

[212] Geall A.J., Verma A., Otten G.R., Shaw C.A., Hekele A., Banerjee K., Cu Y., Beard C.W., Brito L.A., Krucker T. (2012). Nonviral delivery of self-amplifying RNA vaccines. Proc. Natl. Acad. Sci. USA.

[213] Kunyk D., Plotnikova M., Bespalov M., Shevyrev D., Klotchenko S., Ivanov R., Reshetnikov V. (2025). The Interplay Between Therapeutic Self-Amplifying RNA and the Innate Immune System: Balancing Efficiency and Reactogenicity. Int. J. Mol. Sci..

[214] Erasmus J.H., Archer J., Fuerte-Stone J., Khandhar A.P., Voigt E., Granger B., Bombardi R.G., Govero J., Tan Q., Durnell L.A. (2020). Intramuscular Delivery of Replicon RNA Encoding ZIKV-117 Human Monoclonal Antibody Protects against Zika Virus Infection. Mol. Ther. Methods Clin. Dev..

[215] Li J.Q., Zhang Z.R., Zhang H.Q., Zhang Y.N., Zeng X.Y., Zhang Q.Y., Deng C.L., Li X.D., Zhang B., Ye H.Q. (2021). Intranasal delivery of replicating mRNA encoding neutralizing antibody against SARS-CoV-2 infection in mice. Signal Transduct. Target. Ther..

[216] Zhang Y.N., Zhang H.Q., Wang G.F., Zhang Z.R., Li J.Q., Chen X.L., Hu Y.Y., Zeng X.Y., Shi Y.J., Wang J. (2023). Intranasal delivery of replicating mRNA encoding hACE2-targeting antibody against SARS-CoV-2 Omicron infection in the hamster. Antiviral Res..

[217] Liu X., Zhang Y., Zhou S., Dain L., Mei L., Zhu G. (2022). Circular RNA: An emerging frontier in RNA therapeutic targets, RNA therapeutics, and mRNA vaccines. J. Control. Release.

[218] Loan Young T., Chang Wang K., James Varley A., Li B. (2023). Clinical delivery of circular RNA: Lessons learned from RNA drug development. Adv. Drug Deliv. Rev..

[219] O’Leary E., Jiang Y., Kristensen L.S., Hansen T.B., Kjems J. (2025). The therapeutic potential of circular RNAs. Nat. Rev. Genet..

[220] Chen K., Xu Y., Li J., Gu S., Wang Z., Li J., Zhang Y. (2025). The potential and challenges of circular RNA in the development of vaccines and drugs for emerging infectious diseases. Mol. Ther. Nucleic Acids.

[221] Biocca S., Neuberger M.S., Cattaneo A. (1990). Expression and targeting of intracellular antibodies in mammalian cells. EMBO J..

[222] Richardson J.H., Marasco W.A. (1995). Intracellular antibodies: Development and therapeutic potential. Trends Biotechnol..

[223] Lo A.S., Zhu Q., Marasco W.A. (2008). Intracellular antibodies (intrabodies) and their therapeutic potential. Ther. Antibodies.

[224] Slastnikova T.A., Ulasov A.V., Rosenkranz A.A., Sobolev A.S. (2018). Targeted Intracellular Delivery of Antibodies: The State of the Art. Front. Pharmacol..

[225] Rondon I.J., Marasco W.A. (1997). Intracellular antibodies (intrabodies) for gene therapy of infectious diseases. Annu. Rev. Microbiol..

[226] Tanaka T., Lobato M.N., Rabbitts T.H. (2003). Single domain intracellular antibodies: A minimal fragment for direct in vivo selection of antigen-specific intrabodies. J. Mol. Biol..

[227] Böldicke T. (2017). Single domain antibodies for the knockdown of cytosolic and nuclear proteins. Protein Sci..

[228] Rabbitts T.H. (2023). Intracellular Antibodies for Drug Discovery and as Drugs of the Future. Antibodies.

[229] Zhou X., Hao R., Chen C., Su Z., Zhao L., Luo Z., Xie W. (2020). Rapid Delivery of Nanobodies/VHHs into Living Cells via Expressing In Vitro-Transcribed mRNA. Mol. Ther. Methods Clin. Dev..

[230] Blavier J., Esposito G., Twizere J.C., Percipalle P. (2025). Targeting viral replication complexes with mRNA-encoded nanobodies: A new frontier for antiviral design. Drug Discov. Today.

[231] Yan Q., Duan N., Lin M., Zhang W., Denton S., Zhong Y., Dong Y., Rikihisa Y. (2025). Development of mRNA-lipid nanoparticle intrabodies against rickettsial infection. J. Biomed. Sci..

[232] Duan N., Lin M., Zhang W., Yan Q., Chien R.C., Budachetri K., Denton S., Kawahara J., Lakritz J., Zhong Y. (2025). Development of Etf-3-specific nanobodies to prevent Ehrlichia infection and LNP-mRNA delivery in cellular and murine models. Microbiol. Res..

[233] Patel A., Bah M.A., Weiner D.B. (2020). In Vivo Delivery of Nucleic Acid-Encoded Monoclonal Antibodies. BioDrugs.

[234] Chung C., Kudchodkar S.B., Chung C.N., Park Y.K., Xu Z., Pardi N., Abdel-Mohsen M., Muthumani K. (2023). Expanding the Reach of Monoclonal Antibodies: A Review of Synthetic Nucleic Acid Delivery in Immunotherapy. Antibodies.

[235] Zhao Y., Gan L., Ke D., Chen Q., Fu Y. (2023). Mechanisms and research advances in mRNA antibody drug-mediated passive immunotherapy. J. Transl. Med..

[236] Sun W., Wu Y., Ying T. (2024). Progress in novel delivery technologies to improve efficacy of therapeutic antibodies. Antivir. Res..

[237] Köhler G., Milstein C. (1975). Continuous cultures of fused cells secreting antibody of predefined specificity. Nature.

[238] Nadler L.M., Stashenko P., Hardy R., Kaplan W.D., Button L.N., Kufe D.W., Antman K.H., Schlossman S.F. (1980). Serotherapy of a patient with a monoclonal antibody directed against a human lymphoma-associated antigen. Cancer Res..

[239] Miller R.A., Levy R. (1981). Response of cutaneous T cell lymphoma to therapy with hybridoma monoclonal antibody. Lancet.

[240] Ritz J., Schlossman S.F. (1982). Utilization of monoclonal antibodies in the treatment of leukemia and lymphoma. Blood.

[241] Miller R.A., Oseroff A.R., Stratte P.T., Levy R. (1983). Monoclonal antibody therapeutic trials in seven patients with T-cell lymphoma. Blood.

[242] Cobbold S.P., Jayasuriya A., Nash A., Prospero T.D., Waldmann H. (1984). Therapy with monoclonal antibodies by elimination of T-cell subsets in vivo. Nature.

[243] Burke B., Warren G. (1984). Microinjection of mRNA coding for an anti-Golgi antibody inhibits intracellular transport of a viral membrane protein. Cell.

[244] Baron M.D., Garoff H. (1990). Mannosidase II and the 135-kDa Golgi-specific antigen recognized monoclonal antibody 53FC3 are the same dimeric protein. J. Biol. Chem..

[245] Wolff J.A., Malone R.W., Williams P., Chong W., Acsadi G., Jani A., Felgner P.L. (1990). Direct gene transfer into mouse muscle in vivo. Science.

[246] Hoerr I., Probst J., Pascolo S. (2008). RNA-Coded Antibody. U.S. Patent.

[247] Wang X., Gao X., Liu C., Yang L., Li L., Rcheulishvili N., Wang Z., Guo J., Yang C., Zheng Y. (2025). Harnessing mRNA-encoded PcrV-targeting monoclonal antibodies for combating antibiotic-resistant P. aeruginosa infections. Mol. Ther..

[248] Kinoshita M., Kawaguchi K., Le N.B., Kainuma A., Sawa T., Uchida S. (2025). Fc-Free Single Chain Antibody mRNA Therapy for Airway Infection of Multidrug-Resistant Pseudomonas Aeruginosa. bioRxiv.

[249] Qiaerxie G., Jiang Y., Li G., Yang Z., Long F., Yu Y., Lu J.S., Du P., Cui Y. (2024). Design and evaluation of mRNA encoding recombinant neutralizing antibodies for botulinum neurotoxin type B intoxication prophylaxis. Hum. Vaccin. Immunother..

[250] Panova E.A., Kleymenov D.A., Shcheblyakov D.V., Bykonia E.N., Mazunina E.P., Dzharullaeva A.S., Zolotar A.N., Derkaev A.A., Esmagambetov I.B., Sorokin I.I. (2023). Single-domain antibody delivery using an mRNA platform protects against lethal doses of botulinum neurotoxin A. Front. Immunol..

[251] Mukherjee J., Ondeck C.A., Tremblay J.M., Archer J., Debatis M., Foss A., Awata J., Erasmus J.H., McNutt P.M., Shoemaker C.B. (2022). Intramuscular delivery of formulated RNA encoding six linked nanobodies is highly protective for exposures to three Botulinum neurotoxin serotypes. Sci. Rep..

[252] Thran M., Pönisch M., Danz H., Horscroft N., Ichtchenko K., Tzipori S., Shoemaker C.B. (2023). Co-administration of an effector antibody enhances the half-life and therapeutic potential of RNA-encoded nanobodies. Sci. Rep..

[253] Doering J.E., Adewunmi Y., Deal C.E., Plante O., Carfi A., Mantis N.J. (2025). Maternal transfer of mRNA LNP-derived, pathogen-specific, monoclonal IgG to suckling mice. bioRxiv.

[254] Wu X., Yang Z.Y., Li Y., Hogerkorp C.M., Schief W.R., Seaman M.S., Zhou T., Schmidt S.D., Wu L., Xu L. (2010). Rational design of envelope identifies broadly neutralizing human monoclonal antibodies to HIV-1. Science.

[255] Pardi N., Secreto A.J., Shan X., Debonera F., Glover J., Yi Y., Muramatsu H., Ni H., Mui B.L., Tam Y.K. (2017). Administration of nucleoside-modified mRNA encoding broadly neutralizing antibody protects humanized mice from HIV-1 challenge. Nat. Commun..

[256] Kwon Y.D., Georgiev I.S., Ofek G., Zhang B., Asokan M., Bailer R.T., Bao A., Caruso W., Chen X., Choe M. (2016). Optimization of the Solubility of HIV-1-Neutralizing Antibody 10E8 through Somatic Variation and Structure-Based Design. J. Virol..

[257] Burkly L.C., Olson D., Shapiro R., Winkler G., Rosa J.J., Thomas D.W., Williams C., Chisholm P. (1992). Inhibition of HIV infection by a novel CD4 domain 2-specific monoclonal antibody. Dissecting the basis for its inhibitory effect on HIV-induced cell fusion. J. Immunol..

[258] Doria-Rose N.A., Bhiman J.N., Roark R.S., Schramm C.A., Gorman J., Chuang G.Y., Pancera M., Cale E.M., Ernandes M.J., Louder M.K. (2015). New Member of the V1V2-Directed CAP256-VRC26 Lineage That Shows Increased Breadth and Exceptional Potency. J. Virol..

[259] McCoy L.E., Quigley A.F., Strokappe N.M., Bulmer-Thomas B., Seaman M.S., Mortier D., Rutten L., Chander N., Edwards C.J., Ketteler R. (2012). Potent and broad neutralization of HIV-1 by a llama antibody elicited by immunization. J. Exp. Med..

[260] Wang W., Sun L., Li T., Ma Y., Li J., Liu Y., Li M., Wang L., Li C., Xie Y. (2016). A human monoclonal antibody against small envelope protein of hepatitis B virus with potent neutralization effect. mAbs.

[261] Chen B., Chen Y., Li J., Wang C., Song W., Wen Y., Lin J., Wu Y., Ying T. (2022). A Single Dose of Anti-HBsAg Antibody-Encoding mRNA-LNPs Suppressed HBsAg Expression: A Potential Cure of Chronic Hepatitis B Virus Infection. mBio.

[262] Fan P., Chi X., Liu G., Zhang G., Chen Z., Liu Y., Fang T., Li J., Banadyga L., He S. (2020). Potent neutralizing monoclonal antibodies against Ebola virus isolated from vaccinated donors. mAbs.

[263] Fan P., Sun B., Liu Z., Fang T., Ren Y., Zhao X., Song Z., Yang Y., Li J., Yu C. (2024). A pan-orthoebolavirus neutralizing antibody encoded by mRNA effectively prevents virus infection. Emerg. Microbes Infect..

[264] Wolffe E.J., Vijaya S., Moss B. (1995). A myristylated membrane protein encoded by the vaccinia virus L1R open reading frame is the target of potent neutralizing monoclonal antibodies. Virology.

[265] Chen Z., Earl P., Americo J., Damon I., Smith S.K., Zhou Y.H., Yu F., Sebrell A., Emerson S., Cohen G. (2006). Chimpanzee/human mAbs to vaccinia virus B5 protein neutralize vaccinia and smallpox viruses and protect mice against vaccinia virus. Proc. Natl. Acad. Sci. USA.

[266] Chen Z., Earl P., Americo J., Damon I., Smith S.K., Yu F., Sebrell A., Emerson S., Cohen G., Eisenberg R.J. (2007). Characterization of chimpanzee/human monoclonal antibodies to vaccinia virus A33 glycoprotein and its variola virus homolog in vitro and in a vaccinia virus mouse protection model. J. Virol..

[267] Mucker E.M., Thiele-Suess C., Baumhof P., Hooper J.W. (2022). Lipid nanoparticle delivery of unmodified mRNAs encoding multiple monoclonal antibodies targeting poxviruses in rabbits. Mol. Ther. Nucleic Acids.

[268] Chi H., Zhao S.Q., Chen R.Y., Suo X.X., Zhang R.R., Yang W.H., Zhou D.S., Fang M., Ying B., Deng Y.Q. (2024). Rapid development of double-hit mRNA antibody cocktail against orthopoxviruses. Signal Transduct. Target. Ther..

[269] Prosniak M., Faber M., Hanlon C.A., Rupprecht C.E., Hooper D.C., Dietzschold B. (2003). Development of a cocktail of recombinant-expressed human rabies virus-neutralizing monoclonal antibodies for postexposure prophylaxis of rabies. J. Infect. Dis..

[270] Thran M., Mukherjee J., Pönisch M., Fiedler K., Thess A., Mui B.L., Hope M.J., Tam Y.K., Horscroft N., Heidenreich R. (2017). mRNA mediates passive vaccination against infectious agents, toxins, and tumors. EMBO Mol. Med..

[271] Zhao G., He L., Sun S., Qiu H., Tai W., Chen J., Li J., Chen Y., Guo Y., Wang Y. (2018). A Novel Nanobody Targeting Middle East Respiratory Syndrome Coronavirus (MERS-CoV) Receptor-Binding Domain Has Potent Cross-Neutralizing Activity and Protective Efficacy against MERS-CoV. J. Virol..

[272] Fan P., Sun M., Zhang X., Zhang H., Liu Y., Yao Y., Li M., Fang T., Sun B., Chen Z. (2024). A potent Henipavirus cross-neutralizing antibody reveals a dynamic fusion-triggering pattern of the G-tetramer. Nat. Commun..

[273] Liu Z., Sun B., Fang T., Zhao X., Ren Y., Song Z., He S., Li J., Fan P., Yu C. (2025). Characterization of an mRNA-Encoded Antibody Against Henipavirus. Curr. Issues Mol. Biol..

[274] Waickman A.T., Gromowski G.D., Rutvisuttinunt W., Li T., Siegfried H., Victor K., Kuklis C., Gomootsukavadee M., McCracken M.K., Gabriel B. (2020). Transcriptional and clonal characterization of B cell plasmablast diversity following primary and secondary natural DENV infection. EBioMedicine.

[275] Kose N., Fox J.M., Sapparapu G., Bombardi R., Tennekoon R.N., de Silva A.D., Elbashir S.M., Theisen M.A., Humphris-Narayanan E., Ciaramella G. (2019). A lipid-encapsulated mRNA encoding a potently neutralizing human monoclonal antibody protects against chikungunya infection. Sci. Immunol..

[276] August A., Attarwala H.Z., Himansu S., Kalidindi S., Lu S., Pajon R., Han S., Lecerf J.M., Tomassini J.E., Hard M. (2021). A phase 1 trial of lipid-encapsulated mRNA encoding a monoclonal antibody with neutralizing activity against Chikungunya virus. Nat. Med..

[277] Sapparapu G., Fernandez E., Kose N., Cao B., Fox J.M., Bombardi R.G., Zhao H., Nelson C.A., Bryan A.L., Barnes T. (2016). Neutralizing human antibodies prevent Zika virus replication and fetal disease in mice. Nature.

[278] Chen W., Li J., Hao M., Yu C., Hou L., Bian T., Chen Y., Fang T., Liu S. (2023). Monoclonal Antibody A38 Against Rift Valley Fever Virus Use. U.S. Patent.

[279] Wang S., Zhu Z., Li J. (2024). Pharmacokinetic Analyses of a Lipid Nanoparticle-Encapsulated mRNA-Encoded Antibody against Rift Valley Fever Virus. Mol. Pharm..

[280] Kim K.H., Kim J., Ko M., Chun J.Y., Kim H., Kim S., Min J.Y., Park W.B., Oh M.D., Chung J. (2019). An anti-Gn glycoprotein antibody from a convalescent patient potently inhibits the infection of severe fever with thrombocytopenia syndrome virus. PLoS Pathog..

[281] Lee S.Y., Lee Y., Oh E.Y., Lee J., Kim J.Y., Park S.I., Park H.J., Park S.H., Choi E.J., Ha D. (2025). The therapeutic potential of mRNA-encoded SFTSV human monoclonal antibody encapsulated lipid nanoparticle in vivo. J. Control. Release.

[282] Sabnis S., Kumarasinghe E.S., Salerno T., Mihai C., Ketova T., Senn J.J., Lynn A., Bulychev A., McFadyen I., Chan J. (2018). A Novel Amino Lipid Series for mRNA Delivery: Improved Endosomal Escape and Sustained Pharmacology and Safety in Non-human Primates. Mol. Ther..

[283] De Vlieger D., Hoffmann K., Van Molle I., Nerinckx W., Van Hoecke L., Ballegeer M., Creytens S., Remaut H., Hengel H., Schepens B. (2019). Selective Engagement of FcγRIV by a M2e-Specific Single Domain Antibody Construct Protects Against Influenza A Virus Infection. Front. Immunol..

[284] Rossey I., Gilman M.S., Kabeche S.C., Sedeyn K., Wrapp D., Kanekiyo M., Chen M., Mas V., Spitaels J., Melero J.A. (2017). Potent single-domain antibodies that arrest respiratory syncytial virus fusion protein in its prefusion state. Nat. Commun..

[285] Vanderven H.A., Esterbauer R., Jegaskanda S., Tan H.X., Wheatley A.K., Kent S.J. (2022). Poor protective potential of influenza nucleoprotein antibodies despite wide prevalence. Immunol. Cell Biol..

[286] Vu M.N., Neil J.A., Mackenzie-Kludas C., Kelly A., Tan H.X., Subbarao K., Lee W.S., Wheatley A.K. (2025). De novo administration of antiviral monoclonal antibodies against SARS-CoV-2 or influenza using mRNA lipid nanoparticles. bioRxiv.

[287] Dreyfus C., Laursen N.S., Kwaks T., Zuijdgeest D., Khayat R., Ekiert D.C., Lee J.H., Metlagel Z., Bujny M.V., Jongeneelen M. (2012). Highly conserved protective epitopes on influenza B viruses. Science.

[288] Shi R., Shan C., Duan X., Chen Z., Liu P., Song J., Song T., Bi X., Han C., Wu L. (2020). A human neutralizing antibody targets the receptor-binding site of SARS-CoV-2. Nature.

[289] Chen Y., Zhang Y.N., Yan R., Wang G., Zhang Y., Zhang Z.R., Li Y., Ou J., Chu W., Liang Z. (2021). ACE2-targeting monoclonal antibody as potent and broad-spectrum coronavirus blocker. Signal Transduct. Target. Ther..

[290] Zhu L., Deng Y.Q., Zhang R.R., Cui Z., Sun C.Y., Fan C.F., Xing X., Huang W., Chen Q., Zhang N.N. (2020). Double lock of a potent human therapeutic monoclonal antibody against SARS-CoV-2. Natl. Sci. Rev..

[291] Deng Y.Q., Zhang N.N., Zhang Y.F., Zhong X., Xu S., Qiu H.Y., Wang T.C., Zhao H., Zhou C., Zu S.L. (2022). Lipid nanoparticle-encapsulated mRNA antibody provides long-term protection against SARS-CoV-2 in mice and hamsters. Cell Res..

[292] Wang L., Fu W., Bao L., Jia Z., Zhang Y., Zhou Y., Wu W., Wu J., Zhang Q., Gao Y. (2022). Selection and structural bases of potent broadly neutralizing antibodies from 3-dose vaccinees that are highly effective against diverse SARS-CoV-2 variants, including Omicron sublineages. Cell Res..

[293] Chi H., Zhao S.Q., Liu Y.P., Xiong X.C., Zhong X., Suo X.X., Huang X.Y., Yue C., Zhang J.J., Cong Z. (2025). Efficacy and safety of an mRNA encoding neutralizing antibody against SARS-CoV-2 in non-human primates. Sci. Bull..

[294] Zost S.J., Gilchuk P., Case J.B., Binshtein E., Chen R.E., Nkolola J.P., Schäfer A., Reidy J.X., Trivette A., Nargi R.S. (2020). Potently neutralizing and protective human antibodies against SARS-CoV-2. Nature.

[295] Li D., Edwards R.J., Manne K., Martinez D.R., Schäfer A., Alam S.M., Wiehe K., Lu X., Parks R., Sutherland L.L. (2021). In vitro and in vivo functions of SARS-CoV-2 infection-enhancing and neutralizing antibodies. Cell.

[296] Esposito G., Hunashal Y., Percipalle M., Venit T., Dieng M.M., Fogolari F., Hassanzadeh G., Piano F., Gunsalus K.C., Idaghdour Y. (2021). NMR-Based Analysis of Nanobodies to SARS-CoV-2 Nsp9 Reveals a Possible Antiviral Strategy Against COVID-19. Adv. Biol..

[297] Venit T., Blavier J., Maseko S.B., Shu S., Espada L., Breunig C., Holthoff H.P., Desbordes S.C., Lohse M., Esposito G. (2024). Nanobody against SARS-CoV-2 non-structural protein Nsp9 inhibits viral replication in human airway epithelia. Mol. Ther. Nucleic Acids.

[298] Westendorf K., Žentelis S., Wang L., Foster D., Vaillancourt P., Wiggin M., Lovett E., van der Lee R., Hendle J., Pustilnik A. (2022). LY-CoV1404 (bebtelovimab) potently neutralizes SARS-CoV-2 variants. Cell Rep..

[299] Sun X., Yi C., Zhu Y., Ding L., Xia S., Chen X., Liu M., Gu C., Lu X., Fu Y. (2022). Neutralization mechanism of a human antibody with pan-coronavirus reactivity including SARS-CoV-2. Nat. Microbiol..

[300] Hazell N.C., Reyna R.A., Adam A., Bonam S.R., Bei J., Kumar N., Nguyen T.N., Plante J.A., Wu T., Walker D.H. (2026). mRNA-delivered neutralizing antibodies confer protection against SARS-CoV-2 in animal models. J. Virol..

[301] Wheatley A.K., Pymm P., Esterbauer R., Dietrich M.H., Lee W.S., Drew D., Kelly H.G., Chan L.J., Mordant F.L., Black K.A. (2021). Landscape of human antibody recognition of the SARS-CoV-2 receptor binding domain. Cell Rep..

[302] Freed E.O. (2001). HIV-1 replication. Somat. Cell Mol. Genet..

[303] Stevenson M. (2003). HIV-1 pathogenesis. Nat. Med..

[304] Seeger C., Mason W.S. (2015). Molecular biology of hepatitis B virus infection. Virology.

[305] Seto W.K., Lo Y.R., Pawlotsky J.M., Yuen M.F. (2018). Chronic hepatitis B virus infection. Lancet.

[306] Jacob S.T., Crozier I., Fischer W.A., Hewlett A., Kraft C.S., Vega M.A., Soka M.J., Wahl V., Griffiths A., Bollinger L. (2020). Ebola virus disease. Nat. Rev. Dis. Primers.

[307] Shchelkunova G.A., Shchelkunov S.N. (2022). Smallpox, Monkeypox and Other Human Orthopoxvirus Infections. Viruses.

[308] Karagoz A., Tombuloglu H., Alsaeed M., Tombuloglu G., AlRubaish A.A., Mahmoud A., Smajlović S., Ćordić S., Rabaan A.A., Alsuhaimi E. (2023). Monkeypox (mpox) virus: Classification, origin, transmission, genome organization, antiviral drugs, and molecular diagnosis. J. Infect. Public Health.

[309] Lu J., Xing H., Wang C., Tang M., Wu C., Ye F., Yin L., Yang Y., Tan W., Shen L. (2023). Mpox (formerly monkeypox): Pathogenesis, prevention, and treatment. Signal Transduct. Target. Ther..

[310] Schnell M.J., McGettigan J.P., Wirblich C., Papaneri A. (2010). The cell biology of rabies virus: Using stealth to reach the brain. Nat. Rev. Microbiol..

[311] de Wit E., van Doremalen N., Falzarano D., Munster V.J. (2016). SARS and MERS: Recent insights into emerging coronaviruses. Nat. Rev. Microbiol..

[312] Eaton B.T., Broder C.C., Middleton D., Wang L.F. (2006). Hendra and Nipah viruses: Different and dangerous. Nat. Rev. Microbiol..

[313] Singh R.K., Dhama K., Chakraborty S., Tiwari R., Natesan S., Khandia R., Munjal A., Vora K.S., Latheef S.K., Karthik K. (2019). Nipah virus: Epidemiology, pathology, immunobiology and advances in diagnosis, vaccine designing and control strategies—A comprehensive review. Vet. Q..

[314] Martina B.E., Koraka P., Osterhaus A.D. (2009). Dengue virus pathogenesis: An integrated view. Clin. Microbiol. Rev..

[315] Roy S.K., Bhattacharjee S. (2021). Dengue virus: Epidemiology, biology, and disease aetiology. Can. J. Microbiol..

[316] Schwartz O., Albert M.L. (2010). Biology and pathogenesis of chikungunya virus. Nat. Rev. Microbiol..

[317] Silva L.A., Dermody T.S. (2017). Chikungunya virus: Epidemiology, replication, disease mechanisms, and prospective intervention strategies. J. Clin. Investig..

[318] Musso D., Gubler D.J. (2016). Zika Virus. Clin. Microbiol. Rev..

[319] Weaver S.C., Costa F., Garcia-Blanco M.A., Ko A.I., Ribeiro G.S., Saade G., Shi P.Y., Vasilakis N. (2016). Zika virus: History, emergence, biology, and prospects for control. Antiviral Res..

[320] Pepin M., Bouloy M., Bird B.H., Kemp A., Paweska J. (2010). Rift Valley fever virus (Bunyaviridae: Phlebovirus): An update on pathogenesis, molecular epidemiology, vectors, diagnostics and prevention. Vet. Res..

[321] Liu Q., He B., Huang S.Y., Wei F., Zhu X.Q. (2014). Severe fever with thrombocytopenia syndrome, an emerging tick-borne zoonosis. Lancet Infect. Dis..

[322] Casel M.A., Park S.J., Choi Y.K. (2021). Severe fever with thrombocytopenia syndrome virus: Emerging novel phlebovirus and their control strategy. Exp. Mol. Med..

[323] Krammer F., Smith G.J.D., Fouchier R.A.M., Peiris M., Kedzierska K., Doherty P.C., Palese P., Shaw M.L., Treanor J., Webster R.G. (2018). Influenza. Nat. Rev. Dis. Primers.

[324] Griffiths C., Drews S.J., Marchant D.J. (2017). Respiratory Syncytial Virus: Infection, Detection, and New Options for Prevention and Treatment. Clin. Microbiol. Rev..

[325] Naqvi A.A.T., Fatima K., Mohammad T., Fatima U., Singh I.K., Singh A., Atif S.M., Hariprasad G., Hasan G.M., Hassan M.I. (2020). Insights into SARS-CoV-2 genome, structure, evolution, pathogenesis and therapies: Structural genomics approach. Biochim. Biophys. Acta Mol. Basis Dis..

[326] Hu B., Guo H., Zhou P., Shi Z.L. (2021). Characteristics of SARS-CoV-2 and COVID-19. Nat. Rev. Microbiol..

[327] V’kovski P., Kratzel A., Steiner S., Stalder H., Thiel V. (2021). Coronavirus biology and replication: Implications for SARS-CoV-2. Nat. Rev. Microbiol..

[328] Lamers M.M., Haagmans B.L. (2022). SARS-CoV-2 pathogenesis. Nat. Rev. Microbiol..

[329] Dickie P., Felser J., Eckhaus M., Bryant J., Silver J., Marinos N., Notkins A.L. (1991). HIV-associated nephropathy in transgenic mice expressing HIV-1 genes. Virology.

[330] Michaud E., Mastrandrea C., Rochereau N., Paul S. (2020). Human Secretory IgM: An Elusive Player in Mucosal Immunity. Trends Immunol..

[331] Matsumoto M.L. (2022). Molecular Mechanisms of Multimeric Assembly of IgM and IgA. Annu. Rev. Immunol..

[332] Mayer B.T., Zhang L., deCamp A.C., Yu C., Sato A., Angier H., Seaton K.E., Yates N., Ledgerwood J.E., Mayer K. (2024). Impact of LS Mutation on Pharmacokinetics of Preventive HIV Broadly Neutralizing Monoclonal Antibodies: A Cross-Protocol Analysis of 16 Clinical Trials in People without HIV. Pharmaceutics.

[333] Antonelli A.C.B., Rodrigues L.S., Pinheiro M.C.M., Pinhate S.B., Brígido M.D.M., Maranhão A.Q. (2025). Therapeutic Antibodies for Mosquito-Borne Orthoflavivirus Infections: Discovery, Engineering Approaches, and Advances in mRNA-Based Delivery Systems. Adv. Ther..

[334] Safety, Tolerability, Pharmacokinetics, and Pharmacodynamics of mRNA-1944 in Healthy Adults: NCT03829384. NCT03829384.

[335] Frolov I., Hoffman T.A., Prágai B.M., Dryga S.A., Huang H.V., Schlesinger S., Rice C.M. (1996). Alphavirus-based expression vectors: Strategies and applications. Proc. Natl. Acad. Sci. USA.

[336] Lazear H.M., Govero J., Smith A.M., Platt D.J., Fernandez E., Miner J.J., Diamond M.S. (2016). A Mouse Model of Zika Virus Pathogenesis. Cell Host Microbe.

[337] Van den Hoecke S., Ehrhardt K., Kolpe A., El Bakkouri K., Deng L., Grootaert H., Schoonooghe S., Smet A., Bentahir M., Roose K. (2017). Hierarchical and Redundant Roles of Activating FcγRs in Protection against Influenza Disease by M2e-Specific IgG1 and IgG2a Antibodies. J. Virol..

[338] Long J.S., Mistry B., Haslam S.M., Barclay W.S. (2019). Host and viral determinants of influenza A virus species specificity. Nat. Rev. Microbiol..

[339] Huang C., Zeng W., Zhong K., Duan X., Liu Y., Tian Q., Zhou L., Wang Y., Nie C., Tong A. (2025). A novel ionizable lipid nanoparticle platform for circular RNA-encoded PD-L1× CD3 bispecific antibodies elicits potent antitumor immunity. Chin. Chem. Lett..

[340] Mukhtar M.M., Li S., Li W., Wan T., Mu Y., Wei W., Kang L., Rasool S.T., Xiao Y., Zhu Y. (2009). Single-chain intracellular antibodies inhibit influenza virus replication by disrupting interaction of proteins involved in viral replication and transcription. Int. J. Biochem. Cell Biol..

[341] Ashour J., Schmidt F.I., Hanke L., Cragnolini J., Cavallari M., Altenburg A., Brewer R., Ingram J., Shoemaker C., Ploegh H.L. (2015). Intracellular expression of camelid single-domain antibodies specific for influenza virus nucleoprotein uncovers distinct features of its nuclear localization. J. Virol..

[342] Jin Q., Yao Z., Liu F., Di Y., Gao J., Zhang X. (2022). The protective effect of a combination of human intracellular and extracellular antibodies against the highly pathogenic avian influenza H5N1 virus. Hum. Vaccin. Immunother..

[343] Bessonne M., Morel J., Nevers Q., Da Costa B., Ballandras-Colas A., Chenavier F., Grange M., Roussel A., Crépin T., Delmas B. (2024). Antiviral activity of intracellular nanobodies targeting the influenza virus RNA-polymerase core. PLoS Pathog..

[344] Pizzorno A., Padey B., Julien T., Trouillet-Assant S., Traversier A., Errazuriz-Cerda E., Fouret J., Dubois J., Gaymard A., Lescure F.X. (2020). Characterization and Treatment of SARS-CoV-2 in Nasal and Bronchial Human Airway Epithelia. Cell Rep. Med..

[345] Han F., Guo X.-Y., Cui L.-Y., Zhang M.-X., Zeng Y.-R., Wang G.-Q., Li J.-J., Chi X., Jiang M.-X., Xiong Y.-T. (2026). An mRNA-encoded scFv antibody targeting the helix-α3 of HPV18 E7 oncoprotein as a novel antiviral strategy. mBio.

[346] Schiffman M., Doorbar J., Wentzensen N., de Sanjosé S., Fakhry C., Monk B.J., Stanley M.A., Franceschi S. (2016). Carcinogenic human papillomavirus infection. Nat. Rev. Dis. Primers.

[347] Niazi S.K. (2025). Affordable mRNA Novel Proteins, Recombinant Protein Conversions, and Biosimilars-Advice to Developers and Regulatory Agencies. Biomedicines.

[348] Camperi J., Chatla K., Freund E., Galan C., Lippold S., Guilbaud A. (2025). Current Analytical Strategies for mRNA-Based Therapeutics. Molecules.

[349] Whitley J., Zwolinski C., Denis C., Maughan M., Hayles L., Clarke D., Snare M., Liao H., Chiou S., Marmura T. (2022). Development of mRNA manufacturing for vaccines and therapeutics: mRNA platform requirements and development of a scalable production process to support early phase clinical trials. Transl. Res..

[350] Schoenmaker L., Witzigmann D., Kulkarni J.A., Verbeke R., Kersten G., Jiskoot W., Crommelin D.J.A. (2021). mRNA-lipid nanoparticle COVID-19 vaccines: Structure and stability. Int. J. Pharm..

[351] Singh D. (2026). mRNA-Encoded antibodies as a next-generation therapeutic paradigm: A rapid and adaptive platform for the prevention and treatment of emerging and re-emerging infectious diseases—A critical review. Immunol. Res..

[352] Che S., Li Z., Su Z., Li Z., Yu A., Liu M., Zhang S. (2026). Engineering of enzymatic modules for mRNA manufacturing: Advances in catalytic regulation and process integration. Chin. J. Catal..

